# Multistep molecular assessment of pediocin-like bacteriocins as antifungal agents targeting secreted aspartic protease 2 (SAP2) of Candida albicans through computational modeling and molecular dynamics

**DOI:** 10.1007/s40203-026-00642-3

**Published:** 2026-05-26

**Authors:** Iago Rodrigues Blanco, Ricardo Pinheiro de Souza Oliveira, Matheus M. Pereira

**Affiliations:** 1 Department of Biochemical and Pharmaceutical Technology, School of Pharmaceutical Sciences, University of São Paulo, São Paulo, Brazil; 2 Department of Chemical Engineering, University of Coimbra, CERES, Rua Sílvio Lima, Pólo II – Pinhal de Marrocos, 3030-790 Coimbra, Portugal

**Keywords:** Pediocin-like bacteriocins, Secreted aspartic proteases (SAP) inhibitors, Molecular docking, Molecular dynamics

## Abstract

Antimicrobial resistance is a major public health concern with significant future implications. Among potential alternatives for pathogen inhibition, antimicrobial peptides (AMPs), particularly pediocin-like bacteriocins, are noteworthy due to their efficacy and reduced toxicity to hosts and the environment. In this context, peptide repurposing is relevant for the development of strategies against *Candida albicans*, an opportunistic pathogen associated with high mortality and healthcare costs. This study evaluated in silico the interactions between four pediocin-like bacteriocins (Coagulin A, Pediocin PA-1, Penocin A, and Plantaricin 423) and the secreted aspartic protease 2 (SAP2) of *C. albicans*, a validated antifungal target. Through structural modeling, we have generated high-quality models with conserved structural features. Then, molecular docking and MM/GBSA analyses identified Coagulin A and Pediocin PA-1 as the peptides with the most favorable binding energies and energetically coherent interactions with the SAP2 catalytic dyad, with electrostatic and van der Waals interactions predominating across all complexes, respectively. Molecular dynamics analyses, including RMSD, RMSF, Radius of gyration, Solvent-accessible surface area and Binding energy, demonstrated that Coagulin A and Pediocin PA-1 as the peptides with the most favorable binding energies and coherent interactions with the active site residues of SAP2, specifically the catalytic dyad ASP32 and ASP218, stabilizing in low-flexibility conformations, whereas Penocin A induced more solvated and structurally expanded states, and Plantaricin 423 showed intermediate behavior. These findings highlight the potential of Coagulin A and Pediocin PA-1 as SAP2 inhibitors and provide a computational foundation for developing novel antifungal strategies against *C. albican*s.

**Supplementary Information:**

The online version contains supplementary material available at 10.1007/s40203-026-00642-3.

## Introduction

Over recent decades, antimicrobial resistance has emerged as one of the most critical global public health challenges, with serious implications for human, veterinary, and environmental health (Kasimanickam et al. 2021). The indiscriminate use of antibiotics in livestock and agricultural production has driven the proliferation of drug-resistant microorganisms, which are currently associated with millions of deaths each year (Van Boeckel et al. 2015; Antimicrobial Resistance Collaborators 2022). Projections suggest that, by 2050, antimicrobial resistance could surpass cancer as a leading cause of mortality if effective interventions are not implemented (O’neill 2014).

Conversely, the growth of pathogenic microorganisms from diverse genera, many of which cause clinically relevant infections, has been shown to be inhibited by antimicrobial peptides (AMPs). These are generally associated with reduced toxicity to the host and environment (Mahlapuu et al. 2016) and exert their inhibitory activity through various mechanisms, often mediated by bacteriocins – which can induce processes such as cell wall disruption, energy depletion, and interference with essential cellular functions (Héchard and Sahl 2002). Among these, pediocin-like bacteriocins (class IIA) are particularly notable for their anti-Listeria activity. These peptides target the mannose phosphotransferase system (Man-PTS) on the cell membrane, where receptor binding promotes pore formation, ultimately impairing critical cellular processes such as amino acid transport and ion homeostasis, leading to cell death (Ríos Colombo et al. 2018); (Kjos et al. 2011).

The application of AMPs, capable of exerting targeted inhibition against specific microbial groups, is regarded as a promising strategy with significant biotechnological potential (Mahlapuu et al. 2016). Among microorganisms that naturally produce bioactive compounds, probiotics such as *Pediococcus pentosaceus* are particularly relevant. This species synthesizes pediocin-like bacteriocins (small AMPs originally identified in Pediococcus) with demonstrated activity against Gram-positive bacteria, including Listeria, Clostridium, and other susceptible pathogens (Ríos Colombo et al. 2018). Such inhibitory potential has been exploited in various applications, including food preservation, modulation of the gut microbiota, and treatment of gastrointestinal infections (Hernández-González et al. 2021). In contrast, advances in antibacterial strategies have not been paralleled in antifungal drug development, where high toxicity and elevated costs remain major limitations, underscoring the urgent need for new safe and effective therapeutic approaches (Perfect 2017).

Given that the therapeutic effects of bacteriocins extend beyond the inhibition of Gram-positive bacteria, we investigated their potential repurposing for antifungal activity, based on their established bioactive properties. This emerging line of research holds promise for the development of novel antimicrobial strategies with improved efficacy and reduced cost. Secreted aspartic protease 2 (SAP2) is a key enzyme involved in nutrient acquisition, immune modulation, tissue invasion, and pathogenicity in fungal species, including *C. albicans* (Bras et al. 2024). Inhibition of SAP family enzymes through peptidomimetic approaches has been shown to reduce the growth of Candida species both in vitro and in vivo (Pichová et al. 2001). Nevertheless, the clinical application of synthetic SAP inhibitors is frequently hindered by undesirable toxic effects and off-target interactions with host proteases, reinforcing the need for highly specific and biocompatible alternatives such as AMPs (Perfect 2017); (Mahlapuu et al. 2016). However, the ability of pediocin-like bacteriocins to inhibit *C. albicans* remains unreported, despite their structural diversity and broad antimicrobial activity.

*C. albicans* is an opportunistic pathogen and the most prominent fungal species associated with human infections, ranging from superficial skin and mucosal disease to systemic infections affecting multiple organs (Talapko et al. 2021). Its pathogenicity is linked to approximately 200,000 deaths annually, with mortality rates for invasive candidiasis reported between 40% and 55% (Soriano et al. 2023); (Richardson 2022). In the United States alone, healthcare costs related to *C. albicans* infections are estimated at nearly USD 2 billion annually (Soriano et al. 2023); (Richardson 2022). In this context, we evaluated sequences of pediocin-like bacteriocins produced by *P. pentosaceus* for their potential inhibitory activity against this pathogen, proposing their repurposing as antifungal agents. To this end, a multistep molecular analysis using molecular modeling, molecular docking, MM/GBSA, and molecular dynamics were conducted to assess their potential to inhibit SAP2 from *C. albicans*. The exhaustive quantitative mapping of these interactions and the overall structural dynamics are thoroughly detailed in the subsequent results sections.

## Methodology

### Molecular modelling

Pediocin-like peptide sequences identified in *P. pentosaceus* genomes were retrieved from GenBank. The four pediocin-like bacteriocin sequences were subjected to structural modeling using AlphaFold v2 (Jumper et al. 2021), integrated with the HHsearch algorithm and MMseqs2 tool (Steinegger and Söding 2017) for homology searches, all executed using Google ColabFold v1.5.5 platform (Mirdita et al. 2022). Modeling was performed using the pdb100 template mode and Amber relaxation was applied, generating five relaxed and five unrelaxed models, recycled six times. Model quality was assessed through analysis of the Predicted Aligned Error (PAE), the Predicted Local Distance Difference Test (pLDDT), which estimates the confidence of the structural predictions, and the predicted Template Modeling score (pTM), which evaluates the accuracy of the protein fold. These metrics informed the selection of the highest-scoring unrelaxed model for each peptide to be used in subsequent analyses. Sequence coverage for each model was calculated and predicted aligned error (PAE) heatmaps were generated to visualize structural uncertainties. The structures generated for each peptide were exported in PDB format and prepared for subsequent computational analyses. To ensure the reliability of the predicted models, their stereochemical quality and 3D structural environments were validated using structure-based web tools. By SAVES 6.1 structure validation server, the Ramachandran plot was generated using PROCHECK to assess the distribution of the backbone dihedral angles. Additionally, the ERRAT and VERIFY3D tools were employed to analyze the statistics of non-bonded atomic interactions and the compatibility of the 3D atomic model with its own 1D amino acid sequence, respectively. Finally, the overall model quality and deviation from high-resolution structures were estimated by calculating the Z-score using the ProSA-web server, a metric that evaluates the model by measuring its total energy deviation compared to experimentally resolved high-resolution native structures.

### Molecular docking

After structural modeling, the peptides and the receptor (SAP2 from *C. albicans*, PDB ID: 3PVK) were subjected to protonation using the ProteinPrepare server (Martínez-Rosell et al. 2017), adjusted to a pH of 7.4. All protonated PDB files were saved for use in subsequent molecular docking and molecular dynamics analyses. The structures of the modeled peptides were docked to the SAP2 of *C. albicans*, obtained from the Protein Data Bank (RCSB PDB, https://www.rcsb.org/). For this purpose, the previously protonated and cleaned receptor structure was prepared in BIOVIA Discovery Studio 2024 Client. During this step, water molecules, unnecessary cofactors, and any duplicate residues were manually removed. The stereochemical quality of the prepared receptor was validated using the SAVES v6.1 server (PROCHECK, ERRAT, VERIFY 3D) and ProSA-web server. Protein chains were renamed according to their specific designations (chain A: receptor), and the structures of the peptides (ligands) were inserted and assigned as chains B in the corresponding complex files. The prepared structures were saved in PDB format for subsequent steps. Molecular docking was performed using the HDOCK server (Yan et al. 2020), with receptor binding residues (Chain A: 32, 33, 34, 35, 36, 218, 219, 220, and 221) were specified based on experimental data and literature to guide molecular docking (Pranav Kumar and Kulkarni 2002). After running HDOCK, each complex was evaluated based on its docking score, confidence score, and ligand RMSD, and ranked accordingly. The highest-ranked complexes for each peptide–receptor interaction were selected for further analysis. To establish a comparative baseline for binding affinity and interaction profiling, benzamidine, a well-known small molecule inhibitor of SAP2 extracted from the reference crystallographic complex, was redocked into the receptor using the exact same parameters. Interaction profiles for all complexes were mapped using BIOVIA Discovery Studio to pinpoint specific molecular interactions.

### MM/GBSA

As a preliminary static screening to estimate the initial energetic favorability and viability of the peptide-SAP2 complexes, the HawkDock server was utilized (Weng et al. 2019). The docked poses of receptor-ligand complexes generated in the previous step were submitted to compute the static MM/GBSA (Molecular Mechanics/Generalized Born Surface Area) binding free energies, decomposed into van der Waals, electrostatic, polar solvation and nonpolar solvation contributions. This initial filter was essential to identify the thermodynamic potential of the complexes prior to rigorous molecular dynamics evaluations.

### Molecular dynamics simulation

The top-ranked receptor-ligand complex obtained from the molecular docking was used as the starting structure for the molecular dynamics (MD) simulations. The PDB files for each complex were loaded into the YASARA software (Land and Humble 2018) for the simulations. The md_run.mcr macro was employed to execute the simulations, with parameters configured to approximate physiological conditions. Each system was simulated for 100 ns using the AMBER14 force field in a cubic box filled with water (TIP3P model), with the edges spaced 10 Å from the protein surface. The system was neutralized, and energy minimization was performed using a steepest-descent algorithm to optimize the geometry. Furthermore, the pH was adjusted to 7.4, the NaCl concentration to 0.9%, the temperature to 310.15 K (37 °C), and the water density to 0.997 g/mL.

Subsequently, the md_analyze.mcr macro was applied to evaluate the MD trajectories and compute structural and dynamic properties, including Root Mean Square Deviation (RMSD), Root Mean Square Fluctuation (RMSF), Solvent Accessible Surface Area (SASA), and Radius of Gyration (Rg).

To assess for receptor flexibility and solvent effects over time, the dynamic binding free energy was calculated continuously across the 100 ns trajectory using the md_analyzebindenergy.mcr macro in YASARA. This approach computes the total potential energy and the solvation energy utilizing a Poisson-Boltzmann (MM/PBSA-like) or BoundaryFast approach for the complex and isolated components at regular intervals, providing a highly reliable energetic profile compared to static evaluations, and the md_convert.mcr macro was executed to analyze and visualize the complex structures, generating structural representations of the MD trajectory.

## Results and discussion

### Molecular modelling of peptides

Pediocin-like peptide sequences previously identified in *P. pentosaceus* genomes were subjected to three-dimensional structural modeling using AlphaFold v2. Four peptides were analyzed: Coagulin A, Pediocin PA-1, Penocin A and Plantaricin 423. For each peptide, multiple structural models were generated and evaluated using the following quality parameters: Predicted Alignment Error (PAE) and Predicted Local Distance Difference Test (pLDDT), predicted TM-score (PTM) and sequence coverage.

All analyzed peptides exhibited structural elements characteristic of pediocin-like bacteriocins, notably the conserved “pediocin box”. This motif, defined by the sequence “YGNGVxCxxxxCxVxWxxA”, promotes an S-shaped fold in the N-terminal region, a feature known to contribute substantially to the structural stability of these peptides (Rodrigues Blanco et al. 2022). To ensure robustness of the predicted models, all quality parameters were evaluated. The pLDDT values for the peptides ranged from 80.9 to 84.7, indicating a good confidence in the predicted structures and supporting their expected stability (Keskin Karakoyun et al. 2023). In general, pLDDT values > 70 are considered reliable, while values ≥ 90 indicate high confidence in structural prediction (Jumper et al. 2021). Additionally, PAE heat maps were generated to visualize potential structural inconsistencies in specific regions (Figs. S1–S4).

**Fig. 1:**
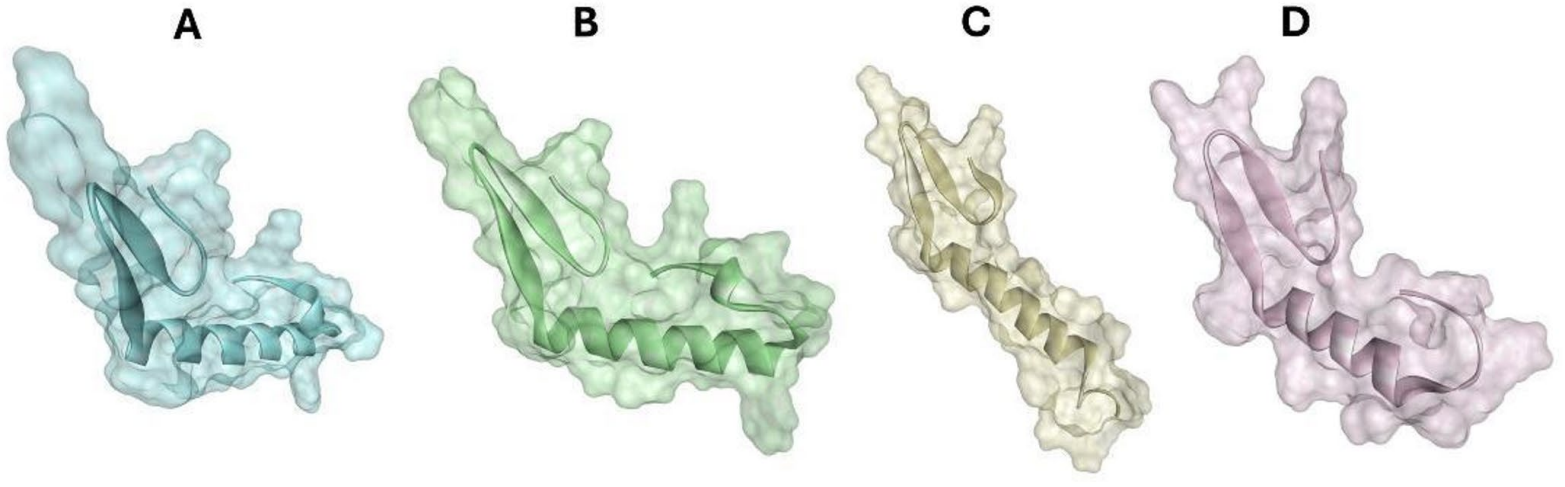
Three-dimensional structures of the peptides** A** Coagulin A,** B** Pediocin PA-1,** C** Penocin A and** D** Plantaricin 423 generated by AlphaFold v2. A surface structure surrounding the protein was created in Discovery Studio

Structural modeling of the four bacteriocins revealed substantial conservation of key features, consistent with a shared mechanism of action and providing a foundation for interpreting their interactions with SAP2. Figure 1 illustrates the three-dimensional structures generated for the four peptides. Among them, Pediocin PA-1 was reported to exhibit the highest structural stability based on stereochemical metrics (Figs. S1–S4 and S6–S9). It achieved the highest percentage of residues in the most favored Ramachandran region (100%), an exceptional ERRAT overall quality factor (100%), and a highly reliable ProSA Z-score (− 1.61), alongside robust pLDDT confidence values. All models demonstrated satisfactory confidence value**s**, confirming the preservation of canonical folds and conserved domains essential for biological activity. PAE profiles revealed low predicted errors in conserved regions, supporting high structural accuracy relative to homologous templates. Collectively, these results validate the reliability of the predicted structures and confirm their suitability for subsequent computational analyses. Structural validation further confirmed the quality of generated models. Ramachandran plot analysis by PROCHECK revealed that all peptides had 100% of their residues in allowed regions. Specifically, Pediocin PA-1 displayed 100% of its residues in the most favored region, followed by Coagulin A with 97.1%, Penocin A with 87.5%, and Plantaricin 423 with 75.9%. The overall quality factor calculated by ERRAT was exceptional for Coagulin A and Pediocin PA-1 (100.0%), very good for Penocin A (96.0%), and acceptable for Plantaricin 423 (76.47%). The Z-scores obtained from ProSA were consistent with native structures of similar sizes, yielding values of − 1.45 for Coagulin A, − 1.61 for Pediocin PA-1, − 0.21 for Penocin A, and − 1.44 for Plantaricin 423. VERIFY3D results additionally corroborated the proper 3D folding of the peptides. These validation profiles ensure the structural viability of these peptides for the subsequent molecular docking and dynamics studies (Figs. S6–S9, Supplementary Material).

### Molecular docking

**Fig. 2:**
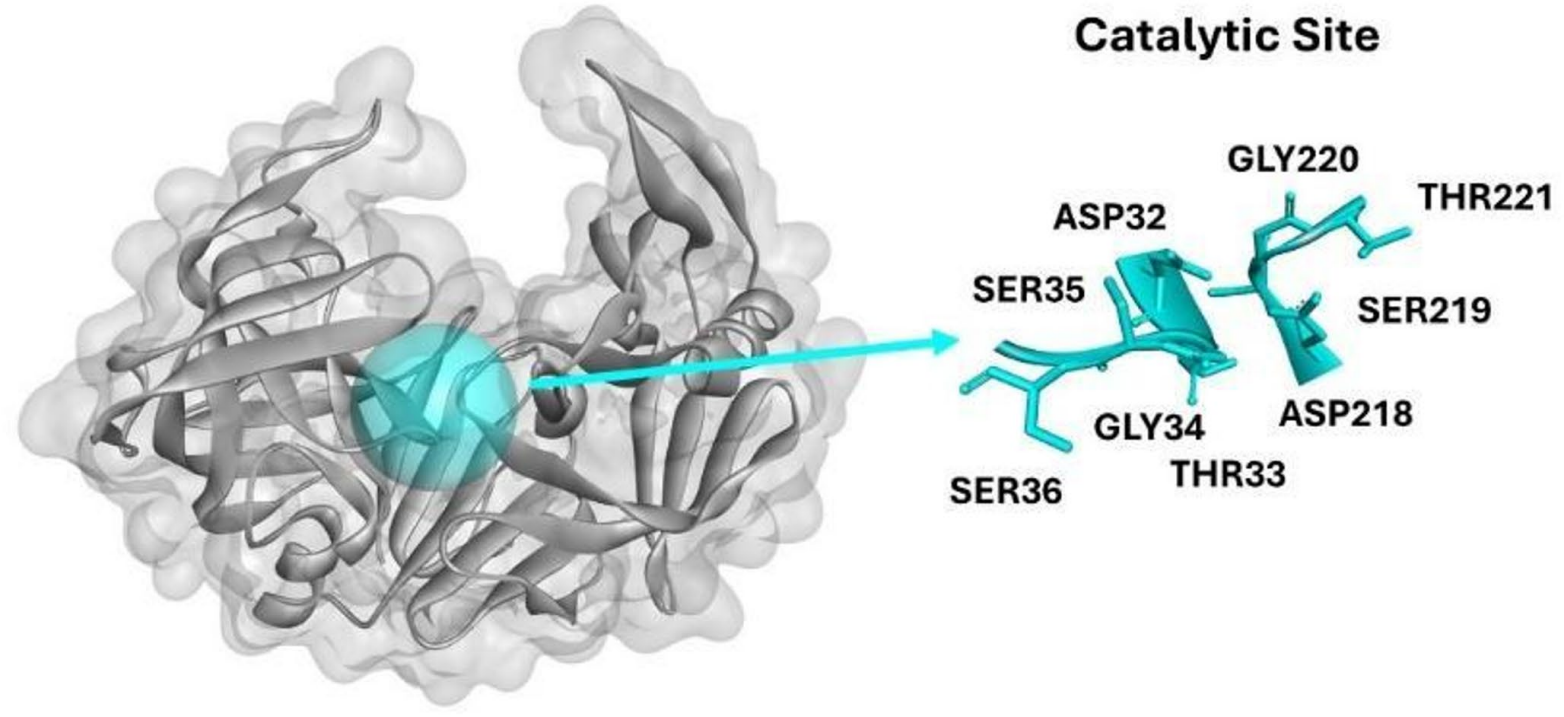
Structural representation of SAP2 from *C. albicans* highlighting the catalytic pocket (blue). The zoomed view delineates the key catalytic and adjacent residues (Asp32, Thr33, Gly34, Ser35, Ser36, Asp218, Ser219, Gly220, and Thr221) that constitute the functional core of the active site

The catalytic site of SAP2 from *C. albicans*, comprising residues 32–35 and 218–221, represents a crucial region for enzymatic activity (Fig. 2) and therefore an ideal target for the inhibitor identification (Cutfield et al. 1995). The catalytic region contains aspartic acid residues at positions 32 and 218, which are essential for the protein substrate hydrolysis (Pranav Kumar and Kulkarni 2002). The importance of this site is further supported by previous molecular docking analysis studies showing that effective SAP2 inhibitors interact directly with these catalytic residues, enabling peptide-based repurposing strategies aimed at inhibiting *C. albicans* through targeted engagement of this receptor (Calugi et al. 2012). Furthermore, the evolutionary conservation of this region among different *Candida* species underscores its essential role in enzymatic function (Bras et al. 2024). Effective SAP2 inhibitors typically interact directly with these catalytic residues, supporting peptide-repurposing strategies aimed at inhibiting *C. albicans (*Li et al. 2020). Targeting this site for docking AMPs is therefore a strategic approach, as it enables detailed investigation of ligand interactions within the catalytic cleft and provides a clear mechanistic basis for potential direct inhibition of SAP2 enzymatic activity (Pranav Kumar and Kulkarni 2002).

The structural integrity of the prepared SAP2 receptor was validated. Ramachandran plot analysis via PROCHECK demonstrated its stereochemical quality, with 89.7% of residues in the most favored regions and 9.3% in additional allowed regions. The residues found in generously allowed (Gln11 and Asn269) or disallowed regions (Asn160) are located in peripheral surface loops far from the catalytic pocket. Interaction mapping confirmed that none of these participate in any relevant receptor-ligand interactions, not compromising the docking grids or binding pose predictions. The overall structural reliability of the receptor was further corroborated by an ERRAT quality factor of 88.41, a VERIFY 3D score of 80.91%, and a highly reliable ProSA Z-score of − 7.63 (Fig. S5).

**Fig. 3:**
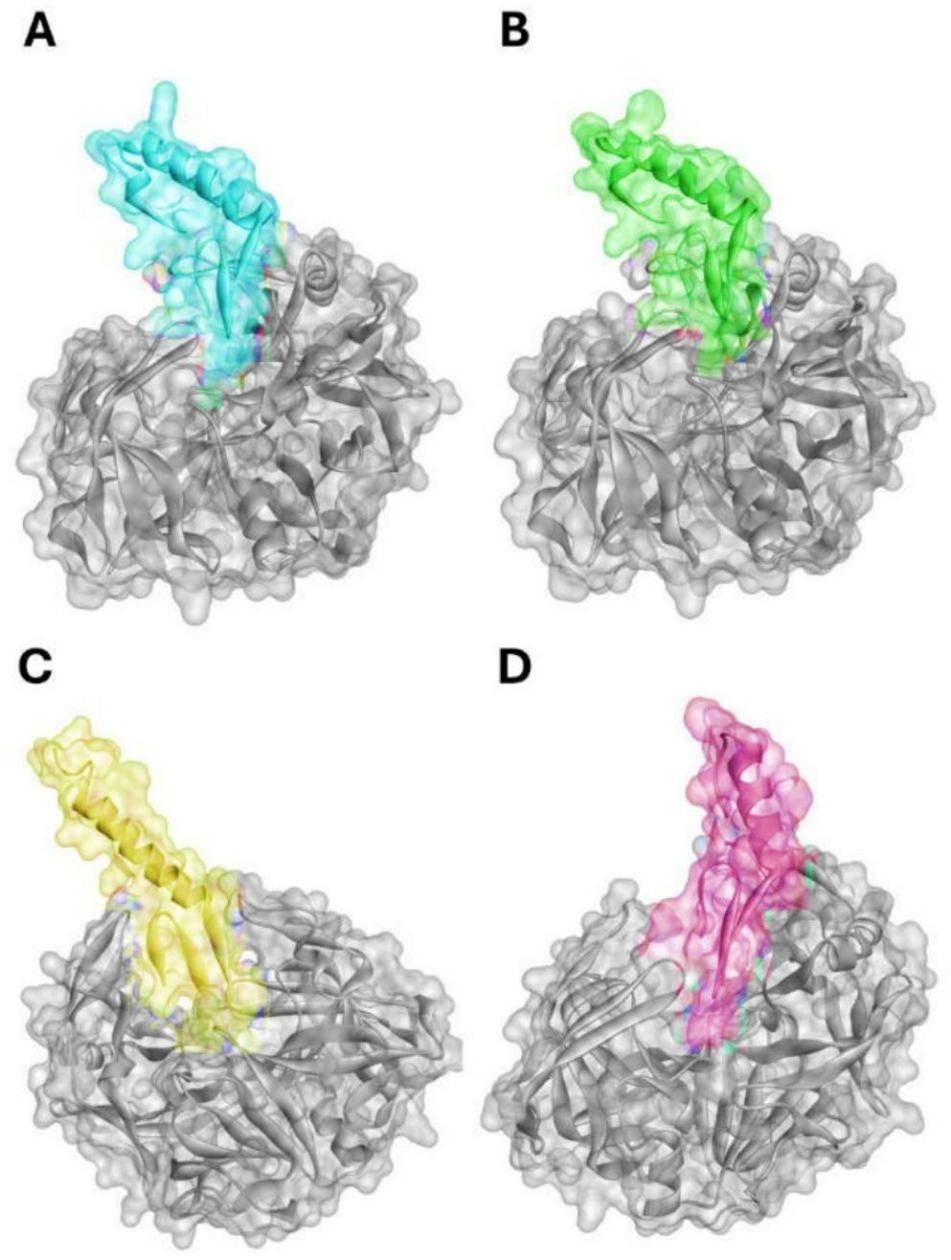
Three-dimensional structures of SAP2 docked with:** A** Coagulin A,** B** Pediocin PA-1,** C** Penocin A and;** D** Plantaricin 423, generated by AlphaFold v2. Protein surface representation was created in Discovery Studio. SAP2 is shown in grey, and ligands (peptides) are represented in distinct colors

Molecular docking analyses performed revealed distinct binding behavior among the four evaluated peptides toward SAP2. The three-dimensional structures of the enzyme–receptor complexes are presented in Fig. 3. Molecular docking results (Docking Score, Confidence Score, and Ligand RMSD (Å)) for the *C. albicans* SAP2 complexes with the baseline molecule Benzamidine and the pediocin-like bacteriocins Coagulin A, Pediocin PA-1, Penocin A, and Plantaricin 423 are presented in Table S1 of the Supplementary Material.

The functionality and accuracy of the proposed docking protocol were strongly validated by the redocking of the reference inhibitor benzamidine into the SAP2 active site, which yielded an exceptionally low ligand RMSD of 0.28 Å, successfully reproducing the native crystallographic pose. Comparatively, benzamidine exhibited a docking score of −  128.08 and a confidence score of 0.3921. Coagulin A exhibited the most favorable docking score (− 203.71 kcal.mol-1), followed by Pediocin PA-1 (− 184.91 kcal.mol-1), whereas Penocin A and Plantaricin 423 presented comparatively weaker scores (− 170.96 kcal.mol-1 and − 165.54 kcal.mol-1, respectively), but still demonstrated significant interaction potential. The elevated ligand RMSD values observed for the peptides compared to benzamidine are characteristic of their macromolecular nature and intrinsic flexibility, reflecting extensive conformational sampling during the docking process (Table S1).

Collectively, these results identify Coagulin A as the peptide with the strongest predicted binding affinity and highest docking confidence, while also demonstrating that all peptides engage a wide conformational landscape when interacting with SAP2.

Docking results specifically directed toward the SAP2 catalytic pocket indicated that all peptide–SAP2 complexes established interactions within these functionally critical regions. Detailed interaction mapping revealed that the reference inhibitor benzamidine is stabilized primarily by hydrogen bonds with the catalytic dyad ASP32 and ASP218, as well as GLY34, TYR84, ASP86, GLY220, and THR221, along with a hydrophobic interaction with Tyr225. Additionally, Coagulin A and Pediocin PA-1 engaged the critical catalytic pocket but established a much more extensive network of interactions. For instance, Coagulin A formed a strong electrostatic interaction with the catalytic residue ASP218, in addition to an extensive hydrogen-bonding network involving ASP86, TYR225, GLN228, ASN249, SER250, TYR252, VAL300, ASN301, and ASP302. Coagulin A was further anchored by a non-conventional contact with the backbone oxygen of Ser250. Notably, Pediocin PA-1 formed robust simultaneous hydrogen bonds and electrostatic interactions with both residues of the catalytic dyad (ASP32 and ASP218) through its LYS11 residue.

Furthermore, interaction profiling of Penocin A and Plantaricin 423 revealed that, despite comparatively weaker docking scores, both peptides successfully engaged the critical catalytic dyad. Specifically, Penocin A established strong electrostatic interactions between its lysine residues (LYS11 and LYS12) and the receptor’s ASP32 and ASP218, while Plantaricin 423 interacted with both ASP32 and ASP218 through its LYS11 residue. The sheer volume and quality of these non-covalent contacts perfectly explain their highly favorable docking scores compared to the small-molecule reference (Detailed interaction lists are provided in Table S2). Altogether, the docking outcomes highlight that these peptide–SAP2 complexes constitute promising candidates for continued mechanistic investigation, particularly given the established role of SAP2 as a major virulence determinant in *C. albicans* (Copping et al., 2005).

### MM/GBSA (molecular mechanics/generalized born surface area)

**Fig. 4:**
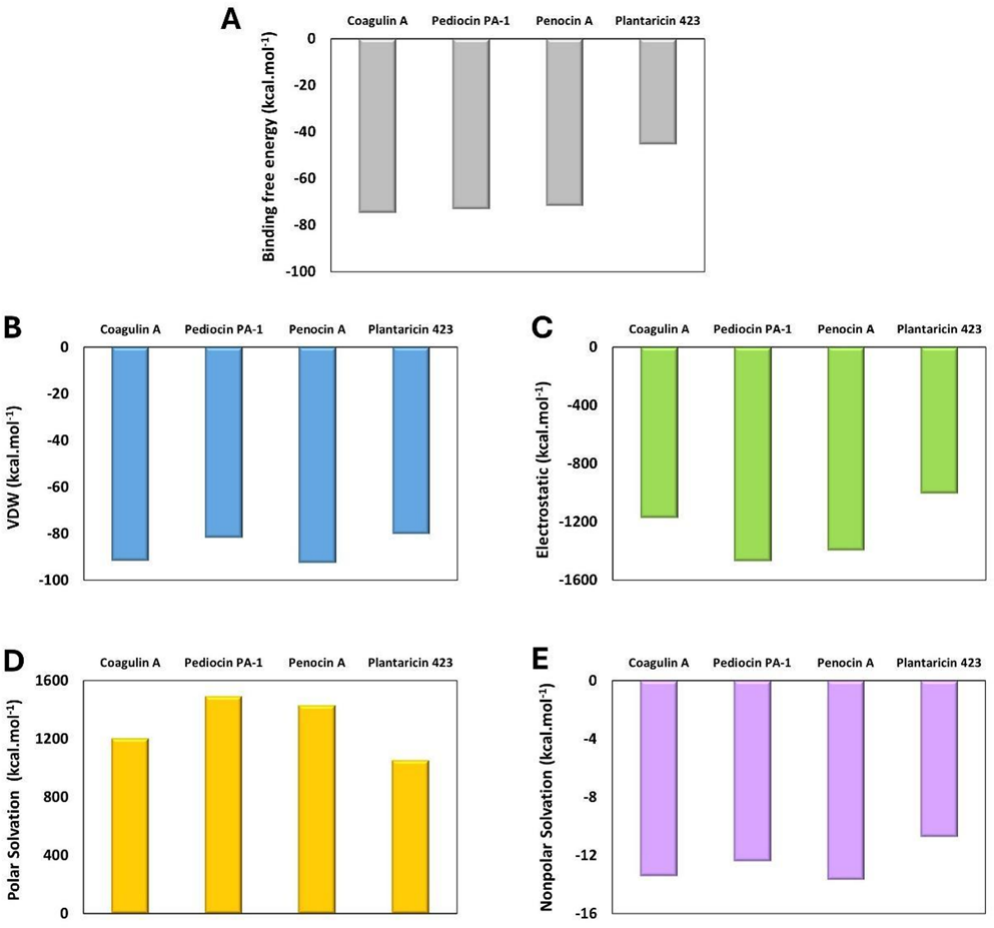
Binding free energy (kcal·mol^− 1^) of the peptide–SAP2 complexes:** A** total binding energy,** B** van der Waals,** C** electrostatic,** D** polar solvation, and** E** nonpolar solvation contributions for Coagulin A, Pediocin PA-1, Penocin A, and Plantaricin 423

MM/GBSA analysis of the complexes between SAP2 and all peptides—Pediocin PA-1, Coagulin A, Penocin A, and Plantaricin 423—was conducted as a static preliminary screening to dissect the initial energetic driving forces of these novel macromolecular inhibitors, revealing distinct interaction profiles, driven by differences in van der Waals, electrostatic, polar solvation, and nonpolar solvation contributions (Fig. 4). The binding energy values for the complexes are presented in Table S3 of the Supplementary Material. Although the peptides share broadly similar residues, the relative frequency and positional recurrence of LYS, TYR, TRP, GLY, CYS, SER, THR and HIS produced markedly divergent energetic contributions and structural engagement patterns within the receptor (SAP2) surface.

Regarding total binding energy, Coagulin A exhibited the highest binding affinity (− 74.69 kcal.mol^− 1^), followed by Pediocin PA-1 (− 73.08 kcal.mol^− 1^), Penocin A (− 71.7 kcal.mol^− 1^), and Plantaricin 423 (-45.32 kcal.mol^− 1^) (Fig. 4A). Evaluation of the individual energetic contributions revealed clear patterns distinguishing favorable and unfavorable interactions across the four peptides. In all complexes, van der Waals (VDW) energies were consistently favorable (Fig. 4B), reflecting stabilizing hydrophobic and steric complementarity, with Penocin A and Coagulin A showing the most negative VDW interactions (− 92.62 kcal.mol^− 1^ and − 91.65 kcal.mol^− 1^, respectively), consistent with deeper or more packed peptide interface. The electrostatic (ELE) interactions represented the most substantial favorable contribution for all peptides-SAP2 complexes, dominated by stabilization from their positively charged residues interacting with the negatively charged SAP2 surface (Fig. 4C). Pediocin PA-1 exhibited the strongest electrostatic contribution (− 1468.99 kcal.mol^− 1^), followed by Penocin A (− 1395.14 kcal.mol^− 1^), Coagulin A (− 1172.71 kcal.mol^− 1^), and Plantaricin 423 (− 1005.99 kcal.mol^− 1^), mirroring the relative abundance and accessibility of charged groups. This suggests that this ligand forms substantial charge interactions with the receptor residues, known to play a critical role in SAP inhibitor binding (Zhou and Pang 2018).

Notably, the polar energetic contributions (GB) were consistently unfavorable for all complexes (Fig. 4D and E). The GB values increase with the ELE values, such that peptides exhibiting stronger electrostatic interactions incurred higher GB values. Consequently, Pediocin PA-1 and Penocin A showed the largest unfavorable GB values (1490.10 and 1429.75 kcal·mol^− 1^, respectively), whereas Plantaricin 423, which engaged in weaker charge-mediated interactions, exhibited a substantially lower value (1051.68 kcal·mol^− 1^). The nonpolar solvation (SA) values contributed modestly in a favorable manner across all peptides, with comparable magnitudes for most, except for Plantaricin 423, whose weaker hydrophobic interactions resulted in the smallest SA contribution (− 10.76 kcal·mol^− 1^). The most favorable SA contribution was observed for Penocin A (− 13.70 kcal·mol^− 1^), in line with its stronger nonpolar contact (Fig. 4E). Overall, the total binding energy reflected a balance between favorable VDW, ELE, and SA contributions and the opposing GB interactions. Peptides enriched in positively charged and aromatic residues (such as LYS, TYR, and TRP) exhibited the strongest binding, as observed for Pediocin PA-1 and Penocin A. In contrast, the peptide with fewer charged and aromatic residues at the interface, Plantaricin 423, displayed weaker total interaction energies, reflecting a combination of reduced favorable electrostatic and nonpolar interactions and a relatively higher polar solvation. Coagulin A, with an intermediate composition of LYS and aromatic residues, showed stabilization comparable to Pediocin PA-1 and Penocin A, consistent with its total binding energy.

**Fig. 5:**
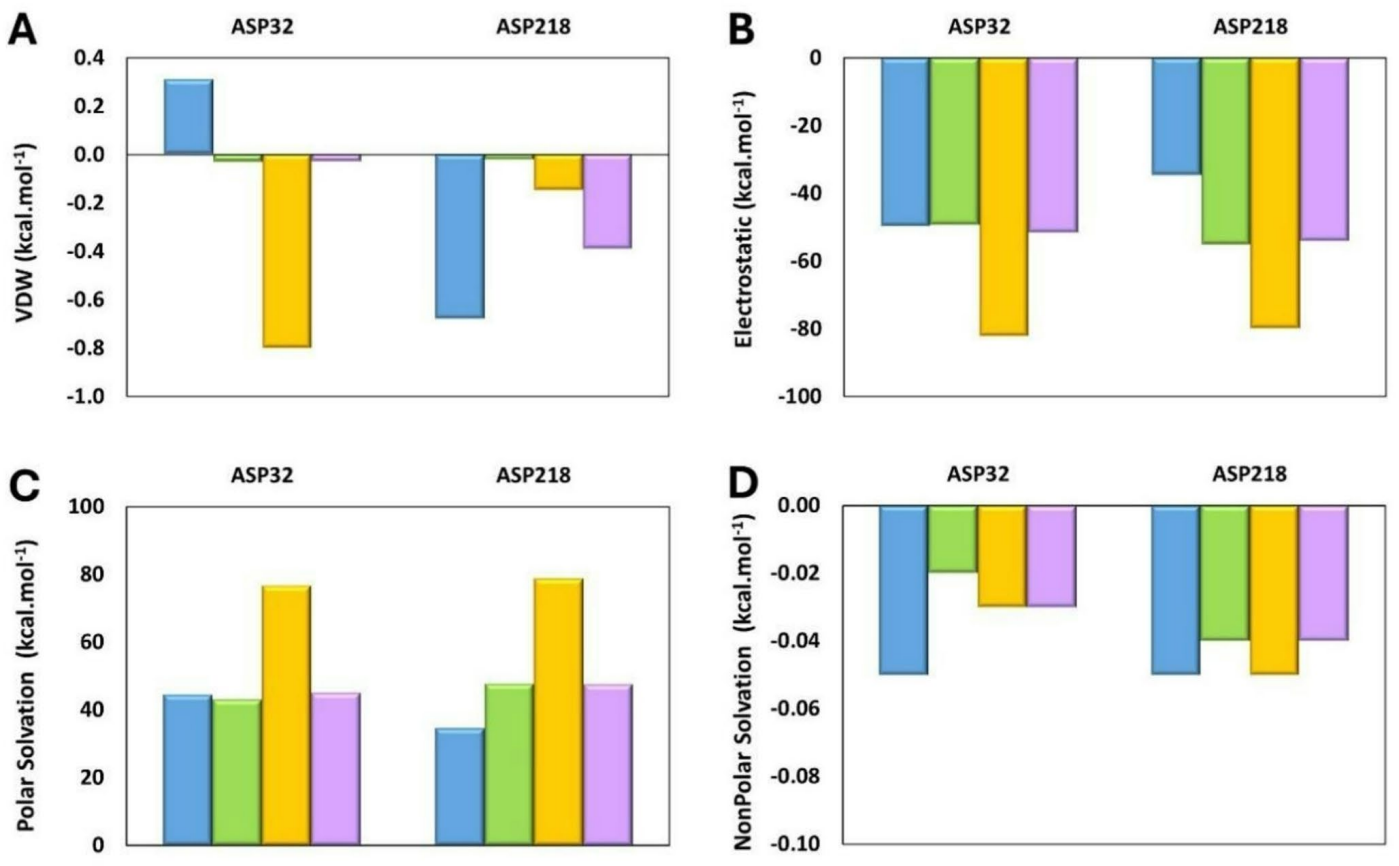
Binding free energy (kcal·mol^− 1^) for the catalytic dyad residues (ASP32 and ASP218) of SAP2 in complex with Coagulin A (blue), Pediocin PA-1 (green), Penocin A (yellow), and Plantaricin 423 (purple):** A** van der Waals,** B** electrostatic,** C** polar solvation, and** D** nonpolar solvation

The MM/GBSA assessment of the peptide at the SAP2 active site amino acid residues is depicted in Fig. 5. The complete list of SAP2 amino acid residue binding energies for all peptides is provided in Tables S4–S7 of the Supplementary Material. The ASP32 and ASP218 residues that compose the catalytic dyad define the central region of the SAP2 active site, where substrate binding and catalysis occur, and are required for the enzyme function (Pranav Kumar and Kulkarni 2002).

Analysis of binding energies revealed clear differences in how the peptides interact with the catalytic residues of SAP2. VDW interactions were uniformly small for all complexes and did not significantly distinguish the peptides, although Penocin A exhibited slightly less favorable values at ASP32, consistent with minor steric incompatibilities at this position. In contrast, ELE interactions were the major interaction of binding in the peptide-SAP2 complexes. Penocin A displayed markedly stronger electrostatic attraction toward both ASP32 and ASP218 than the other peptides, indicating a superior capacity to stabilize the negatively charged dyad. Pediocin PA-1 and Plantaricin 423 showed intermediate electrostatic contributions, whereas Coagulin A exhibited the weakest interaction. The GB interactions, which represent the polar solvation contribution, followed the overall ELE trend. However, all GB values were positive, meaning that polar solvation was uniformly unfavorable across all peptides. In this context, a higher GB value corresponds to a more unfavorable contribution. The SA values, corresponding to nonpolar solvation, remained minimal and highly similar across peptides, suggesting that they do not significantly influence peptides interactions with the SAP2 catalytic dyad. Interactions between ligands and these residues are therefore mechanistically critical, as perturbation of this microenvironment can effectively compromise catalytic behavior of SAP2 and lead to inhibition (Borelli et al. 2008). In this context, selecting molecules that bind the catalytic dyad with appropriate energetic and structural complementarity represents a rational approach for inhibitor design and contributes to the development of targeted strategies against *C. albicans* (Silva et al. 2019).

While this static MM/GBSA analysis provided a valuable preliminary screening, the macromolecular and intrinsically flexible nature of pediocin-like bacteriocins demanded a dynamic energetic evaluation. Therefore, these initial static estimates were subsequently challenged and validated through trajectory-based binding energy calculations during molecular dynamics.

### Molecular dynamics

To further assess the stability and dynamic behavior of the peptide–SAP2 complexes following molecular docking and MM/GBSA analysis, all complexes were subjected to molecular dynamics (MD) simulations. Whereas the reference inhibitor was used as reference in the docking phase, it was excluded from molecular dynamics simulations due to its distinct nature, as a rigid small molecule (~ 120 Da) whose binding stability is already experimentally validated by the co-crystallized complex (PDB ID: 3PVK). MD simulations and energetic evaluations were prioritized for the peptide-SAP2 complexes to assess whether these novel, large macromolecules could overcome their intrinsic flexibility to achieve a stable, locked conformation within the active site.

**Fig. 6:**
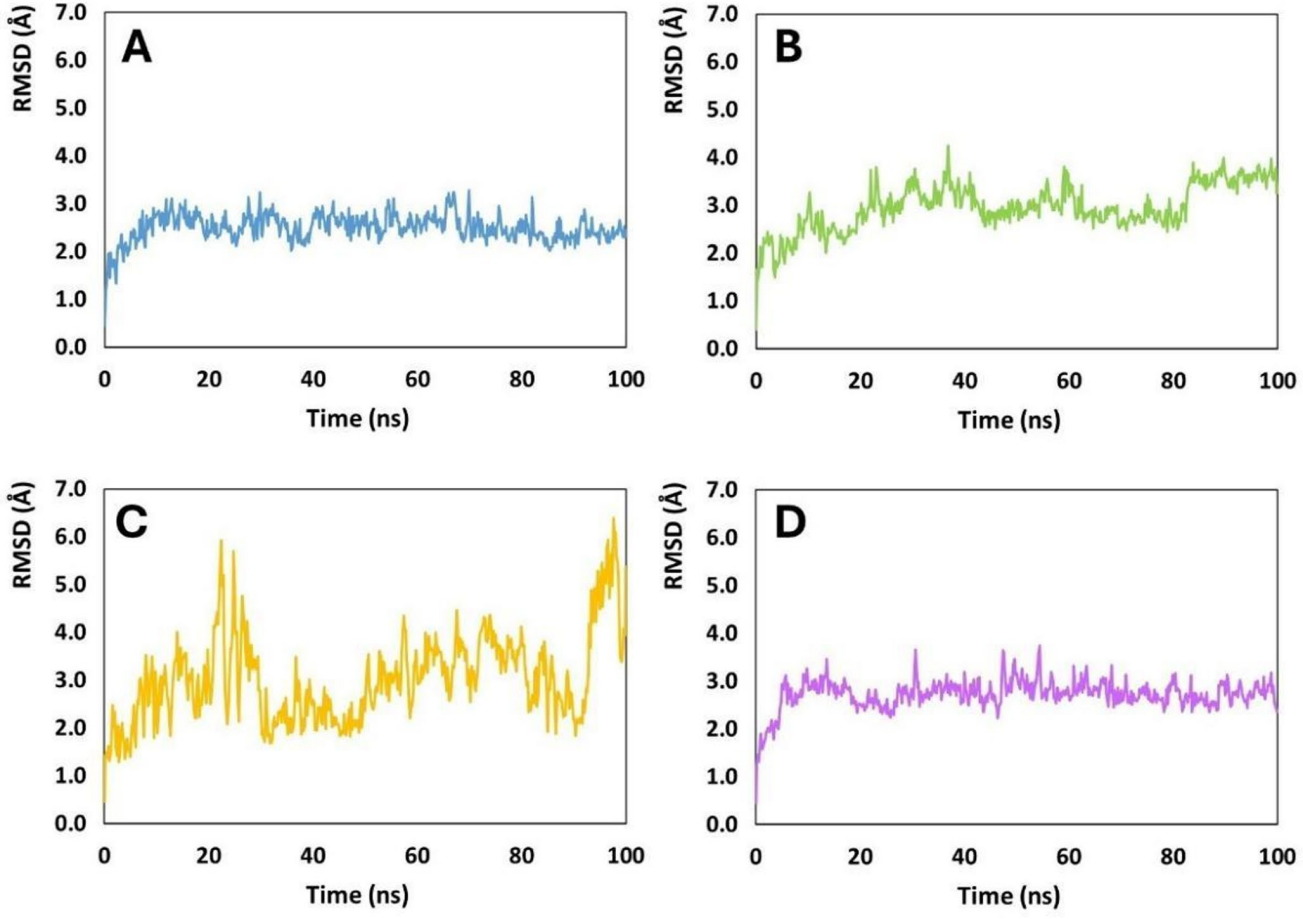
Root-mean-square deviation (RMSD) of the SAP2–peptide complexes with:** A** Coagulin A,** B** Pediocin PA-1,** C** Penocin A, and** D** Plantaricin 423

RMSD profiles revealed distinct stability regimes among the four SAP2–peptide complexes (Fig. 6). Pediocin PA-1 exhibited the most restrained fluctuations, maintaining RMSD values predominantly between 1.5 and 3.0 Å throughout the trajectory. Coagulin A showed a similar magnitude of fluctuation, although with slightly more pronounced variability and a broader upper range approaching ~ 3.0 Å. In contrast, Penocin A and Plantaricin 423 displayed substantially higher RMSD amplitudes, frequently exceeding 3.5 Å and in several segments surpassing 4.0 Å, indicating enhanced structural mobility relative to Pediocin PA-1 and Coagulin A.

All complexes underwent an initial equilibration phase within the first 1–3 ns, during which RMSD values stabilised from early conformational relaxation. Pediocin PA-1 and Coagulin A reached a relatively stable regime shortly thereafter, oscillating within a narrow window with no sustained drift, consistent with the formation of stable intermolecular contacts. Penocin A and Plantaricin 423, however, entered a pattern of progressive RMSD escalation beginning around 8–12 ns, followed by recurrent peaks above 4 Å from approximately 20 ns onwards. These high-amplitude fluctuations persisted intermittently across the remainder of the trajectory, indicating recurrent structural rearrangements or sampling of multiple conformational basins. Across the final third of the simulation (60–100 ns), Pediocin PA-1 and Coagulin A maintained RMSD values in a confined range (generally 2.3–3.1 Å), whereas Penocin A and Plantaricin 423 exhibited sustained high-mobility states with RMSD values frequently between 3.5 and 5.0 Å. The largest RMSD excursions were observed for Penocin A, which reached values above 6.0 Å in the late simulation, followed by Plantaricin 423. Collectively, the RMSD trajectories identify Pediocin PA-1 and Coagulin A as forming the most conformationally stable complexes with SAP2, while Penocin A and Plantaricin 423 induce markedly greater structural mobility and undergo extensive conformational exploration throughout the simulation.

**Fig. 7:**
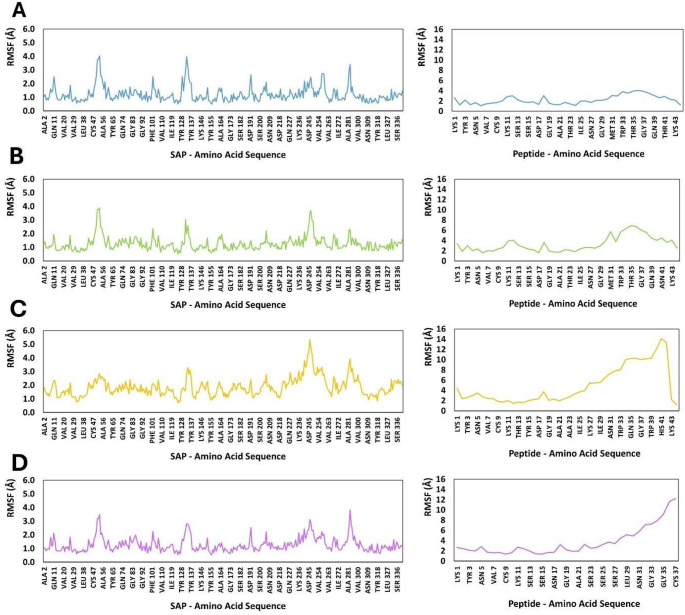
Root mean square fluctuation (RMSF) of the SAP2–peptide complexes with:** A** Coagulin A,** B** Pediocin PA-1,** C** Penocin A, and** D** Plantaricin 423

RMSF analysis revealed that SAP2 exhibits distinct flexibility profiles depending on the bound peptide (Fig. 7). Across the N-terminal region (residues 2–40), Pediocin PA-1 and Coagulin A maintained consistently low fluctuations, generally below ~ 1.3 Å, whereas Penocin A induced systematically higher mobility, with values in several positions exceeding 1.4–1.8 Å. Plantaricin 423 produced intermediate fluctuations, slightly above those observed for Pediocin PA-1 and Coagulin A but notably below Penocin A. This pattern indicates that Pediocin PA-1 and Coagulin A stabilize early structural segments of SAP2 more effectively than Penocin A, while Plantaricin 423 exerts moderate destabilizing influence.

In the central domain (residues 41–120), which encompasses catalytic and substrate-binding elements, all complexes showed a marked increase in mobility from residues 48–60 and again from 70 to 90. Nonetheless, the amplitude of these peaks depended strongly on the ligand. Pediocin PA-1 and Coagulin A maintained RMSF values largely within 1.0–2.5 Å, whereas Penocin A and Plantaricin 423 frequently drove fluctuations above 2.5 Å. The catalytic ASP32 remained highly stable across all complexes (< 1.0 Å), yet surrounding loop regions (e.g., 48–55, 72–84) were markedly more mobile in the Penocin A-SAP2 complex. This suggests that Penocin A perturbs local loop dynamics surrounding the active site to a greater extent than the other peptides.

The C-terminal region (residues 120–200) retained moderate flexibility for all complexes, but again Penocin A produced the highest amplitude fluctuations, frequently between 1.7 and 2.4 Å, with occasional peaks above 3.0 Å. Plantaricin 423 elicited similar but slightly less pronounced increases. In contrast, Pediocin PA-1 and Coagulin A consistently stabilized this region, maintaining RMSF values mostly below 1.6 Å.

The most pronounced mobility differences occurred in the distal C-terminal region (residues 200–260) and the extended loop cluster (residues 240–260). Across these positions, Penocin A and Plantaricin 423 induced large-scale fluctuations, often between 3.0 and 5.3 Å, with the highest values observed at residues 246–247 and 253–257. Coagulin A produced moderate increases, while Pediocin PA-1 yielded the lowest fluctuation amplitudes, remaining near or below 3.0 Å at all positions. These segments form a flexible external loop network, and the differences among ligands demonstrate distinct capacities to dampen or amplify global receptor mobility.

Finally, the extreme C-terminal region (residues 260–341) followed the same hierarchical trend. Penocin A consistently generated the highest RMSF, frequently between 2.0 and 4.0 Å, and Plantaricin 423 produced similarly elevated values at several positions. Coagulin A showed intermediate fluctuations, whereas Pediocin PA-1 again conferred the greatest stability, maintaining values predominantly below ~ 1.6 Å. Residues 292–298 and 330–340 were particularly sensitive to the identity of the bound peptide. Additionally, residues in the C-terminal region of the receptor contribute to van der Waals and hydrophobic interactions that are critical for inhibitor selectivity and binding and are particularly important for the overall efficacy of the complexes (Du et al. 2016); (Pranav Kumar and Kulkarni 2002).

Overall, the RMSF data demonstrate a consistent stability hierarchy across the SAP2: Pediocin PA-1 > Coagulin A > Plantaricin 423 > Penocin A. Pediocin PA-1 most effectively damps receptor flexibility across all structural domains, while Penocin A induces widespread enhancement of residue-level mobility, particularly within loop regions distal to the catalytic core. The dynamic behavior observed in these complexes may facilitate interactions with flexible inhibitors or those that require conformational adaptation of the active site (Huang et al. 2016), whereas more stable complexes are likely to promote rigid binding, potentially enhancing selectivity toward inhibitors with well-defined structures.

**Fig. 8:**
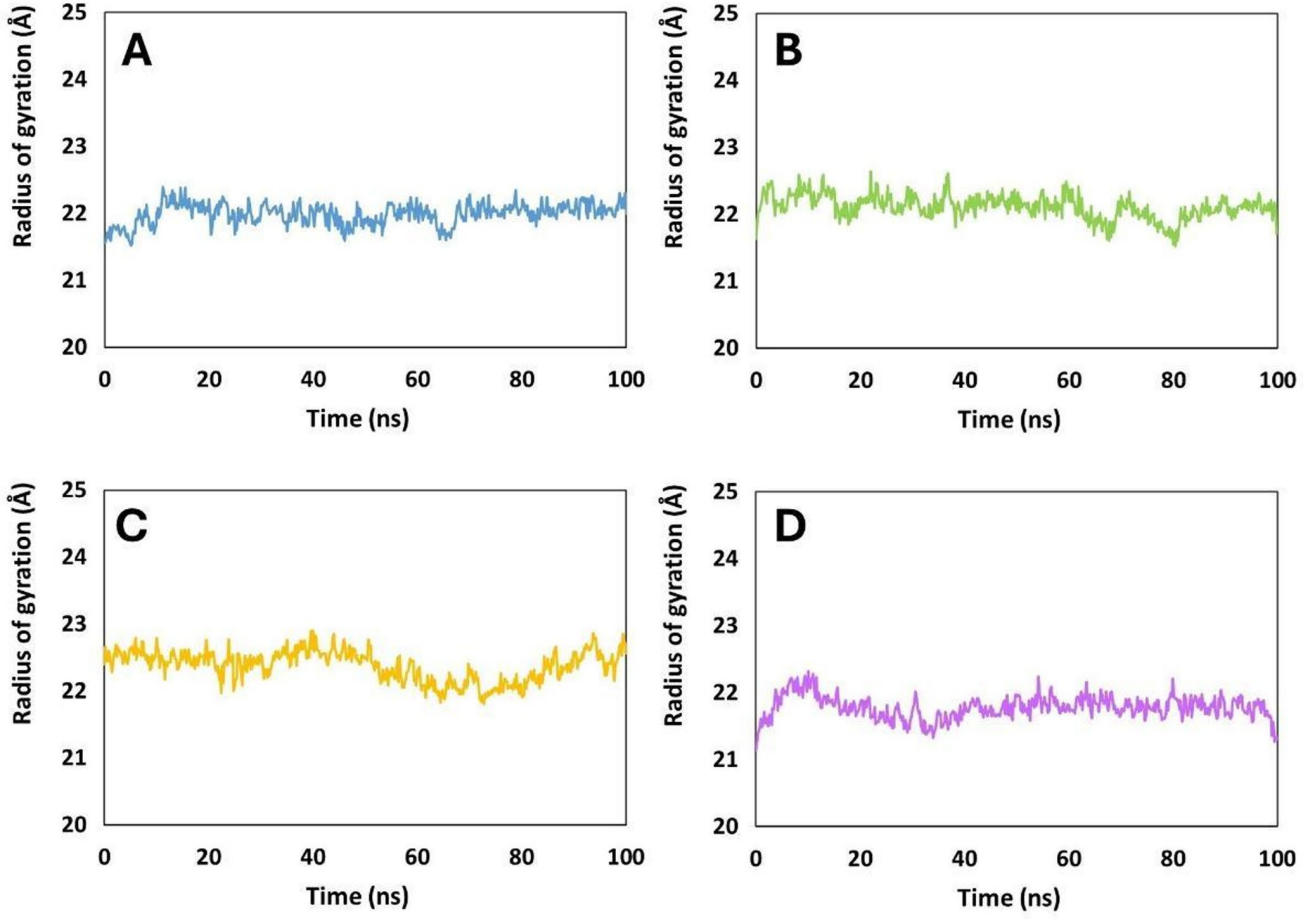
Radius of gyration (Rg) of the SAP2–peptide complexes with:** A** Coagulin A,** B** Pediocin PA-1,** C** Penocin A, and** D** Plantaricin 423

Across the MD trajectories, the four SAP2–peptide complexes showed clear differences in peptide compaction as reflected in their average radius of gyration (Rg) (Fig. 8). Pediocin PA-1 and Coagulin A exhibited the lowest mean Rg values (≈ 22.15 Å and ≈ 21.95 Å, respectively), indicating comparatively compact conformations. Plantaricin 423 maintained an intermediate mean Rg (≈ 21.85 Å), whereas Penocin A was consistently more expanded, displaying the highest average value (≈ 22.50 Å). Considered together, these data indicate two distinct behavioral classes: Pediocin PA-1 and Coagulin A, which achieve stable and compact conformations upon binding to SAP2, and Penocin A and Plantaricin 423, which retain more extended configurations. The mean values therefore provide an initial ranking of structural stabilization induced by SAP2.

Coagulin A exhibited a relatively restricted radius of gyration (Rg) profile (Fig. 8A), typically ranging from 21.7 to 22.1 Å, with transient deviations of slightly greater magnitude than those observed for Pediocin PA-1 (Fig. 8B). These fluctuations remained limited in both duration and amplitude, indicating that the Coagulin A complex attained a stable structural regime, albeit with modestly higher flexibility, consistent with compact and energetically favorable conformations typically associated with stable protein–ligand complexes (Lobanov et al. 2008). Pediocin PA-1 presented an equally narrow Rg distribution throughout the simulation, generally fluctuating within 21.8–22.3 Å. Deviations from this interval were infrequent and of low magnitude, suggesting that Pediocin PA-1 adopts a well-defined conformation in the SAP2-bound state and undergoes only minor global rearrangements, further supporting the presence of strong and persistent intermolecular contacts contributing to complex stability (Lobanov et al. 2008). Penocin A, in contrast, displayed a consistently expanded Rg profile, with values predominantly between 22.4 and 22.8 Å and frequent excursions above 22.9 Å (Fig. 8C). The absence of convergence toward a more compact conformation suggests that Penocin A retains substantial conformational mobility even after establishing interactions with SAP2, a behavior characteristic of peptides with higher intrinsic flexibility and localized motion (Pranav Kumar and Kulkarni 2002). Plantaricin 423 demonstrated intermediate behavior (Fig. 8D). During the early stages of the simulation, its Rg values ranged between 21.6 and 21.9 Å, but subsequently shifted upward, stabilizing around 21.9–22.1 Å. Although more constrained than Penocin A, Plantaricin 423 did not fully settle into a uniformly compact state and maintained modest but persistent variability over time, a controlled flexibility that may favor induced-fit mechanisms in dynamic binding environments (Mamonova et al. 2013); (Kamal et al. 2012).

The Rg analyses indicate the following order of conformational stabilization in the SAP2–peptide complexes: Pediocin PA-1 > Coagulin A > Plantaricin 423 > Penocin A. Pediocin PA-1 forms the most compact and least variable structure, followed closely by Coagulin A. Plantaricin 423 achieves only partial compaction, whereas Penocin A remains the most structurally dynamic and least stabilized upon binding to SAP2.

**Fig. 9:**
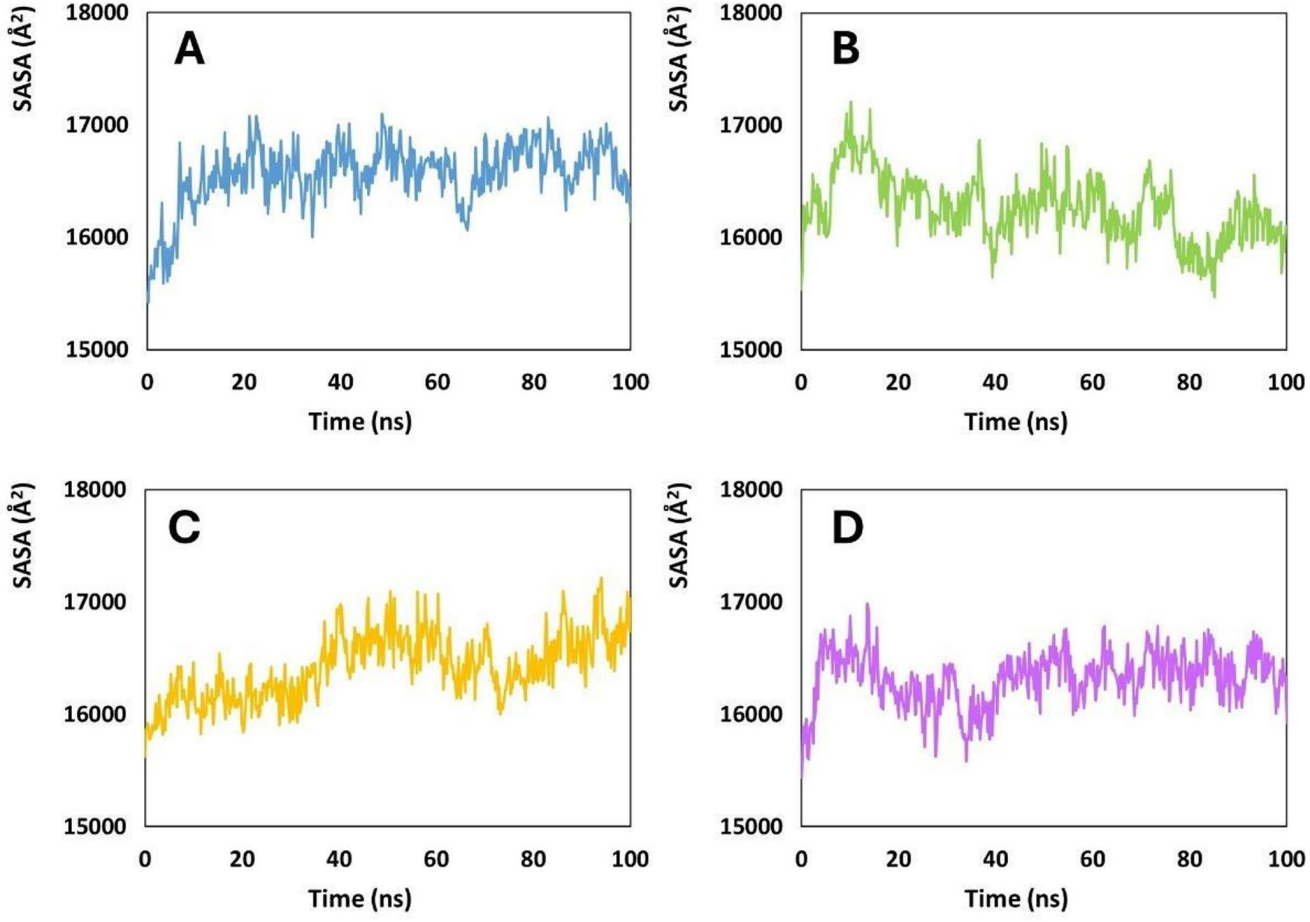
Solvent-accessible surface area (SASA) of the SAP2–peptide complexes with:** A** Coagulin A,** B** Pediocin PA-1,** C** Penocin A, and** D** Plantaricin 423

The solvent-accessible surface area (SASA) profiles of the SAP2–peptide complexes (Fig. 9) revealed clear differences in how each ligand modulated the global exposure of the SAP2 to solvent. Such analysis of solvent accessibility is critical for understanding protein–ligand interactions and the dynamic stability of complexes (Osuna et al. 2015; Du et al. 2016). When averaged over the full trajectories, Coagulin A (Fig. 9A) and Pediocin PA-1 (Fig. 9B) consistently drove SAP2 toward the lowest SASA values, both stabilizing around the mid-16,000 Å² range with relatively narrow dispersion. Plantaricin 423 (Fig. 9D) produced slightly higher values overall, while Penocin A (Fig. 9C) generated the most solvent-exposed states, frequently approaching or exceeding 17,000 Å². This distinct pattern in mean SASA values (Coagulin A ≈ Pediocin PA-1 < Plantaricin 423 < Penocin A) was maintained throughout the simulation and reflects distinct peptide-induced effects on SAP2 conformational fold. Such stability in solvent accessibility is directly related to the dynamic behavior of the SAP2 active sites and is critical for the efficacy of receptor–ligand complexes, particularly in the context of enzyme inhibition. The observed variations in accessibility suggest that while the complexes maintain overall stability, controlled flexibility allows for adaptive interactions, highlighting a balance between rigidity and dynamic behavior that may facilitate sustained inhibitor binding (Vishwanath and Srinivasan 2014).

Although Pediocin PA-1 and Coagulin A belonged to the same general regime of reduced solvent exposure, their dynamic signatures were not identical. Pediocin PA-1 maintained an especially constrained SASA distribution, characterized by smooth, low-amplitude oscillations with only brief excursions. The maintenance of consistent residue accessibility over time suggests that critical active site residues remain available for interaction with inhibitor ligands, thereby increasing the likelihood of effective and sustained binding, which is relevant for the modulation of pathogenicity (Martelli et al. 2016); (Savojardo et al. 2020).

The tight clustering of Pediocin PA-1-associated SASA frames suggests that its binding promotes relatively cohesive and compact conformational states of SAP2. Coagulin A exhibited slightly broader fluctuations, including intermittent expansions that momentarily raised the surface area, yet these episodes resolved quickly and did not deviate from the overall trend of moderate compaction. The presence of some variability in accessibility allows residues to adjust their conformations to accommodate different ligands, a key factor in the design of competitive inhibitors (Durrant and McCammon 2011).

In contrast, Penocin A produced a markedly different profile. SAP2–Penocin A complex repeatedly accessed expansive conformations, showing both the highest SASA maxima and the most frequent high-amplitude transitions. Periods of sustained elevation above 16,900 Å² emerged throughout the trajectory, implying a persistent increase in solvent exposure driven by Penocin A binding. Such behavior typically reflects enhanced mobility of peripheral loops or localized structural relaxation, indicating that Penocin A perturbs the protease scaffold more substantially than the other peptides. Plantaricin 423 occupied an intermediate zone between these extremes: although its complexes exhibited SASA values higher than those observed for Pediocin PA-1 and Coagulin A, the pronounced expansions characteristic of Penocin A were less common and generally short-lived. SAP2 therefore remained more compact in the presence of Plantaricin 423 than in the presence of Penocin A, but less so than with the two peptides that most effectively restricted solvent exposure. Based on SASA, which acts as an indicator of structural compactness and thermodynamic stability, where lower and more tightly distributed values signify more compact and energetically favorable structures, the relative stabilizing capacity of the peptides follows the order Pediocin PA-1 ≈ Coagulin A > Plantaricin 423 > Penocin A. The first two peptides promote the most cohesive SAP2 conformations, Plantaricin 423 allows moderate expansion without sustained destabilization, and Penocin A induces the highest and most persistent solvent exposure, marking it as the least favorable ligand in terms of maintaining compact SAP2 states.

**Fig. 10:**
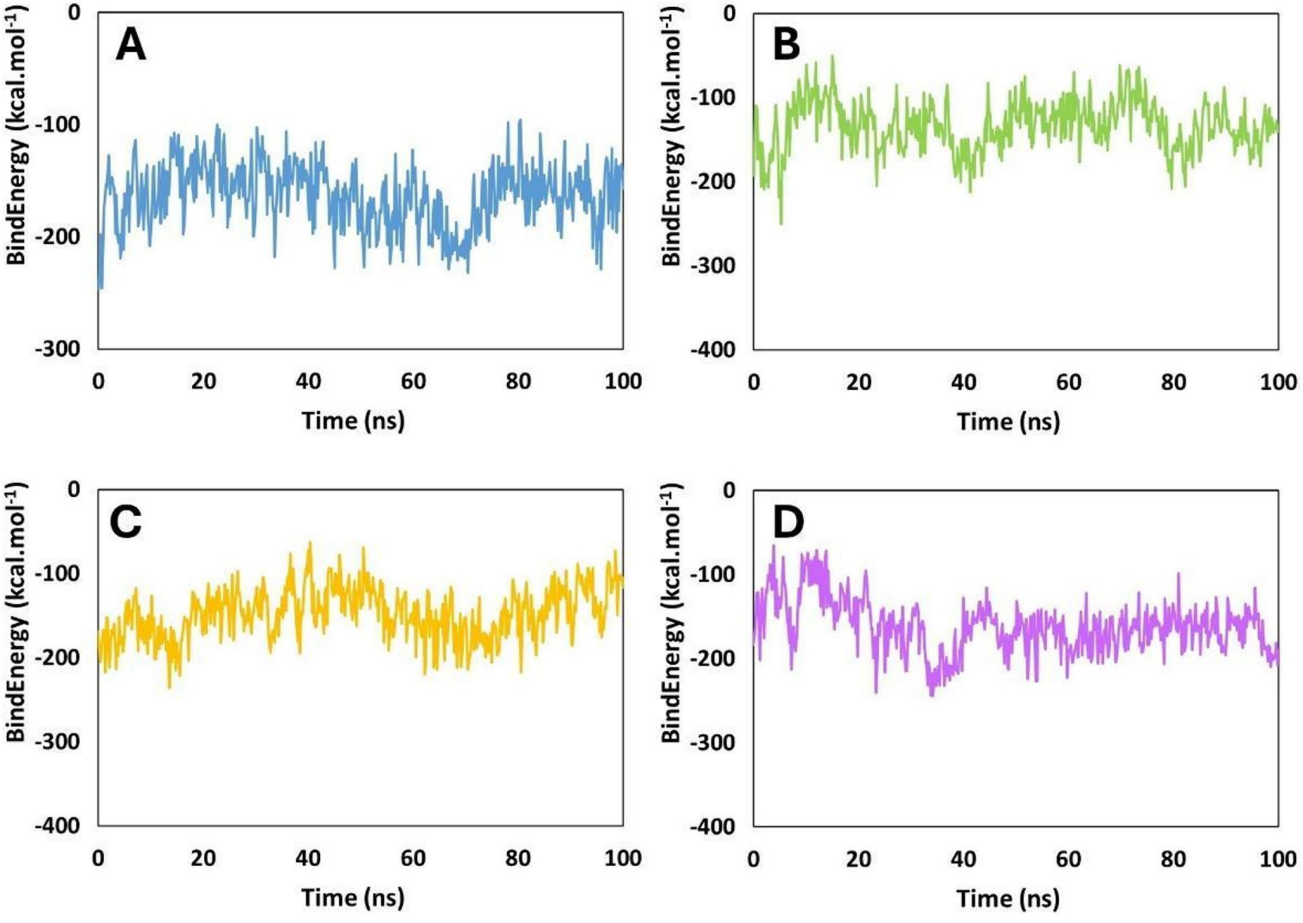
Binding energy (kcal.mol^− 1^) of the SAP2–peptide complexes with:** A** Coagulin A,** B** Pediocin PA-1,** C** Penocin A, and** D** Plantaricin 423

The binding energy of the SAP2–peptide complexes revealed distinct stability patterns among the four peptides (Fig. 10). Coagulin A maintained the most consistently favorable interactions (Fig. 10B). The binding energies fluctuated mostly between − 100 and − 250 kcal.mol^− 1^ across the 100 ns simulation, showing persistent stability with only minor deviations. This indicates a strong and relatively stable association with SAP2 throughout the trajectory. Pediocin PA-1 (Fig. 10A) displayed intermediate binding stability. Its energies ranged roughly from − 50 to − 250 kcal.mol^− 1^, with frequent fluctuations in the early simulation stages, followed by a moderate stabilization pattern in the middle to late stages. Although it occasionally reached highly favorable states (− 200 kcal.mol^− 1^ or lower), these events were intermittent and less persistent than in Coagulin A. Penocin A (Fig. 10C) exhibited greater energetic variability. The binding energies varied widely from approximately − 60 to − 220 kcal.mol^− 1^, with many short-lived minima and frequent transitions toward less favorable values. This indicates a less stable and more dynamic binding mode with SAP2 compared to Coagulin A and Pediocin PA-1. Plantaricin 423 (Fig. 10D) showed the most heterogeneous and least favorable binding profile. Its energies ranged from − 60 to − 250 kcal.mol^− 1^, with repeated oscillations toward higher (less negative) values. Extended periods of relative destabilization were observed, highlighting a weak and highly dynamic interaction with SAP2.

The binding energy results establish a clear hierarchy of interface stability: Coagulin A > Pediocin PA-1 > Penocin A > Plantaricin 423. The apparent divergence between these energetic affinities and global structural metrics (where Plantaricin 423 exhibited lower RMSD and Rg fluctuations than Penocin A) arises because MM/GBSA quantifies the localized strength of the peptide-receptor interface. In contrast, metrics like RMSD, Rg, and SASA evaluate the conformational dynamics of the entire system in solvent. Because these bacteriocins are larger molecules, Penocin A establishes local interactions at the active site while the remainder of its long chain remains highly flexible and solvent-exposed, leading to higher RMSD and Rg values. Conversely, Plantaricin 423 binds with weaker affinity, adopting a more constrained conformation. Thus, local binding and global structural flexibility confirm Coagulin A and Pediocin PA-1 as the most optimized and robust ligands across all parameters.

## Conclusions

This study provides a comprehensive computational evaluation of pediocin-like bacteriocins as potential inhibitors of *C. albicans* SAP2, a key virulence factor in this pathogenic fungus. By integrating molecular docking, MM/GBSA decomposition, and molecular dynamics simulations, we established a coherent and hierarchically consistent ranking of ligand compatibility with the SAP2 catalytic site. Across all computational steps, Coagulin A and Pediocin PA-1 emerged as the most favorable ligands. Molecular Docking analyses revealed that Coagulin A achieved the most favorable score and highest confidence, closely followed by Pediocin PA-1, while Penocin A and Plantaricin 423 exhibited markedly weaker docking scores and lower confidence metrics. These findings were reinforced by MM/GBSA analyses, which provided residue-specific energetic insights: Coagulin A and Pediocin PA-1 displayed balanced van der Waals and electrostatic contributions at the catalytic dyad (ASP32 and ASP218), consistent with stable and productive engagement. In contrast, Penocin A showed large but energetically offset electrostatic terms, and Plantaricin 423 exhibited intermediate interaction profiles, less optimized than those of Coagulin A or Pediocin PA-1. Molecular dynamics simulations further validated these observations, confirming the structural stability of the peptide-SAP2 complexes. RMSD and RMSF results indicated that Coagulin A and Pediocin PA-1 induced tighter anchoring of residues and enhanced local stability, whereas Penocin A increased flexibility across the catalytic region. Radius of gyration and solvent-accessible surface area analyses corroborated these results, showing that Coagulin A and Pediocin PA-1 promoted the most compact and least solvent-exposed SAP2 conformations, Plantaricin 423 displayed intermediate stabilization, and Penocin A led to expanded, highly solvated states. Overall, the convergence of all results identifies Coagulin A and Pediocin PA-1 as the most promising SAP2 inhibitors, with Plantaricin 423 as a moderate candidate and Penocin A as the least favorable. This work establishes a robust computational framework for the rational design of novel antifungal therapeutics, supporting the potential of pediocin-like bacteriocins to contribute to the development of targeted and effective treatments against resistant *C. albicans*.

## Supplementary Information

Below is the link to the electronic supplementary material.

Supplementary Material 1

## Data Availability

No datasets were generated or analysed during the current study.
