# Supplementary material for: Multistep molecular assessment of pediocin-like bacteriocins as antifungal agents targeting secreted aspartic protease 2 (SAP2) of Candida albicans through computational modeling and molecular dynamics
Source: In Silico Pharmacol. 2026 May 26;14(2):146. doi: 10.1007/s40203-026-00642-3 (PMC13212834; doi:10.1007/s40203-026-00642-3)
Supplement: Supplementary file 1 — Supplementary Material 1 [file 40203_2026_642_MOESM1_ESM.docx]

**Multistep molecular assessment of pediocin-like bacteriocins as antifungal agents targeting secreted aspartic protease 2 (SAP2) of *Candida albicans* through computational modeling and molecular dynamics**

Iago Rodrigues Blanco^1,2^, Ricardo Pinheiro de Souza Oliveira^1^, Matheus M. Pereira^2*^

^1^Department of Biochemical and Pharmaceutical Technology, School of Pharmaceutical Sciences, University of São Paulo, São Paulo, Brazil.

^2^ University of Coimbra, CERES, Department of Chemical Engineering, Rua Sílvio Lima, Pólo II – Pinhal de Marrocos, 3030-790 Coimbra, Portugal.

***Corresponding Author:** matheus@eq.uc.pt

**
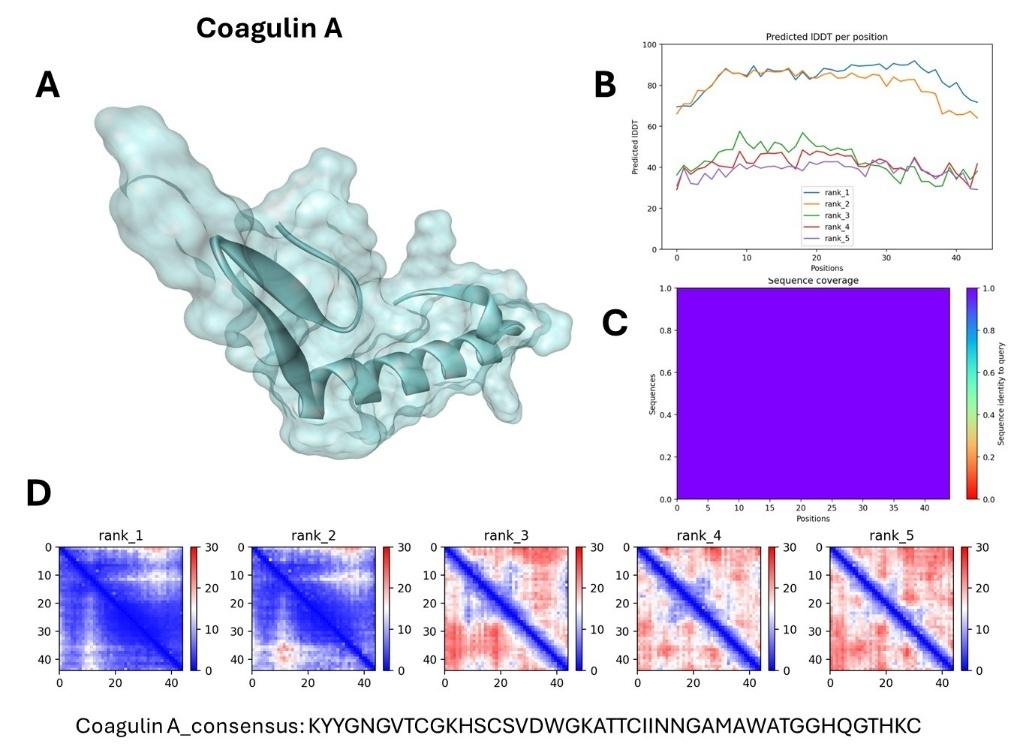
**

**Figure S1-** AlphaFold2 prediction and structural evaluation of the Coagulin A: (A) Three-dimensional structural model of Coagulin A predicted by AlphaFold2, (B) pLDDT scores for the five best-scoring models (rank_1–rank_5), (C) Sequence coverage plot showing the multiple sequence alignment depth used during structure prediction and (D) PAE heatmaps for the five ranked models of Coagulin A.


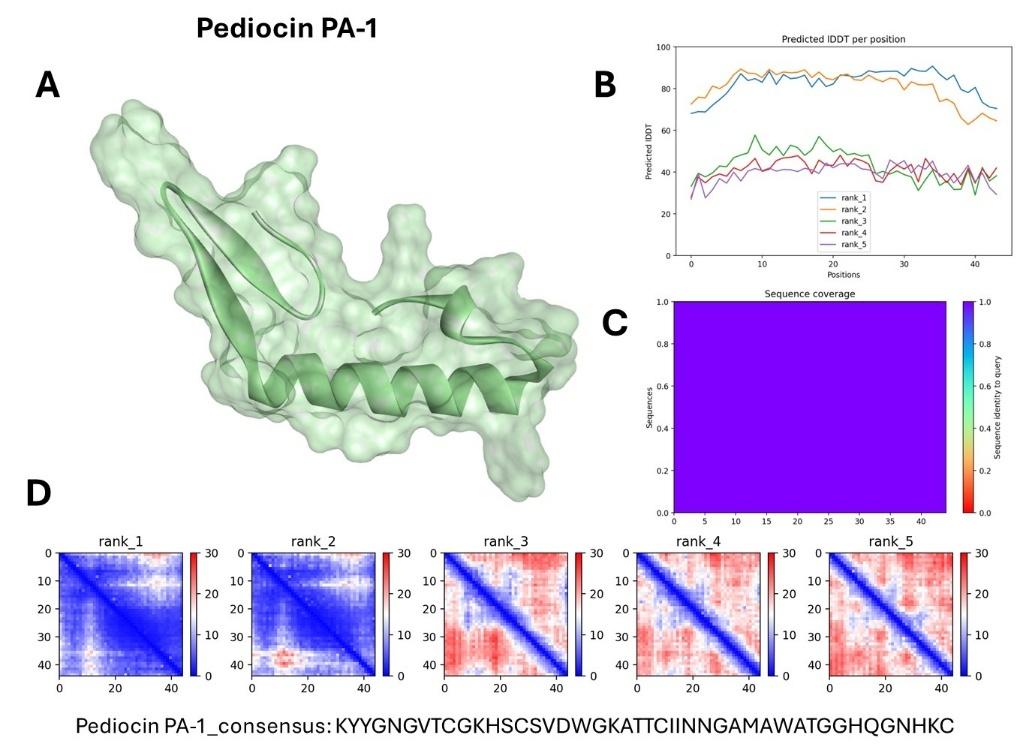


**Figure S2** – AlphaFold2 prediction and structural evaluation of the Pediocin PA-1: (A) Three-dimensional structural model of Pediocin PA-1 predicted by AlphaFold2, (B) pLDDT scores for the five best-scoring models (rank_1–rank_5), (C) Sequence coverage plot showing the multiple sequence alignment depth used during structure prediction and (D) PAE heatmaps for the five ranked models of Pediocin PA-1.


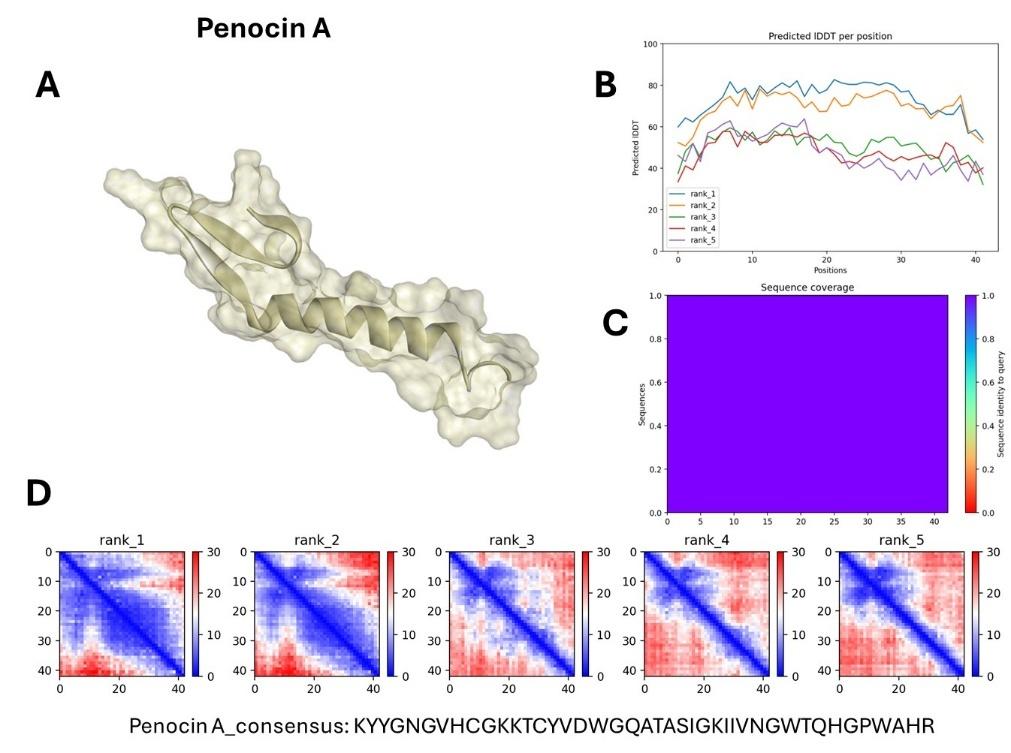


**Figure S3** – AlphaFold2 prediction and structural evaluation of the Penocin A: (A) Three-dimensional structural model of Penocin A predicted by AlphaFold2, (B) pLDDT scores for the five best-scoring models (rank_1–rank_5), (C) Sequence coverage plot showing the multiple sequence alignment depth used during structure prediction and (D) PAE heatmaps for the five ranked models of Penocin A.


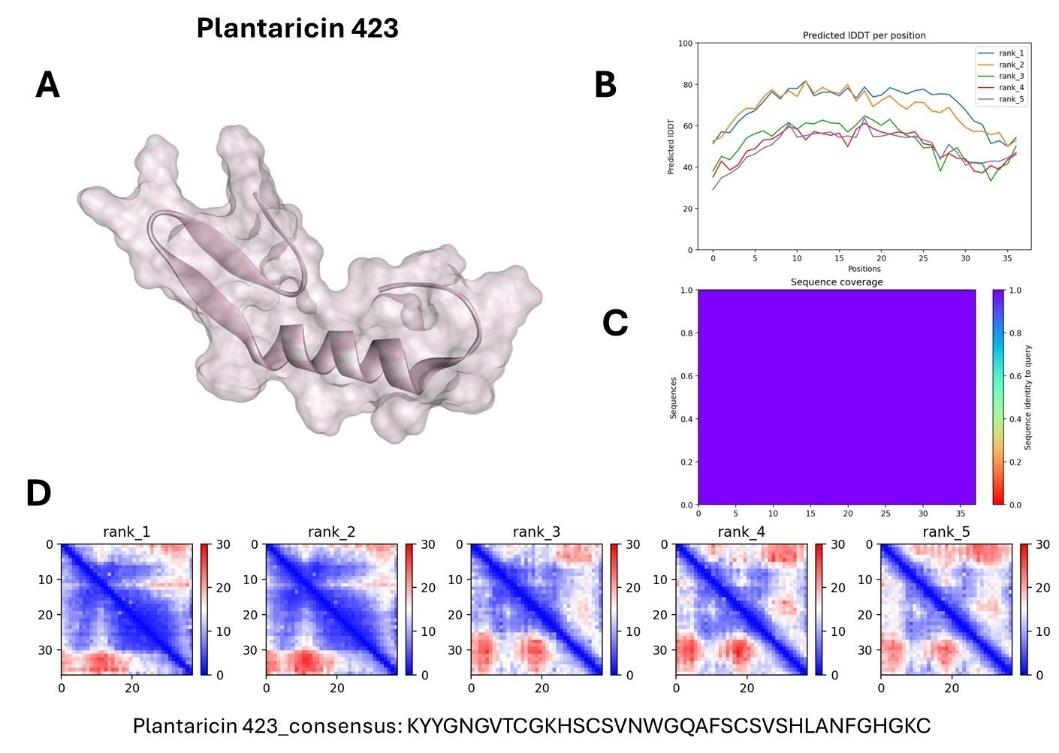


**Figure S4** – AlphaFold2 prediction and structural evaluation of the Plantaricin 423: (A) Three-dimensional structural model of Plantaricin 423 predicted by AlphaFold2, (B) pLDDT scores for the five best-scoring models (rank_1–rank_5), (C) Sequence coverage plot showing the multiple sequence alignment depth used during structure prediction and (D) PAE heatmaps for the five ranked models of Plantaricin 423.

**
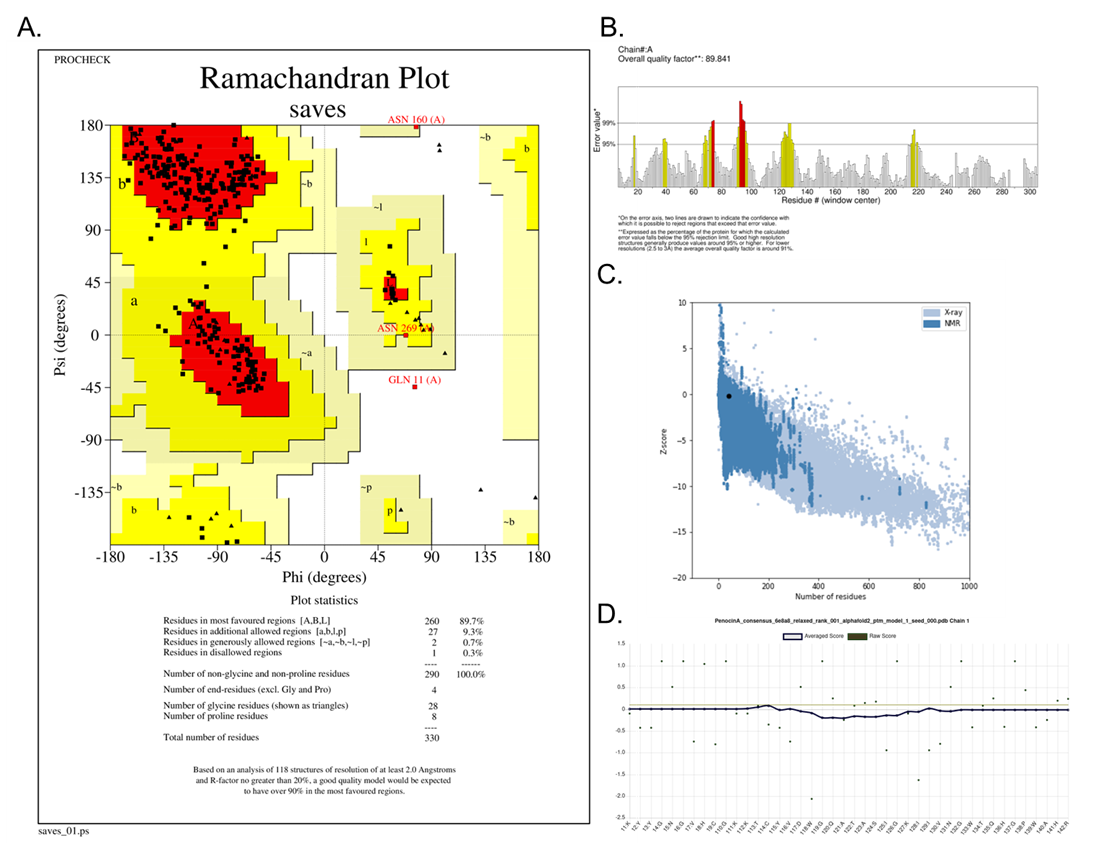
**

**Figure S5 -** Structural validation of the protonated SAP2 structure. (A) Ramachandran plot analysis generated by PROCHECK. (B) Overall quality factor determined by ERRAT. (C) Z-score estimation and local model quality assessed by ProSA-web. (D) 3D-1D profile calculated by VERIFY 3D.

**
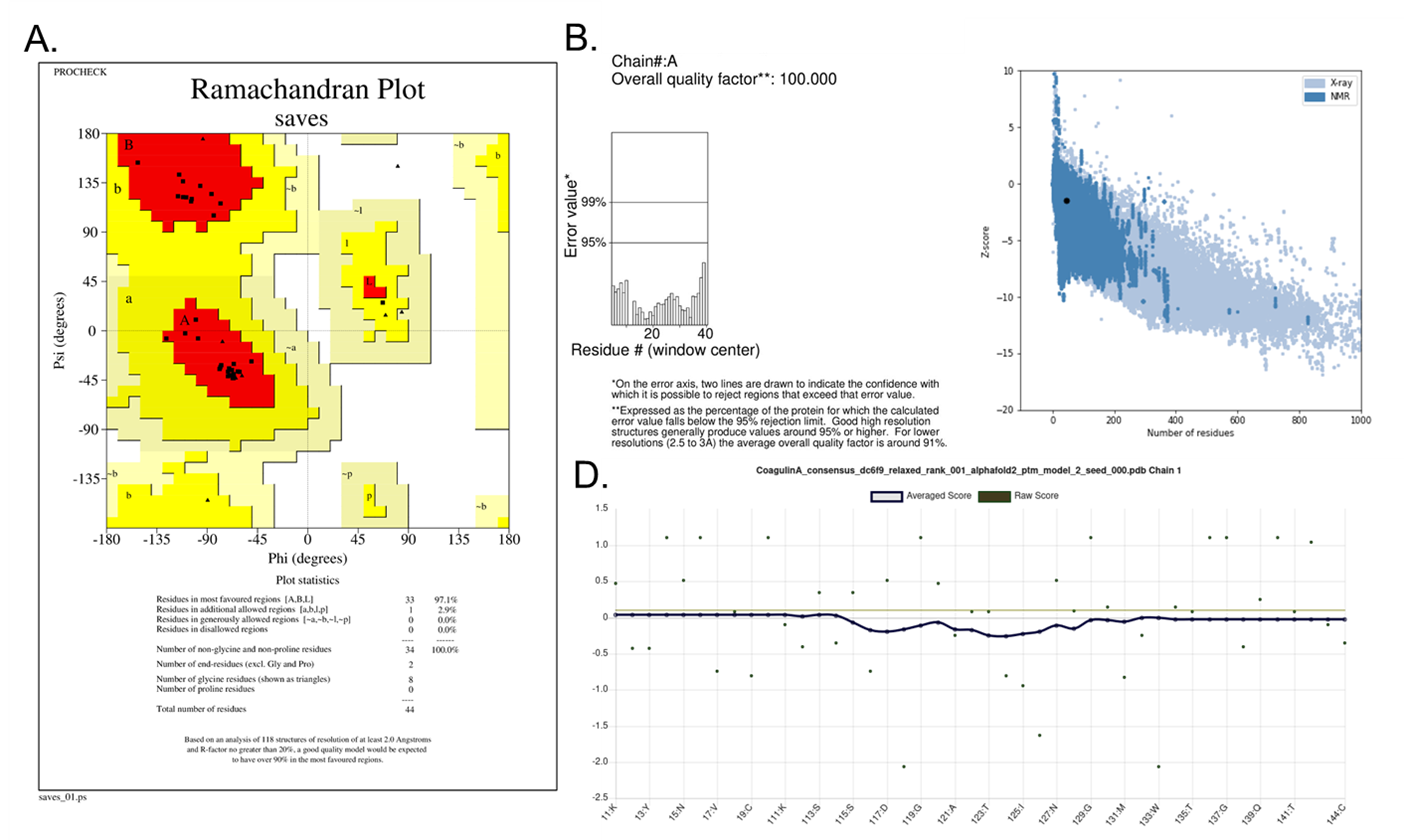
**

**Figure S6 -** Structural validation of the Coagulin A predicted model. (A) Ramachandran plot analysis generated by PROCHECK. (B) Overall quality factor determined by ERRAT. (C) Z-score estimation and local model quality assessed by ProSA-web. (D) 3D-1D profile calculated by VERIFY 3D.


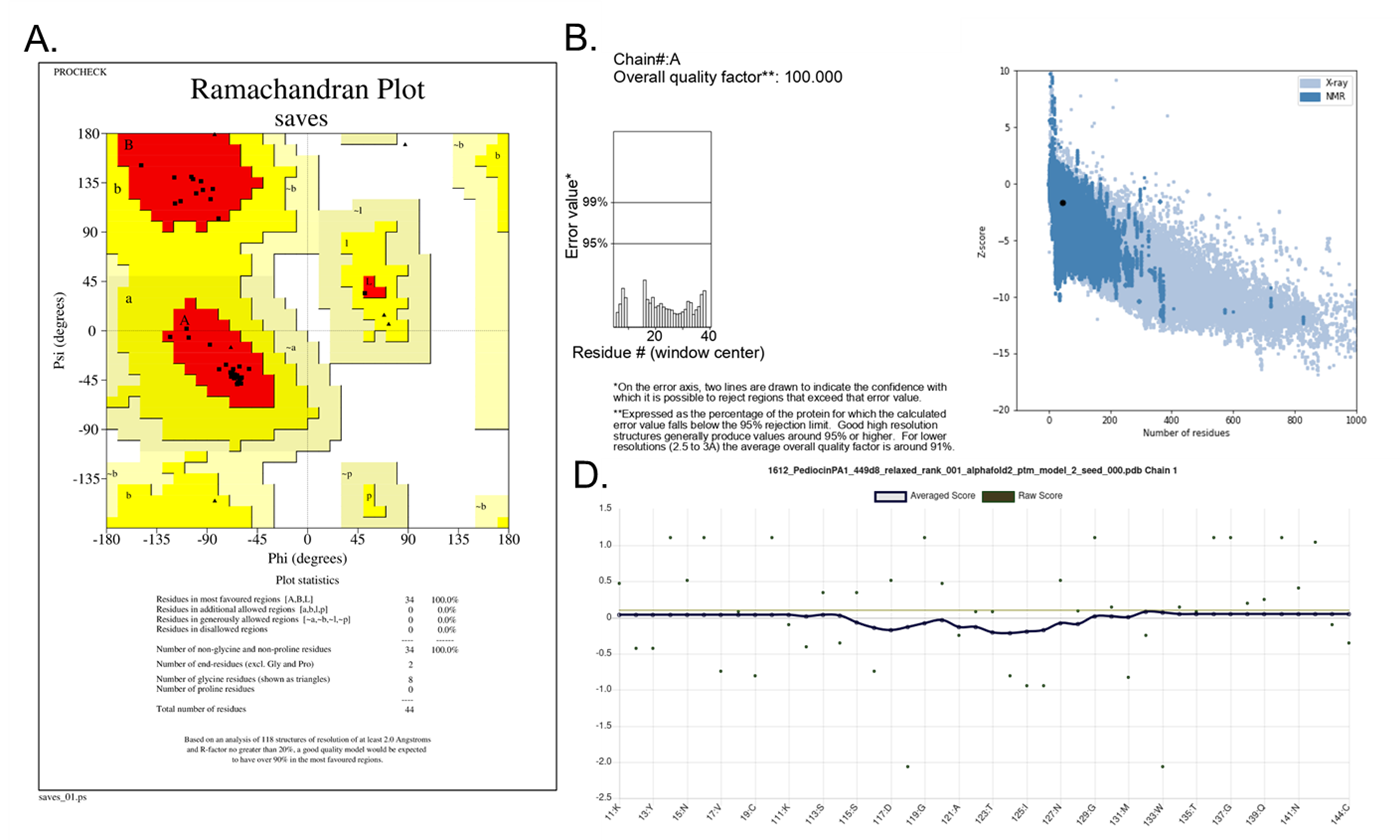


**Figure S7 -** Structural validation of the Pediocin PA-1 predicted model. (A) Ramachandran plot analysis generated by PROCHECK. (B) Overall quality factor determined by ERRAT. (C) Z-score estimation and local model quality assessed by ProSA-web. (D) 3D-1D profile calculated by VERIFY 3D.


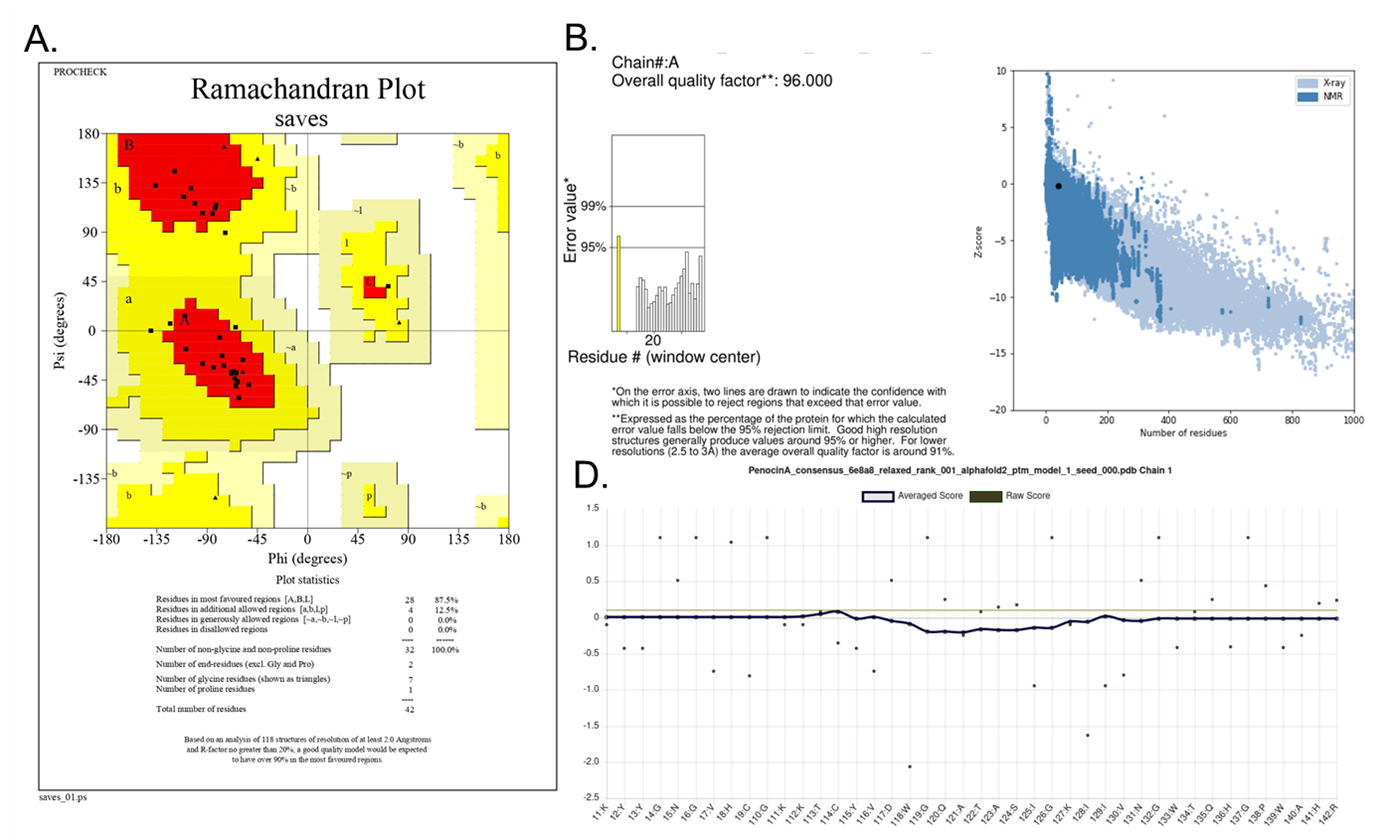


**Figure S8 -** Structural validation of the Penocin A predicted model. (A) Ramachandran plot analysis generated by PROCHECK. (B) Overall quality factor determined by ERRAT. (C) Z-score estimation and local model quality assessed by ProSA-web. (D) 3D-1D profile calculated by VERIFY 3D.


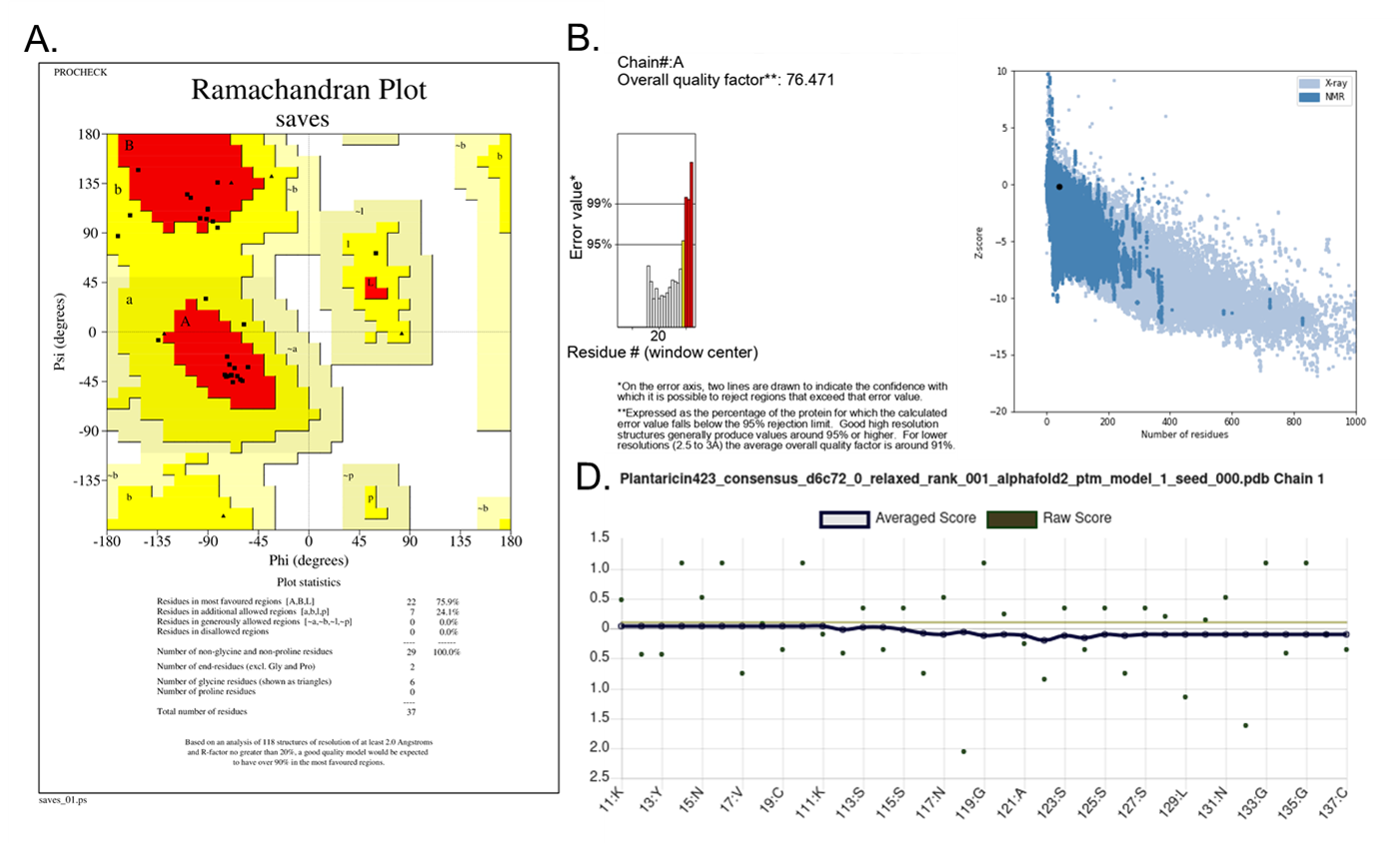


**Figure S9 -** Structural validation of the Plantaricin 423 predicted model. (A) Ramachandran plot analysis generated by PROCHECK. (B) Overall quality factor determined by ERRAT. (C) Z-score estimation and local model quality assessed by ProSA-web. (D) 3D-1D profile calculated by VERIFY 3D.

**Table S1 -** Molecular docking results of *C. albicans* SAP2 complexes with Benzamidine (BEN), Coagulin A, Pediocin PA-1, Penocin A, and Plantaricin 423 predicted using HDock.

| Peptide | Docking Score (kcal.mol^-1^) | Confidence Score | Ligand RMSD (Å) |
| --- | --- | --- | --- |
| Benzamidine (BEN) | -128.08 | 0.3921 | 0.28 |
| Coagulin A | -203.71 | 0.7454 | 58.56 |
| Pediocin PA-1 | -184.91 | 0.6678 | 43.12 |
| Penocin A | -170.96 | 0.6033 | 66.15 |
| Plantaricin 423 | -165.54 | 0.5771 | 55.98 |

**Table S2** - Molecular interactions (Hydrogen bonds, hydrophobic and electrostatic interactions) of SAP2 with the reference inhibitor and bacteriocins.

| **Ligand** | **Hydrogen Bonding** | **Electrostatic** | **Hydrophobic** |
| --- | --- | --- | --- |
| Benzamidine | ASP32 GLY34 TYR84 ASP86 ASP218 GLY220 THR221 | - | TYR225 |
| Coagulin A | ASP86 TYR225 GLN228 ASN249 SER250 TYR252 VAL300 ASN301 ASP302 | ASP218 TYR252 | TYR84  GLY85 |
| Pediocin PA-1 | ASP32 GLY85 ASP218 GLN228 ASN249 SER250 VAL300 ASN301 ASP302 ALA303 | ASP32 ASP218 GLH193 TYR252 | ILE305 |
| Penocin A | THR50 ASP86 GLY220 ASN247 LEU296 | ASP32 ASP218 ASP299 | ILE30  TYR51  TYR84  ILE223 |
| Plantaricin 423 | ASP32 ASN131 ARG192 GLU193 GLY220 GLN228 GLY248 SER250 | ASP32  ASP86 ARG192 ASP218 | - |

**Table S3 -** MM/GBSA results (kcal·mol⁻¹) of *C. albicans* SAP2 complexes with Coagulin A, Pediocin PA-1, Penocin A, and Plantaricin 423 predicted using HawkDock.

| **Peptide** | **Van der Waals (VDW)** | **Electrostatic (ELE)** | **Polar solvation (GB)** | **Nonpolar solvation (SA)** | **Binding energy** |
| --- | --- | --- | --- | --- | --- |
| Coagulin A | -91.65 | -1172.71 | 1203.09 | -13.43 | -74.69 |
| Pediocin PA-1 | -81.78 | -1468.99 | 1490.10 | -12.41 | -73.08 |
| Penocin A | -92.62 | -1395.14 | 1429.75 | -13.70 | -71.70 |
| Plantaricin 423 | -80.25 | -1005.99 | 1051.68 | -10.76 | -45.32 |

**Table S4 -** MM/GBSA results (kcal·mol⁻¹) of *C. albicans* SAP2 complexed with Coagulin A, calculated using HawkDock.

| ***C. albicans* SAP2**  **(Sequence)** | **Van der Waals (VDW)** | **Electrostatic (ELE)** | **Polar solvation (GB)** | **Nonpolar solvation (SA)** | **Binding energy** |
| --- | --- | --- | --- | --- | --- |
| ALA2 | 0.00 | 7.74 | -7.64 | 0.00 | 0.10 |
| VAL3 | 0.00 | -0.12 | 0.12 | 0.00 | 0.00 |
| PRO4 | 0.00 | 0.14 | -0.14 | 0.00 | 0.00 |
| VAL5 | 0.00 | -0.04 | 0.04 | 0.00 | 0.00 |
| THR6 | 0.00 | -0.06 | 0.07 | 0.00 | 0.00 |
| LEU7 | 0.00 | -14.84 | 14.66 | 0.00 | -0.18 |
| HIS8 | 0.00 | -18.98 | 18.76 | 0.00 | -0.22 |
| ASN9 | -0.01 | 19.92 | -19.50 | 0.00 | 0.41 |
| GLN10 | -0.04 | -0.54 | 0.62 | 0.00 | 0.04 |
| GLN11 | -0.04 | 0.94 | -0.84 | 0.00 | 0.06 |
| VAL12 | -0.02 | -1.23 | 1.20 | 0.00 | -0.05 |
| THR13 | 0.00 | 0.02 | -0.01 | 0.00 | 0.00 |
| TYR14 | 0.00 | -0.46 | 0.46 | 0.00 | -0.01 |
| ALA15 | 0.00 | -12.69 | 12.53 | 0.00 | -0.16 |
| ALA16 | 0.00 | -0.08 | 0.08 | 0.00 | 0.00 |
| ASP17 | 0.00 | -0.24 | 0.23 | 0.00 | 0.00 |
| ILE18 | 0.00 | 0.01 | 0.00 | 0.00 | 0.00 |
| THR19 | 0.00 | -0.17 | 0.16 | 0.00 | 0.00 |
| VAL20 | 0.00 | -0.08 | 0.07 | 0.00 | 0.00 |
| GLY21 | 0.00 | -0.09 | 0.09 | 0.00 | 0.00 |
| SER22 | 0.00 | -0.14 | 0.14 | 0.00 | 0.00 |
| ASN23 | 0.00 | -0.41 | 0.40 | 0.00 | -0.01 |
| ASN24 | 0.00 | 8.99 | -8.87 | 0.00 | 0.11 |
| GLN25 | 0.00 | 0.38 | -0.37 | 0.00 | 0.01 |
| LYS26 | 0.00 | -0.82 | 0.82 | 0.00 | 0.00 |
| LEU27 | 0.00 | 0.75 | -0.70 | 0.00 | 0.05 |
| ASN28 | -0.08 | 0.92 | -0.85 | -0.01 | -0.03 |
| VAL29 | -0.03 | -0.86 | 0.96 | 0.00 | 0.08 |
| ILE30 | 0.31 | -49.77 | 44.62 | -0.05 | -4.89 |
| VAL31 | -0.07 | 0.81 | -0.78 | 0.00 | -0.04 |
| ASP32 | -0.40 | 4.34 | -3.65 | -0.07 | 0.23 |
| THR33 | -0.21 | 1.11 | -1.09 | 0.00 | -0.19 |
| GLY34 | -0.01 | -0.25 | 0.24 | 0.00 | -0.03 |
| SER35 | -0.01 | -13.67 | 13.52 | 0.00 | -0.16 |
| SER36 | 0.00 | 0.28 | -0.27 | 0.00 | 0.00 |
| ASP37 | -0.02 | 0.56 | -0.56 | 0.00 | -0.01 |
| LEU38 | 0.00 | 0.07 | -0.07 | 0.00 | 0.00 |
| TRP39 | 0.00 | 0.27 | -0.27 | 0.00 | 0.00 |
| VAL40 | 0.00 | -10.15 | 10.02 | 0.00 | -0.13 |
| PRO41 | 0.00 | -0.16 | 0.16 | 0.00 | 0.00 |
| ASP42 | 0.00 | 0.03 | -0.02 | 0.00 | 0.00 |
| VAL43 | 0.00 | 0.27 | -0.26 | 0.00 | 0.00 |
| ASN44 | 0.00 | -24.60 | 24.29 | 0.00 | -0.31 |
| VAL45 | -0.01 | 15.57 | -15.27 | 0.00 | 0.29 |
| ASP46 | -0.01 | 0.33 | -0.30 | 0.00 | 0.02 |
| CYS47 | -0.06 | 0.21 | -0.06 | 0.00 | 0.10 |
| GLN48 | -0.37 | -1.31 | 1.31 | -0.22 | -0.59 |
| VAL49 | 0.00 | 0.09 | -0.07 | 0.00 | 0.01 |
| THR50 | 0.00 | -18.76 | 18.58 | 0.00 | -0.19 |
| TYR51 | -0.01 | 0.77 | -0.74 | 0.00 | 0.02 |
| SER52 | 0.00 | -0.93 | 0.92 | 0.00 | -0.01 |
| ASP53 | 0.00 | -0.15 | 0.15 | 0.00 | 0.00 |
| GLN54 | 0.00 | -11.84 | 11.69 | 0.00 | -0.15 |
| THR55 | -0.01 | -16.24 | 16.07 | 0.00 | -0.18 |
| ALA56 | 0.00 | 25.57 | -25.22 | 0.00 | 0.35 |
| ASP57 | 0.00 | 0.86 | -0.84 | 0.00 | 0.01 |
| PHE58 | 0.00 | 17.96 | -17.71 | 0.00 | 0.25 |
| CYS59 | 0.00 | -0.60 | 0.60 | 0.00 | -0.01 |
| LYS60 | 0.00 | 0.07 | -0.07 | 0.00 | 0.00 |
| GLN61 | 0.00 | -0.16 | 0.15 | 0.00 | 0.00 |
| LYS62 | 0.00 | -8.03 | 7.93 | 0.00 | -0.10 |
| GLY63 | 0.00 | 0.09 | -0.09 | 0.00 | 0.00 |
| THR64 | 0.00 | 0.05 | -0.05 | 0.00 | 0.00 |
| TYR65 | 0.00 | 0.18 | -0.18 | 0.00 | 0.00 |
| ASP66 | 0.00 | 0.14 | -0.14 | 0.00 | 0.00 |
| PRO67 | 0.00 | 0.00 | 0.00 | 0.00 | 0.00 |
| SER68 | 0.00 | 0.07 | -0.07 | 0.00 | 0.00 |
| GLY69 | 0.00 | 0.28 | -0.28 | 0.00 | 0.00 |
| SER70 | 0.00 | -0.15 | 0.15 | 0.00 | 0.00 |
| SER71 | 0.00 | -7.17 | 7.08 | 0.00 | -0.09 |
| ALA72 | 0.00 | 0.18 | -0.18 | 0.00 | 0.00 |
| SER73 | 0.00 | 0.09 | -0.09 | 0.00 | 0.00 |
| GLN74 | 0.00 | -0.01 | 0.01 | 0.00 | 0.00 |
| ASP75 | 0.00 | 0.10 | -0.09 | 0.00 | 0.00 |
| LEU76 | 0.00 | 0.18 | -0.17 | 0.00 | 0.00 |
| ASN77 | -0.01 | 10.59 | -10.41 | 0.00 | 0.18 |
| THR78 | -0.04 | -0.08 | 0.11 | 0.00 | -0.01 |
| PRO79 | -0.10 | 1.20 | -0.95 | 0.00 | 0.14 |
| PHE80 | -2.10 | -1.28 | 2.57 | -0.34 | -1.16 |
| LYS81 | -2.08 | -1.07 | 1.71 | -0.37 | -1.80 |
| ILE82 | -1.44 | -40.55 | 40.84 | -0.47 | -1.61 |
| GLY83 | -0.21 | -0.12 | 0.26 | 0.00 | -0.07 |
| TYR84 | -0.15 | 1.21 | -1.17 | 0.00 | -0.11 |
| GLY85 | -0.03 | -1.26 | 1.29 | 0.00 | 0.01 |
| ASP86 | -0.01 | -0.51 | 0.51 | 0.00 | -0.01 |
| GLY87 | 0.00 | 0.21 | -0.20 | 0.00 | 0.01 |
| SER88 | 0.00 | -0.03 | 0.03 | 0.00 | 0.00 |
| SER89 | 0.00 | -0.31 | 0.31 | 0.00 | 0.00 |
| SER90 | 0.00 | 0.15 | -0.15 | 0.00 | 0.00 |
| GLN91 | 0.00 | -0.12 | 0.12 | 0.00 | 0.00 |
| GLY92 | 0.00 | 7.04 | -6.95 | 0.00 | 0.09 |
| THR93 | 0.00 | -8.12 | 8.02 | 0.00 | -0.10 |
| LEU94 | 0.00 | -0.10 | 0.10 | 0.00 | 0.00 |
| TYR95 | 0.00 | 0.22 | -0.22 | 0.00 | 0.00 |
| LYS96 | 0.00 | -0.20 | 0.20 | 0.00 | 0.00 |
| ASP97 | 0.00 | 0.17 | -0.17 | 0.00 | 0.00 |
| THR98 | 0.00 | 0.14 | -0.14 | 0.00 | 0.00 |
| VAL99 | 0.00 | 0.14 | -0.14 | 0.00 | 0.00 |
| GLY100 | 0.00 | -0.12 | 0.12 | 0.00 | 0.00 |
| PHE101 | 0.00 | 0.00 | 0.00 | 0.00 | 0.00 |
| GLY102 | 0.00 | -0.05 | 0.05 | 0.00 | 0.00 |
| GLY103 | 0.00 | 6.08 | -6.00 | 0.00 | 0.08 |
| VAL104 | 0.00 | 0.00 | 0.00 | 0.00 | 0.00 |
| SER105 | 0.00 | 0.29 | -0.28 | 0.00 | 0.01 |
| ILE106 | 0.00 | -0.21 | 0.21 | 0.00 | 0.00 |
| LYS107 | 0.00 | 0.37 | -0.37 | 0.00 | 0.01 |
| ASN108 | 0.00 | -0.20 | 0.20 | 0.00 | 0.00 |
| GLN109 | 0.00 | -9.78 | 9.66 | 0.00 | -0.12 |
| VAL110 | 0.00 | 0.14 | -0.14 | 0.00 | 0.00 |
| LEU111 | 0.00 | -10.31 | 10.18 | 0.00 | -0.13 |
| ALA112 | 0.00 | -0.10 | 0.10 | 0.00 | 0.00 |
| ASP113 | -0.01 | 0.33 | -0.32 | 0.00 | 0.01 |
| VAL114 | -0.02 | -0.95 | 0.99 | 0.00 | 0.03 |
| ASP115 | -0.04 | 1.00 | -0.99 | 0.00 | -0.02 |
| SER116 | -0.01 | -24.33 | 24.13 | 0.00 | -0.21 |
| THR117 | -0.01 | -1.28 | 1.32 | 0.00 | 0.03 |
| SER118 | 0.00 | -0.87 | 0.87 | 0.00 | 0.00 |
| ILE119 | -0.11 | 1.18 | -1.19 | -0.02 | -0.15 |
| ASP120 | -0.01 | -0.44 | 0.45 | 0.00 | 0.00 |
| GLN121 | -0.01 | 0.71 | -0.71 | 0.00 | -0.02 |
| GLY122 | -0.01 | 0.63 | -0.62 | 0.00 | 0.00 |
| ILE123 | 0.00 | 0.09 | -0.07 | 0.00 | 0.01 |
| LEU124 | -0.01 | -0.48 | 0.47 | 0.00 | -0.01 |
| GLY125 | 0.00 | 7.22 | -7.12 | 0.00 | 0.10 |
| VAL126 | 0.00 | 0.07 | -0.06 | 0.00 | 0.01 |
| GLY127 | -0.03 | -8.70 | 8.70 | 0.00 | -0.03 |
| TYR128 | -0.01 | 11.09 | -10.87 | 0.00 | 0.21 |
| LYS129 | 0.00 | 0.27 | -0.26 | 0.00 | 0.00 |
| THR130 | 0.00 | -0.22 | 0.22 | 0.00 | 0.00 |
| ASN131 | 0.00 | 0.03 | -0.03 | 0.00 | 0.00 |
| GLN132 | 0.00 | -0.06 | 0.06 | 0.00 | 0.00 |
| ALA133 | 0.00 | -7.57 | 7.48 | 0.00 | -0.09 |
| GLY134 | 0.00 | 0.96 | -0.93 | 0.00 | 0.03 |
| GLY135 | 0.00 | 0.52 | -0.51 | 0.00 | 0.01 |
| SER136 | 0.00 | 0.36 | -0.36 | 0.00 | 0.01 |
| TYR137 | 0.00 | 0.19 | -0.19 | 0.00 | 0.00 |
| ASP138 | 0.00 | 0.34 | -0.34 | 0.00 | 0.01 |
| ASN139 | 0.00 | 0.30 | -0.29 | 0.00 | 0.01 |
| VAL140 | 0.00 | 7.04 | -6.95 | 0.00 | 0.09 |
| PRO141 | 0.00 | 7.02 | -6.93 | 0.00 | 0.09 |
| VAL142 | 0.00 | 0.02 | -0.02 | 0.00 | 0.00 |
| THR143 | 0.00 | 0.07 | -0.07 | 0.00 | 0.00 |
| LEU144 | 0.00 | 0.11 | -0.11 | 0.00 | 0.00 |
| LYS145 | 0.00 | 0.07 | -0.07 | 0.00 | 0.00 |
| LYS146 | 0.00 | 0.08 | -0.08 | 0.00 | 0.00 |
| GLN147 | 0.00 | 6.92 | -6.83 | 0.00 | 0.09 |
| GLY148 | 0.00 | -0.30 | 0.30 | 0.00 | 0.00 |
| VAL149 | 0.00 | 0.11 | -0.11 | 0.00 | 0.00 |
| ILE150 | 0.00 | 0.16 | -0.15 | 0.00 | 0.00 |
| ALA151 | 0.00 | -0.06 | 0.06 | 0.00 | 0.00 |
| LYS152 | -0.01 | 0.49 | -0.48 | 0.00 | 0.01 |
| ASN153 | 0.00 | 0.13 | -0.12 | 0.00 | 0.01 |
| ALA154 | 0.00 | 0.05 | -0.05 | 0.00 | 0.00 |
| TYR155 | 0.00 | -0.50 | 0.50 | 0.00 | -0.01 |
| SER156 | 0.00 | -0.16 | 0.16 | 0.00 | 0.00 |
| LEU157 | 0.00 | -0.28 | 0.27 | 0.00 | 0.00 |
| TYR158 | 0.00 | -11.88 | 11.74 | 0.00 | -0.15 |
| LEU159 | 0.00 | -0.50 | 0.50 | 0.00 | 0.00 |
| ASN160 | 0.00 | -0.44 | 0.44 | 0.00 | 0.00 |
| SER161 | 0.00 | 0.12 | -0.11 | 0.00 | 0.00 |
| PRO162 | 0.00 | 0.21 | -0.20 | 0.00 | 0.01 |
| ASP163 | 0.00 | -0.09 | 0.09 | 0.00 | 0.00 |
| ALA164 | 0.00 | 0.09 | -0.09 | 0.00 | 0.00 |
| ALA165 | 0.00 | -0.04 | 0.04 | 0.00 | 0.00 |
| THR166 | 0.00 | 0.10 | -0.10 | 0.00 | 0.00 |
| GLY167 | 0.00 | 0.16 | -0.15 | 0.00 | 0.00 |
| GLN168 | 0.00 | -0.17 | 0.17 | 0.00 | 0.00 |
| ILE169 | 0.00 | 0.16 | -0.15 | 0.00 | 0.00 |
| ILE170 | 0.00 | -8.54 | 8.44 | 0.00 | -0.11 |
| PHE171 | 0.00 | -0.08 | 0.08 | 0.00 | 0.00 |
| GLY172 | 0.00 | 0.08 | -0.08 | 0.00 | 0.00 |
| GLY173 | 0.00 | 9.04 | -8.93 | 0.00 | 0.11 |
| VAL174 | 0.00 | 0.10 | -0.10 | 0.00 | 0.00 |
| ASP175 | 0.00 | -0.21 | 0.20 | 0.00 | 0.00 |
| ASN176 | 0.00 | -0.04 | 0.04 | 0.00 | 0.00 |
| ALA177 | 0.00 | -0.05 | 0.05 | 0.00 | 0.00 |
| LYS178 | 0.00 | 0.23 | -0.23 | 0.00 | 0.00 |
| TYR179 | 0.00 | -0.18 | 0.18 | 0.00 | 0.00 |
| SER180 | 0.00 | 0.11 | -0.11 | 0.00 | 0.00 |
| GLY181 | 0.00 | -0.02 | 0.02 | 0.00 | 0.00 |
| SER182 | 0.00 | -0.16 | 0.15 | 0.00 | 0.00 |
| LEU183 | 0.00 | 0.18 | -0.17 | 0.00 | 0.01 |
| ILE184 | 0.00 | 0.08 | -0.07 | 0.00 | 0.01 |
| ALA185 | -0.01 | -0.02 | 0.04 | 0.00 | 0.01 |
| LEU186 | 0.00 | -6.81 | 6.74 | 0.00 | -0.07 |
| PRO187 | -0.04 | -3.86 | 3.96 | 0.00 | 0.06 |
| VAL188 | -0.01 | 9.85 | -9.53 | 0.00 | 0.31 |
| THR189 | -0.08 | 5.66 | -5.40 | 0.00 | 0.19 |
| SER190 | -0.01 | -0.11 | 0.11 | 0.00 | -0.01 |
| ASP191 | 0.00 | -0.03 | 0.04 | 0.00 | 0.01 |
| ARG192 | -0.01 | 0.18 | -0.17 | 0.00 | 0.01 |
| GLN193 | 0.00 | 0.04 | -0.03 | 0.00 | 0.00 |
| LEU194 | 0.00 | -0.08 | 0.09 | 0.00 | 0.01 |
| ARG195 | 0.00 | 0.13 | -0.12 | 0.00 | 0.00 |
| ILE196 | 0.00 | -8.79 | 8.68 | 0.00 | -0.12 |
| SER197 | 0.00 | 0.21 | -0.20 | 0.00 | 0.01 |
| LEU198 | 0.00 | 0.06 | -0.05 | 0.00 | 0.01 |
| GLY199 | 0.00 | 0.16 | -0.16 | 0.00 | 0.00 |
| SER200 | 0.00 | 8.64 | -8.50 | 0.00 | 0.13 |
| VAL201 | 0.00 | 0.10 | -0.09 | 0.00 | 0.01 |
| GLU202 | -0.01 | 0.00 | 0.00 | 0.00 | 0.00 |
| VAL203 | 0.00 | -0.34 | 0.36 | 0.00 | 0.01 |
| SER204 | 0.00 | 0.03 | -0.01 | 0.00 | 0.02 |
| GLY205 | 0.00 | -7.19 | 7.12 | 0.00 | -0.07 |
| LYS206 | 0.00 | 0.30 | -0.28 | 0.00 | 0.02 |
| THR207 | -0.01 | -0.09 | 0.10 | 0.00 | 0.01 |
| ILE208 | -0.01 | -7.71 | 7.64 | 0.00 | -0.09 |
| ASN209 | -0.02 | 0.31 | -0.27 | 0.00 | 0.03 |
| THR210 | -0.23 | -0.30 | 0.33 | -0.04 | -0.23 |
| ASP211 | -0.02 | 0.17 | -0.14 | 0.00 | 0.01 |
| ASN212 | -0.68 | -34.65 | 34.67 | -0.05 | -0.72 |
| VAL213 | -0.06 | 0.72 | -0.48 | 0.00 | 0.18 |
| ASP214 | -0.44 | -4.80 | 4.30 | -0.12 | -1.05 |
| VAL215 | -0.91 | -1.27 | 1.39 | -0.13 | -0.92 |
| LEU216 | -0.11 | 2.31 | -2.02 | 0.00 | 0.19 |
| LEU217 | -0.26 | 0.50 | -0.38 | -0.04 | -0.18 |
| ASP218 | -0.06 | -0.97 | 1.13 | 0.00 | 0.10 |
| SER219 | -1.50 | -1.52 | 1.08 | -0.38 | -2.31 |
| GLY220 | -0.12 | -1.03 | 1.02 | 0.00 | -0.13 |
| THR221 | -0.14 | -0.12 | 0.29 | 0.00 | 0.03 |
| THR222 | -2.43 | -2.14 | 3.87 | -0.45 | -1.15 |
| ILE223 | -0.06 | -9.31 | 9.29 | 0.00 | -0.08 |
| THR224 | -0.03 | 0.16 | -0.05 | 0.00 | 0.08 |
| TYR225 | -0.07 | -0.13 | 0.18 | 0.00 | -0.02 |
| LEU226 | -0.28 | -9.54 | 10.80 | -0.01 | 0.96 |
| GLN227 | -0.02 | 0.12 | -0.05 | 0.00 | 0.04 |
| GLN228 | -0.02 | 0.31 | -0.29 | 0.00 | 0.01 |
| ASP229 | -0.12 | 0.29 | -0.29 | 0.00 | -0.11 |
| LEU230 | -0.04 | 9.67 | -9.36 | 0.00 | 0.27 |
| ALA231 | 0.00 | 0.28 | -0.26 | 0.00 | 0.01 |
| ASP232 | -0.01 | 0.18 | -0.16 | 0.00 | 0.00 |
| GLN233 | -0.01 | -0.05 | 0.09 | 0.00 | 0.04 |
| ILE234 | -0.02 | 0.69 | -0.55 | 0.00 | 0.11 |
| ILE235 | -0.05 | 10.46 | -10.09 | 0.00 | 0.32 |
| LYS236 | -0.72 | 0.92 | -0.50 | -0.10 | -0.40 |
| ALA237 | -0.11 | -2.89 | 2.86 | 0.00 | -0.14 |
| PHE238 | -0.39 | 2.59 | -1.80 | -0.12 | 0.29 |
| ASN239 | -0.18 | -21.48 | 21.89 | 0.00 | 0.23 |
| GLY240 | -0.07 | 1.07 | -0.84 | 0.00 | 0.16 |
| LYS241 | -0.98 | 4.43 | -2.93 | -0.23 | 0.30 |
| LEU242 | -0.74 | -0.39 | 0.73 | -0.12 | -0.52 |
| THR243 | -2.79 | -1.08 | 3.91 | -0.52 | -0.48 |
| GLN244 | -2.17 | -2.28 | 4.35 | -0.50 | -0.60 |
| ASP245 | -0.69 | 0.23 | 0.26 | -0.07 | -0.28 |
| SER246 | -1.47 | -3.10 | 2.48 | -0.22 | -2.32 |
| ASN247 | -0.04 | -16.61 | 16.47 | 0.00 | -0.17 |
| GLY248 | -0.01 | -0.51 | 0.52 | 0.00 | -0.01 |
| ASN249 | 0.00 | -24.74 | 24.48 | 0.00 | -0.27 |
| SER250 | 0.00 | 12.12 | -11.95 | 0.00 | 0.17 |
| PHE251 | 0.00 | 0.01 | -0.01 | 0.00 | 0.00 |
| TYR252 | 0.00 | -0.11 | 0.11 | 0.00 | 0.00 |
| GLU253 | 0.00 | -0.19 | 0.19 | 0.00 | 0.00 |
| VAL254 | 0.00 | -9.67 | 9.55 | 0.00 | -0.12 |
| ASP255 | 0.00 | 0.03 | -0.02 | 0.00 | 0.00 |
| CYS256 | 0.00 | 0.01 | -0.01 | 0.00 | 0.00 |
| ASN257 | -0.01 | 0.14 | -0.14 | 0.00 | -0.01 |
| LEU258 | 0.00 | -0.24 | 0.23 | 0.00 | 0.00 |
| SER259 | 0.00 | 0.06 | -0.07 | 0.00 | -0.01 |
| GLY260 | 0.00 | -0.25 | 0.24 | 0.00 | 0.00 |
| ASP261 | 0.00 | 6.67 | -6.59 | 0.00 | 0.08 |
| VAL262 | 0.00 | -0.05 | 0.05 | 0.00 | 0.00 |
| VAL263 | 0.00 | 0.07 | -0.06 | 0.00 | 0.00 |
| PHE264 | 0.00 | 7.71 | -7.61 | 0.00 | 0.10 |
| ASN265 | 0.00 | -0.05 | 0.05 | 0.00 | 0.00 |
| PHE266 | 0.00 | -0.12 | 0.12 | 0.00 | 0.00 |
| SER267 | 0.00 | 0.21 | -0.20 | 0.00 | 0.01 |
| LYS268 | 0.00 | -0.57 | 0.56 | 0.00 | -0.01 |
| ASN269 | 0.00 | -0.64 | 0.63 | 0.00 | -0.01 |
| ALA270 | 0.00 | -16.36 | 16.16 | 0.00 | -0.21 |
| LYS271 | -0.02 | 17.21 | -16.85 | 0.00 | 0.34 |
| ILE272 | -0.01 | 0.17 | -0.15 | 0.00 | 0.01 |
| SER273 | -0.01 | 0.17 | -0.17 | 0.00 | 0.00 |
| VAL274 | -0.01 | -20.60 | 20.44 | 0.00 | -0.17 |
| PRO275 | -0.01 | -5.40 | 5.45 | 0.00 | 0.04 |
| ALA276 | -0.08 | 20.13 | -19.37 | 0.00 | 0.69 |
| SER277 | -0.06 | -1.62 | 1.75 | 0.00 | 0.07 |
| GLN278 | -0.06 | -0.03 | 0.09 | 0.00 | 0.01 |
| PHE279 | -0.08 | -0.31 | 0.42 | 0.00 | 0.03 |
| ALA280 | -1.15 | -32.27 | 33.92 | -0.18 | 0.32 |
| ALA281 | -2.30 | -2.82 | 2.66 | -0.37 | -2.83 |
| SER282 | -3.08 | -5.44 | 2.64 | -0.53 | -6.41 |
| LYS293 | -1.32 | -17.35 | 18.40 | -0.24 | -0.51 |
| CYS294 | -1.06 | -0.43 | 0.41 | -0.23 | -1.31 |
| GLN295 | -0.11 | 0.54 | -0.42 | 0.00 | 0.01 |
| LEU296 | -0.92 | -0.19 | 0.17 | -0.15 | -1.09 |
| LEU297 | -0.03 | 0.06 | -0.04 | 0.00 | -0.01 |
| PHE298 | -0.02 | 1.89 | -1.84 | 0.00 | 0.03 |
| ASP299 | -0.03 | -25.73 | 25.43 | 0.00 | -0.33 |
| VAL300 | -0.02 | 1.13 | -1.07 | 0.00 | 0.04 |
| ASN301 | -0.01 | 0.62 | -0.60 | 0.00 | 0.02 |
| ASP302 | -0.01 | 0.86 | -0.82 | 0.00 | 0.02 |
| ALA303 | -0.01 | 20.28 | -19.95 | 0.00 | 0.32 |
| ASN304 | 0.00 | 0.25 | -0.24 | 0.00 | 0.01 |
| ILE305 | 0.00 | -0.23 | 0.23 | 0.00 | 0.00 |
| LEU306 | 0.00 | 0.08 | -0.08 | 0.00 | 0.00 |
| GLY307 | 0.00 | 0.05 | -0.05 | 0.00 | 0.00 |
| ASP308 | 0.00 | 0.08 | -0.07 | 0.00 | 0.00 |
| ASN309 | -0.01 | 0.19 | -0.19 | 0.00 | -0.01 |
| PHE310 | 0.00 | -8.11 | 8.01 | 0.00 | -0.10 |
| LEU311 | 0.00 | 0.19 | -0.19 | 0.00 | 0.00 |
| ARG312 | 0.00 | -7.28 | 7.19 | 0.00 | -0.09 |
| SER313 | 0.00 | -6.66 | 6.58 | 0.00 | -0.08 |
| ALA314 | 0.00 | -7.65 | 7.56 | 0.00 | -0.09 |
| TYR315 | 0.00 | 9.19 | -9.05 | 0.00 | 0.13 |
| ILE316 | 0.00 | -0.25 | 0.25 | 0.00 | 0.00 |
| VAL317 | 0.00 | 0.27 | -0.27 | 0.00 | 0.00 |
| TYR318 | 0.00 | -0.22 | 0.22 | 0.00 | 0.00 |
| ASP319 | 0.00 | 0.23 | -0.22 | 0.00 | 0.00 |
| LEU320 | 0.00 | 0.24 | -0.23 | 0.00 | 0.00 |
| ASP321 | 0.00 | 7.79 | -7.70 | 0.00 | 0.10 |
| ASP322 | 0.00 | 0.36 | -0.36 | 0.00 | 0.00 |
| ASN323 | 0.00 | -0.15 | 0.15 | 0.00 | 0.00 |
| GLN324 | 0.00 | -0.05 | 0.05 | 0.00 | 0.00 |
| ILE325 | 0.00 | -0.25 | 0.25 | 0.00 | 0.00 |
| SER326 | 0.00 | 0.09 | -0.09 | 0.00 | 0.00 |
| LEU327 | 0.00 | 0.07 | -0.07 | 0.00 | 0.00 |
| ALA328 | 0.00 | 0.20 | -0.20 | 0.00 | 0.00 |
| GLN329 | 0.00 | -0.26 | 0.26 | 0.00 | 0.00 |
| VAL330 | 0.00 | 0.01 | -0.01 | 0.00 | 0.00 |
| LYS331 | 0.00 | -7.03 | 6.94 | 0.00 | -0.09 |
| TYR332 | 0.00 | 7.74 | -7.64 | 0.00 | 0.10 |
| THR333 | 0.00 | -0.12 | 0.12 | 0.00 | 0.00 |
| SER334 | 0.00 | 0.14 | -0.14 | 0.00 | 0.00 |
| ALA335 | 0.00 | -0.04 | 0.04 | 0.00 | 0.00 |
| SER336 | 0.00 | -0.06 | 0.07 | 0.00 | 0.00 |
| SER337 | 0.00 | -14.84 | 14.66 | 0.00 | -0.18 |
| ILE338 | 0.00 | -18.98 | 18.76 | 0.00 | -0.22 |
| SER339 | -0.01 | 19.92 | -19.50 | 0.00 | 0.41 |
| ALA340 | -0.04 | -0.54 | 0.62 | 0.00 | 0.04 |
| LEU341 | -0.04 | 0.94 | -0.84 | 0.00 | 0.06 |

**Table S5 -** MM/GBSA results (kcal·mol⁻¹) of *C. albicans* SAP2 complexed with Pediocin PA-1, calculated using HawkDock.

| ***C. albicans* SAP2**  **(Sequence)** | **Van der Waals (VDW)** | **Electrostatic (ELE)** | **Polar solvation (GB)** | **Nonpolar solvation (SA)** | **Binding energy** |
| --- | --- | --- | --- | --- | --- |
| ALA2 | 0.00 | 7.97 | -7.87 | 0.00 | 0.10 |
| VAL3 | 0.00 | -0.13 | 0.13 | 0.00 | 0.00 |
| PRO4 | 0.00 | 0.14 | -0.14 | 0.00 | 0.00 |
| VAL5 | 0.00 | -0.06 | 0.06 | 0.00 | 0.00 |
| THR6 | 0.00 | -0.07 | 0.07 | 0.00 | 0.00 |
| LEU7 | 0.00 | 0.45 | -0.44 | 0.00 | 0.01 |
| HIS8 | 0.00 | -0.56 | 0.56 | 0.00 | 0.00 |
| ASN9 | 0.00 | -0.42 | 0.43 | 0.00 | 0.01 |
| GLN10 | 0.00 | -19.59 | 19.38 | 0.00 | -0.21 |
| GLN11 | -0.01 | -0.97 | 1.00 | 0.00 | 0.02 |
| VAL12 | -0.03 | -0.86 | 0.92 | 0.00 | 0.03 |
| THR13 | -0.02 | 0.55 | -0.50 | 0.00 | 0.04 |
| TYR14 | -0.01 | -1.15 | 1.12 | 0.00 | -0.04 |
| ALA15 | 0.00 | -0.06 | 0.06 | 0.00 | 0.00 |
| ALA16 | 0.00 | -0.38 | 0.37 | 0.00 | -0.01 |
| ASP17 | 0.00 | -12.02 | 11.87 | 0.00 | -0.15 |
| ILE18 | 0.00 | -0.06 | 0.06 | 0.00 | 0.00 |
| THR19 | 0.00 | -0.24 | 0.23 | 0.00 | 0.00 |
| VAL20 | 0.00 | 0.02 | -0.02 | 0.00 | 0.00 |
| GLY21 | 0.00 | -0.15 | 0.15 | 0.00 | 0.00 |
| SER22 | 0.00 | -0.07 | 0.07 | 0.00 | 0.00 |
| ASN23 | 0.00 | -0.09 | 0.09 | 0.00 | 0.00 |
| ASN24 | 0.00 | -0.13 | 0.13 | 0.00 | 0.00 |
| GLN25 | 0.00 | -0.39 | 0.39 | 0.00 | 0.00 |
| LYS26 | 0.00 | 8.75 | -8.64 | 0.00 | 0.11 |
| LEU27 | 0.00 | 0.35 | -0.34 | 0.00 | 0.01 |
| ASN28 | 0.00 | -0.79 | 0.79 | 0.00 | 0.00 |
| VAL29 | 0.00 | 0.57 | -0.54 | 0.00 | 0.02 |
| ILE30 | -0.03 | 0.85 | -0.81 | 0.00 | 0.01 |
| VAL31 | -0.02 | -0.68 | 0.78 | 0.00 | 0.09 |
| ASP32 | 0.12 | -49.35 | 43.08 | -0.02 | -6.16 |
| THR33 | -0.09 | 1.20 | -0.88 | 0.00 | 0.24 |
| GLY34 | -0.74 | 4.14 | -2.43 | -0.08 | 0.89 |
| SER35 | -0.24 | 0.19 | -0.41 | 0.00 | -0.45 |
| SER36 | -0.01 | -0.47 | 0.40 | 0.00 | -0.08 |
| ASP37 | -0.01 | -14.44 | 14.26 | 0.00 | -0.19 |
| LEU38 | 0.00 | 0.19 | -0.18 | 0.00 | 0.00 |
| TRP39 | -0.01 | 0.46 | -0.46 | 0.00 | -0.01 |
| VAL40 | 0.00 | 0.04 | -0.04 | 0.00 | 0.00 |
| PRO41 | 0.00 | 0.27 | -0.27 | 0.00 | 0.00 |
| ASP42 | 0.00 | -10.12 | 10.00 | 0.00 | -0.13 |
| VAL43 | 0.00 | -0.14 | 0.14 | 0.00 | 0.00 |
| ASN44 | 0.00 | 0.03 | -0.03 | 0.00 | 0.00 |
| VAL45 | 0.00 | 0.29 | -0.28 | 0.00 | 0.00 |
| ASP46 | 0.00 | -24.97 | 24.66 | 0.00 | -0.31 |
| CYS47 | -0.01 | 15.83 | -15.53 | 0.00 | 0.29 |
| GLN48 | -0.01 | 0.08 | -0.04 | 0.00 | 0.02 |
| VAL49 | -0.05 | 0.43 | -0.29 | 0.00 | 0.09 |
| THR50 | -0.16 | -0.83 | 0.96 | -0.12 | -0.15 |
| TYR51 | 0.00 | 0.03 | -0.01 | 0.00 | 0.01 |
| SER52 | 0.00 | -17.28 | 17.11 | 0.00 | -0.18 |
| ASP53 | -0.01 | 0.56 | -0.54 | 0.00 | 0.02 |
| GLN54 | 0.00 | -0.89 | 0.88 | 0.00 | -0.01 |
| THR55 | 0.00 | -0.17 | 0.17 | 0.00 | 0.00 |
| ALA56 | 0.00 | -11.43 | 11.29 | 0.00 | -0.14 |
| ASP57 | -0.01 | -15.63 | 15.46 | 0.00 | -0.18 |
| PHE58 | 0.00 | 24.93 | -24.59 | 0.00 | 0.33 |
| CYS59 | 0.00 | 0.82 | -0.81 | 0.00 | 0.01 |
| LYS60 | 0.00 | 17.10 | -16.87 | 0.00 | 0.23 |
| GLN61 | 0.00 | -0.56 | 0.55 | 0.00 | -0.01 |
| LYS62 | 0.00 | 0.09 | -0.09 | 0.00 | 0.00 |
| GLY63 | 0.00 | -0.15 | 0.15 | 0.00 | 0.00 |
| THR64 | 0.00 | -7.97 | 7.87 | 0.00 | -0.10 |
| TYR65 | 0.00 | 0.10 | -0.10 | 0.00 | 0.00 |
| ASP66 | 0.00 | 0.05 | -0.05 | 0.00 | 0.00 |
| PRO67 | 0.00 | 0.18 | -0.18 | 0.00 | 0.00 |
| SER68 | 0.00 | 0.14 | -0.14 | 0.00 | 0.00 |
| GLY69 | 0.00 | 0.00 | 0.00 | 0.00 | 0.00 |
| SER70 | 0.00 | 0.06 | -0.06 | 0.00 | 0.00 |
| SER71 | 0.00 | 0.28 | -0.28 | 0.00 | 0.00 |
| ALA72 | 0.00 | -0.17 | 0.17 | 0.00 | 0.00 |
| SER73 | 0.00 | -7.37 | 7.27 | 0.00 | -0.09 |
| GLN74 | 0.00 | 0.18 | -0.17 | 0.00 | 0.00 |
| ASP75 | 0.00 | 0.09 | -0.09 | 0.00 | 0.00 |
| LEU76 | 0.00 | 0.02 | -0.01 | 0.00 | 0.00 |
| ASN77 | 0.00 | 0.09 | -0.09 | 0.00 | 0.00 |
| THR78 | -0.01 | 0.19 | -0.18 | 0.00 | 0.00 |
| PRO79 | -0.01 | 11.59 | -11.39 | 0.00 | 0.19 |
| PHE80 | -0.03 | -0.01 | 0.04 | 0.00 | 0.00 |
| LYS81 | -0.09 | 1.24 | -1.00 | 0.00 | 0.15 |
| ILE82 | -1.66 | -0.69 | 2.06 | -0.28 | -0.58 |
| GLY83 | -2.02 | -3.63 | 3.85 | -0.38 | -2.18 |
| TYR84 | -1.67 | -45.87 | 45.84 | -0.51 | -2.21 |
| GLY85 | -0.25 | -0.09 | 0.17 | 0.00 | -0.18 |
| ASP86 | -0.13 | 1.31 | -1.24 | 0.00 | -0.06 |
| GLY87 | -0.02 | -1.32 | 1.35 | 0.00 | 0.00 |
| SER88 | -0.01 | -0.64 | 0.64 | 0.00 | -0.01 |
| SER89 | 0.00 | 0.30 | -0.28 | 0.00 | 0.01 |
| SER90 | 0.00 | -0.06 | 0.06 | 0.00 | 0.00 |
| GLN91 | 0.00 | -0.31 | 0.30 | 0.00 | 0.00 |
| GLY92 | 0.00 | 0.16 | -0.16 | 0.00 | 0.00 |
| THR93 | 0.00 | -0.13 | 0.13 | 0.00 | 0.00 |
| LEU94 | 0.00 | 7.32 | -7.22 | 0.00 | 0.09 |
| TYR95 | 0.00 | -8.18 | 8.08 | 0.00 | -0.10 |
| LYS96 | 0.00 | -0.10 | 0.10 | 0.00 | 0.00 |
| ASP97 | 0.00 | 0.20 | -0.20 | 0.00 | 0.00 |
| THR98 | 0.00 | -0.19 | 0.18 | 0.00 | 0.00 |
| VAL99 | 0.00 | 0.16 | -0.15 | 0.00 | 0.00 |
| GLY100 | 0.00 | 0.12 | -0.12 | 0.00 | 0.00 |
| PHE101 | 0.00 | 0.13 | -0.13 | 0.00 | 0.00 |
| GLY102 | 0.00 | -0.10 | 0.10 | 0.00 | 0.00 |
| GLY103 | 0.00 | -0.01 | 0.01 | 0.00 | 0.00 |
| VAL104 | 0.00 | -0.03 | 0.03 | 0.00 | 0.00 |
| SER105 | 0.00 | 6.21 | -6.14 | 0.00 | 0.08 |
| ILE106 | 0.00 | -0.01 | 0.01 | 0.00 | 0.00 |
| LYS107 | 0.00 | 0.29 | -0.29 | 0.00 | 0.01 |
| ASN108 | 0.00 | -0.20 | 0.20 | 0.00 | 0.00 |
| GLN109 | 0.00 | 0.34 | -0.33 | 0.00 | 0.01 |
| VAL110 | 0.00 | -0.17 | 0.17 | 0.00 | 0.00 |
| LEU111 | 0.00 | -9.87 | 9.74 | 0.00 | -0.12 |
| ALA112 | 0.00 | 0.16 | -0.16 | 0.00 | 0.00 |
| ASP113 | 0.00 | -10.59 | 10.46 | 0.00 | -0.13 |
| VAL114 | 0.00 | -0.10 | 0.10 | 0.00 | 0.01 |
| ASP115 | -0.01 | 0.41 | -0.39 | 0.00 | 0.01 |
| SER116 | -0.02 | -0.83 | 0.88 | 0.00 | 0.03 |
| THR117 | -0.03 | 0.81 | -0.79 | 0.00 | -0.01 |
| SER118 | -0.01 | -23.03 | 22.82 | 0.00 | -0.21 |
| ILE119 | -0.01 | -1.14 | 1.17 | 0.00 | 0.02 |
| ASP120 | 0.00 | -0.68 | 0.69 | 0.00 | 0.00 |
| GLN121 | -0.04 | 0.87 | -0.87 | 0.00 | -0.03 |
| GLY122 | -0.01 | -0.48 | 0.50 | 0.00 | 0.01 |
| ILE123 | -0.01 | 0.93 | -0.90 | 0.00 | 0.02 |
| LEU124 | -0.01 | 0.80 | -0.78 | 0.00 | 0.01 |
| GLY125 | -0.01 | 0.37 | -0.33 | 0.00 | 0.03 |
| VAL126 | -0.01 | -0.59 | 0.58 | 0.00 | -0.02 |
| GLY127 | 0.00 | 7.85 | -7.74 | 0.00 | 0.11 |
| TYR128 | 0.00 | 0.09 | -0.08 | 0.00 | 0.01 |
| LYS129 | -0.04 | -9.90 | 9.91 | 0.00 | -0.03 |
| THR130 | -0.01 | 12.31 | -12.07 | 0.00 | 0.23 |
| ASN131 | 0.00 | 0.27 | -0.26 | 0.00 | 0.00 |
| GLN132 | 0.00 | -0.26 | 0.26 | 0.00 | 0.00 |
| ALA133 | 0.00 | 0.04 | -0.03 | 0.00 | 0.00 |
| GLY134 | 0.00 | -0.08 | 0.08 | 0.00 | 0.00 |
| GLY135 | 0.00 | -8.03 | 7.93 | 0.00 | -0.10 |
| SER136 | 0.00 | 1.03 | -1.00 | 0.00 | 0.03 |
| TYR137 | 0.00 | 0.52 | -0.51 | 0.00 | 0.01 |
| ASP138 | 0.00 | 0.43 | -0.42 | 0.00 | 0.01 |
| ASN139 | 0.00 | 0.21 | -0.20 | 0.00 | 0.00 |
| VAL140 | 0.00 | 0.33 | -0.33 | 0.00 | 0.00 |
| PRO141 | 0.00 | 0.32 | -0.31 | 0.00 | 0.01 |
| VAL142 | 0.00 | 7.56 | -7.46 | 0.00 | 0.09 |
| THR143 | 0.00 | 7.46 | -7.37 | 0.00 | 0.09 |
| LEU144 | 0.00 | 0.05 | -0.05 | 0.00 | 0.00 |
| LYS145 | 0.00 | 0.08 | -0.08 | 0.00 | 0.00 |
| LYS146 | 0.00 | 0.13 | -0.12 | 0.00 | 0.00 |
| GLN147 | 0.00 | 0.08 | -0.08 | 0.00 | 0.00 |
| GLY148 | 0.00 | 0.08 | -0.08 | 0.00 | 0.00 |
| VAL149 | 0.00 | 7.46 | -7.37 | 0.00 | 0.10 |
| ILE150 | 0.00 | -0.33 | 0.33 | 0.00 | 0.00 |
| ALA151 | 0.00 | 0.15 | -0.15 | 0.00 | 0.00 |
| LYS152 | 0.00 | 0.15 | -0.14 | 0.00 | 0.00 |
| ASN153 | 0.00 | -0.06 | 0.06 | 0.00 | 0.00 |
| ALA154 | 0.00 | 0.48 | -0.46 | 0.00 | 0.01 |
| TYR155 | 0.00 | 0.16 | -0.15 | 0.00 | 0.01 |
| SER156 | 0.00 | 0.16 | -0.15 | 0.00 | 0.00 |
| LEU157 | 0.00 | -0.53 | 0.53 | 0.00 | -0.01 |
| TYR158 | 0.00 | -0.16 | 0.16 | 0.00 | 0.00 |
| LEU159 | 0.00 | -0.24 | 0.24 | 0.00 | 0.00 |
| ASN160 | 0.00 | -11.46 | 11.32 | 0.00 | -0.14 |
| SER161 | 0.00 | -0.46 | 0.46 | 0.00 | -0.01 |
| PRO162 | 0.00 | -0.44 | 0.44 | 0.00 | 0.00 |
| ASP163 | 0.00 | 0.11 | -0.10 | 0.00 | 0.00 |
| ALA164 | 0.00 | 0.20 | -0.20 | 0.00 | 0.01 |
| ALA165 | 0.00 | -0.13 | 0.13 | 0.00 | 0.00 |
| THR166 | 0.00 | 0.15 | -0.15 | 0.00 | 0.00 |
| GLY167 | 0.00 | -0.06 | 0.06 | 0.00 | 0.00 |
| GLN168 | 0.00 | 0.12 | -0.12 | 0.00 | 0.00 |
| ILE169 | 0.00 | 0.18 | -0.18 | 0.00 | 0.00 |
| ILE170 | 0.00 | -0.20 | 0.20 | 0.00 | 0.00 |
| PHE171 | 0.00 | 0.17 | -0.17 | 0.00 | 0.00 |
| GLY172 | 0.00 | -8.71 | 8.60 | 0.00 | -0.11 |
| GLY173 | 0.00 | -0.11 | 0.10 | 0.00 | 0.00 |
| VAL174 | 0.00 | 0.08 | -0.08 | 0.00 | 0.00 |
| ASP175 | 0.00 | 9.11 | -8.99 | 0.00 | 0.12 |
| ASN176 | 0.00 | 0.12 | -0.12 | 0.00 | 0.00 |
| ALA177 | 0.00 | -0.24 | 0.23 | 0.00 | 0.00 |
| LYS178 | 0.00 | -0.03 | 0.04 | 0.00 | 0.00 |
| TYR179 | 0.00 | -0.05 | 0.04 | 0.00 | 0.00 |
| SER180 | 0.00 | 0.26 | -0.26 | 0.00 | 0.00 |
| GLY181 | 0.00 | -0.21 | 0.21 | 0.00 | 0.00 |
| SER182 | 0.00 | 0.14 | -0.13 | 0.00 | 0.00 |
| LEU183 | 0.00 | -0.02 | 0.02 | 0.00 | 0.00 |
| ILE184 | 0.00 | -0.20 | 0.20 | 0.00 | 0.00 |
| ALA185 | 0.00 | 0.26 | -0.25 | 0.00 | 0.01 |
| LEU186 | 0.00 | 0.10 | -0.09 | 0.00 | 0.01 |
| PRO187 | 0.00 | 0.04 | -0.02 | 0.00 | 0.02 |
| VAL188 | 0.00 | -8.01 | 7.93 | 0.00 | -0.08 |
| THR189 | -0.06 | -4.99 | 5.09 | 0.00 | 0.05 |
| SER190 | 0.00 | 0.04 | -0.02 | 0.00 | 0.02 |
| ASP191 | -0.02 | 12.97 | -12.55 | 0.00 | 0.40 |
| ARG192 | -0.10 | 9.41 | -9.07 | -0.01 | 0.23 |
| GLN193 | -0.01 | -0.23 | 0.22 | 0.00 | -0.02 |
| LEU194 | 0.00 | -0.05 | 0.06 | 0.00 | 0.01 |
| ARG195 | -0.01 | 0.18 | -0.17 | 0.00 | 0.01 |
| ILE196 | 0.00 | 0.02 | -0.02 | 0.00 | 0.00 |
| SER197 | 0.00 | -0.13 | 0.14 | 0.00 | 0.01 |
| LEU198 | 0.00 | 0.13 | -0.12 | 0.00 | 0.01 |
| GLY199 | 0.00 | -9.11 | 8.99 | 0.00 | -0.12 |
| SER200 | 0.00 | 0.19 | -0.18 | 0.00 | 0.01 |
| VAL201 | 0.00 | 0.06 | -0.05 | 0.00 | 0.01 |
| GLU202 | 0.00 | 0.17 | -0.17 | 0.00 | 0.00 |
| VAL203 | 0.00 | 8.55 | -8.42 | 0.00 | 0.13 |
| SER204 | 0.00 | 0.13 | -0.12 | 0.00 | 0.01 |
| GLY205 | -0.01 | -0.02 | 0.02 | 0.00 | 0.00 |
| LYS206 | 0.00 | -0.39 | 0.40 | 0.00 | 0.01 |
| THR207 | -0.01 | 0.02 | 0.00 | 0.00 | 0.01 |
| ILE208 | 0.00 | -7.99 | 7.91 | 0.00 | -0.08 |
| ASN209 | 0.00 | 0.31 | -0.29 | 0.00 | 0.02 |
| THR210 | -0.01 | -0.07 | 0.09 | 0.00 | 0.01 |
| ASP211 | -0.01 | -9.41 | 9.31 | 0.00 | -0.11 |
| ASN212 | -0.02 | 0.55 | -0.49 | 0.00 | 0.04 |
| VAL213 | -0.34 | 0.24 | -0.16 | -0.05 | -0.31 |
| ASP214 | -0.03 | -0.07 | 0.16 | 0.00 | 0.06 |
| VAL215 | 0.45 | -55.11 | 47.69 | -0.04 | -7.01 |
| LEU216 | -0.07 | 0.78 | -0.56 | 0.00 | 0.16 |
| LEU217 | -0.30 | -3.13 | 3.10 | -0.07 | -0.40 |
| ASP218 | -0.83 | 0.06 | 0.34 | -0.09 | -0.52 |
| SER219 | -0.08 | 1.81 | -1.61 | 0.00 | 0.12 |
| GLY220 | -0.17 | 0.16 | -0.07 | -0.01 | -0.08 |
| THR221 | -0.05 | -0.69 | 0.85 | 0.00 | 0.10 |
| THR222 | -1.21 | -1.76 | 1.14 | -0.33 | -2.16 |
| ILE223 | -0.12 | -1.18 | 1.16 | 0.00 | -0.13 |
| THR224 | -0.15 | -0.08 | 0.27 | 0.00 | 0.04 |
| TYR225 | -1.99 | -1.31 | 3.10 | -0.43 | -0.63 |
| LEU226 | -0.06 | -10.31 | 10.27 | 0.00 | -0.09 |
| GLN227 | -0.03 | 0.32 | -0.21 | 0.00 | 0.07 |
| GLN228 | -0.06 | 0.06 | 0.00 | 0.00 | 0.00 |
| ASP229 | -0.24 | -9.99 | 11.03 | -0.01 | 0.78 |
| LEU230 | -0.02 | 0.26 | -0.20 | 0.00 | 0.04 |
| ALA231 | -0.02 | 0.41 | -0.38 | 0.00 | 0.01 |
| ASP232 | -0.10 | 0.35 | -0.34 | 0.00 | -0.10 |
| GLN233 | -0.03 | 9.30 | -9.00 | 0.00 | 0.27 |
| ILE234 | 0.00 | 0.32 | -0.31 | 0.00 | 0.01 |
| ILE235 | -0.01 | 0.21 | -0.19 | 0.00 | 0.00 |
| LYS236 | -0.01 | -0.02 | 0.07 | 0.00 | 0.03 |
| ALA237 | -0.02 | 0.71 | -0.59 | 0.00 | 0.10 |
| PHE238 | -0.05 | 9.82 | -9.47 | 0.00 | 0.30 |
| ASN239 | -0.76 | 0.75 | -0.27 | -0.11 | -0.39 |
| GLY240 | -0.11 | -3.22 | 3.17 | 0.00 | -0.17 |
| LYS241 | -0.39 | 2.52 | -1.72 | -0.16 | 0.25 |
| LEU242 | -0.14 | -19.33 | 19.65 | 0.00 | 0.19 |
| THR243 | -0.05 | 1.10 | -0.90 | 0.00 | 0.14 |
| GLN244 | -0.53 | 5.36 | -4.32 | -0.15 | 0.35 |
| ASP245 | -0.47 | -0.21 | 0.63 | -0.10 | -0.14 |
| SER246 | -2.26 | 1.07 | 1.69 | -0.51 | -0.01 |
| ASN247 | -2.39 | -2.02 | 4.11 | -0.52 | -0.82 |
| GLY248 | -0.65 | -0.89 | 1.16 | -0.03 | -0.41 |
| ASN249 | -1.39 | -3.05 | 2.56 | -0.23 | -2.11 |
| SER250 | -0.03 | -15.62 | 15.49 | 0.00 | -0.16 |
| PHE251 | -0.01 | -0.46 | 0.47 | 0.00 | -0.01 |
| TYR252 | 0.00 | -23.43 | 23.18 | 0.00 | -0.26 |
| GLU253 | 0.00 | 11.51 | -11.35 | 0.00 | 0.16 |
| VAL254 | 0.00 | 0.00 | 0.00 | 0.00 | 0.00 |
| ASP255 | 0.00 | -0.12 | 0.12 | 0.00 | 0.00 |
| CYS256 | 0.00 | -0.17 | 0.17 | 0.00 | 0.00 |
| ASN257 | 0.00 | -9.73 | 9.60 | 0.00 | -0.12 |
| LEU258 | 0.00 | 0.04 | -0.04 | 0.00 | 0.00 |
| SER259 | 0.00 | 0.03 | -0.02 | 0.00 | 0.00 |
| GLY260 | -0.01 | 0.19 | -0.19 | 0.00 | -0.01 |
| ASP261 | 0.00 | -0.26 | 0.26 | 0.00 | 0.00 |
| VAL262 | 0.00 | 0.12 | -0.12 | 0.00 | -0.01 |
| VAL263 | 0.00 | -0.25 | 0.25 | 0.00 | 0.00 |
| PHE264 | 0.00 | 7.31 | -7.22 | 0.00 | 0.09 |
| ASN265 | 0.00 | -0.04 | 0.04 | 0.00 | 0.00 |
| PHE266 | 0.00 | 0.07 | -0.07 | 0.00 | 0.00 |
| SER267 | 0.00 | 8.09 | -7.99 | 0.00 | 0.10 |
| LYS268 | 0.00 | -0.07 | 0.07 | 0.00 | 0.00 |
| ASN269 | 0.00 | -0.14 | 0.14 | 0.00 | 0.00 |
| ALA270 | 0.00 | 0.20 | -0.19 | 0.00 | 0.01 |
| LYS271 | 0.00 | -0.59 | 0.58 | 0.00 | -0.01 |
| ILE272 | 0.00 | -0.61 | 0.60 | 0.00 | -0.01 |
| SER273 | 0.00 | -15.81 | 15.61 | 0.00 | -0.20 |
| VAL274 | -0.01 | 16.75 | -16.42 | 0.00 | 0.32 |
| PRO275 | 0.00 | 0.10 | -0.08 | 0.00 | 0.01 |
| ALA276 | -0.01 | 0.10 | -0.10 | 0.00 | 0.00 |
| SER277 | -0.01 | -18.57 | 18.41 | 0.00 | -0.16 |
| GLN278 | -0.01 | -4.95 | 4.99 | 0.00 | 0.04 |
| PHE279 | -0.05 | 18.83 | -18.17 | 0.00 | 0.61 |
| ALA280 | -0.05 | -1.34 | 1.44 | 0.00 | 0.06 |
| ALA281 | -0.04 | -0.17 | 0.22 | 0.00 | 0.00 |
| SER282 | -0.06 | -0.10 | 0.21 | 0.00 | 0.04 |
| LYS293 | -0.98 | -33.83 | 35.81 | -0.17 | 0.83 |
| CYS294 | -2.05 | -2.56 | 2.82 | -0.34 | -2.13 |
| GLN295 | -3.60 | -6.94 | 4.98 | -0.54 | -6.11 |
| LEU296 | -1.68 | -19.02 | 20.42 | -0.30 | -0.58 |
| LEU297 | -1.50 | -0.11 | 0.25 | -0.27 | -1.63 |
| PHE298 | -0.14 | 0.31 | -0.17 | 0.00 | 0.00 |
| ASP299 | -0.84 | 0.32 | -0.36 | -0.15 | -1.03 |
| VAL300 | -0.03 | -0.15 | 0.17 | 0.00 | -0.01 |
| ASN301 | -0.02 | 1.89 | -1.83 | 0.00 | 0.04 |
| ASP302 | -0.02 | -25.85 | 25.53 | 0.00 | -0.34 |
| ALA303 | -0.01 | 0.86 | -0.82 | 0.00 | 0.04 |
| ASN304 | -0.01 | 0.82 | -0.79 | 0.00 | 0.02 |
| ILE305 | -0.01 | 0.98 | -0.94 | 0.00 | 0.03 |
| LEU306 | -0.01 | 19.64 | -19.33 | 0.00 | 0.31 |
| GLY307 | 0.00 | 0.31 | -0.30 | 0.00 | 0.01 |
| ASP308 | 0.00 | -0.19 | 0.19 | 0.00 | 0.00 |
| ASN309 | 0.00 | 0.06 | -0.06 | 0.00 | 0.00 |
| PHE310 | 0.00 | 0.13 | -0.12 | 0.00 | 0.00 |
| LEU311 | 0.00 | 0.08 | -0.07 | 0.00 | 0.00 |
| ARG312 | -0.01 | 0.18 | -0.17 | 0.00 | 0.00 |
| SER313 | 0.00 | -8.91 | 8.80 | 0.00 | -0.11 |
| ALA314 | 0.00 | 0.19 | -0.18 | 0.00 | 0.00 |
| TYR315 | 0.00 | -7.92 | 7.82 | 0.00 | -0.10 |
| ILE316 | 0.00 | -7.26 | 7.17 | 0.00 | -0.09 |
| VAL317 | 0.00 | -8.59 | 8.49 | 0.00 | -0.10 |
| TYR318 | 0.00 | 10.39 | -10.24 | 0.00 | 0.15 |
| ASP319 | 0.00 | -0.32 | 0.32 | 0.00 | 0.00 |
| LEU320 | 0.00 | 0.29 | -0.29 | 0.00 | 0.00 |
| ASP321 | 0.00 | -0.26 | 0.25 | 0.00 | 0.00 |
| ASP322 | 0.00 | 0.18 | -0.18 | 0.00 | 0.00 |
| ASN323 | 0.00 | 0.25 | -0.24 | 0.00 | 0.00 |
| GLN324 | 0.00 | 7.88 | -7.78 | 0.00 | 0.10 |
| ILE325 | 0.00 | 0.38 | -0.37 | 0.00 | 0.00 |
| SER326 | 0.00 | -0.15 | 0.15 | 0.00 | 0.00 |
| LEU327 | 0.00 | -0.06 | 0.06 | 0.00 | 0.00 |
| ALA328 | 0.00 | -0.25 | 0.25 | 0.00 | 0.00 |
| GLN329 | 0.00 | 0.09 | -0.09 | 0.00 | 0.00 |
| VAL330 | 0.00 | 0.06 | -0.06 | 0.00 | 0.00 |
| LYS331 | 0.00 | 0.21 | -0.21 | 0.00 | 0.00 |
| TYR332 | 0.00 | -0.27 | 0.27 | 0.00 | 0.00 |
| THR333 | 0.00 | 0.01 | -0.01 | 0.00 | 0.00 |
| SER334 | 0.00 | -7.42 | 7.33 | 0.00 | -0.09 |
| ALA335 | 0.00 | 7.97 | -7.87 | 0.00 | 0.10 |
| SER336 | 0.00 | -0.13 | 0.13 | 0.00 | 0.00 |
| SER337 | 0.00 | 0.14 | -0.14 | 0.00 | 0.00 |
| ILE338 | 0.00 | -0.06 | 0.06 | 0.00 | 0.00 |
| SER339 | 0.00 | -0.07 | 0.07 | 0.00 | 0.00 |
| ALA340 | 0.00 | 0.45 | -0.44 | 0.00 | 0.01 |
| LEU341 | 0.00 | -0.56 | 0.56 | 0.00 | 0.00 |

**Table S6 -** MM/GBSA results (kcal·mol⁻¹) of *C. albicans* SAP2 complexed with Penocin A, calculated using HawkDock.

| ***C. albicans* SAP2**  **(Sequence)** | **Van der Waals (VDW)** | **Electrostatic (ELE)** | **Polar solvation (GB)** | **Nonpolar solvation (SA)** | **Binding energy** |
| --- | --- | --- | --- | --- | --- |
| ALA2 | 0.00 | 19.11 | -18.87 | 0.00 | 0.24 |
| VAL3 | 0.00 | -0.29 | 0.29 | 0.00 | 0.00 |
| PRO4 | 0.00 | 0.25 | -0.25 | 0.00 | 0.00 |
| VAL5 | 0.00 | -0.14 | 0.14 | 0.00 | 0.00 |
| THR6 | 0.00 | -0.26 | 0.27 | 0.00 | 0.01 |
| LEU7 | -0.01 | 0.85 | -0.83 | 0.00 | 0.02 |
| HIS8 | -0.01 | -0.98 | 0.98 | 0.00 | 0.00 |
| ASN9 | -0.01 | -0.51 | 0.55 | 0.00 | 0.03 |
| GLN10 | -0.04 | -36.69 | 36.40 | 0.00 | -0.33 |
| GLN11 | -0.04 | -1.59 | 1.72 | 0.00 | 0.09 |
| VAL12 | -0.34 | -1.18 | 1.52 | -0.04 | -0.04 |
| THR13 | -0.30 | 2.00 | -1.63 | -0.06 | 0.01 |
| TYR14 | -0.05 | -2.29 | 2.23 | 0.00 | -0.11 |
| ALA15 | -0.02 | 0.08 | -0.08 | 0.00 | -0.02 |
| ALA16 | 0.00 | -0.73 | 0.72 | 0.00 | -0.02 |
| ASP17 | 0.00 | -25.55 | 25.24 | 0.00 | -0.31 |
| ILE18 | 0.00 | 0.03 | -0.03 | 0.00 | 0.00 |
| THR19 | 0.00 | -0.58 | 0.58 | 0.00 | 0.00 |
| VAL20 | 0.00 | 0.12 | -0.12 | 0.00 | 0.00 |
| GLY21 | 0.00 | -0.24 | 0.24 | 0.00 | 0.00 |
| SER22 | 0.00 | -0.11 | 0.11 | 0.00 | 0.00 |
| ASN23 | 0.00 | -0.21 | 0.21 | 0.00 | 0.00 |
| ASN24 | 0.00 | -0.25 | 0.25 | 0.00 | 0.00 |
| GLN25 | 0.00 | -0.86 | 0.85 | 0.00 | -0.01 |
| LYS26 | 0.00 | 19.19 | -18.94 | 0.00 | 0.25 |
| LEU27 | 0.00 | 0.66 | -0.64 | 0.00 | 0.02 |
| ASN28 | -0.01 | -1.36 | 1.38 | 0.00 | 0.01 |
| VAL29 | -0.02 | 1.68 | -1.45 | 0.00 | 0.21 |
| ILE30 | -0.80 | 0.77 | -0.64 | -0.11 | -0.78 |
| VAL31 | -0.06 | -1.19 | 1.38 | 0.00 | 0.13 |
| ASP32 | 0.50 | -82.19 | 76.74 | -0.03 | -4.98 |
| THR33 | -0.04 | 0.84 | -0.87 | 0.00 | -0.07 |
| GLY34 | -0.10 | 1.68 | -1.39 | -0.01 | 0.18 |
| SER35 | -0.13 | 2.12 | -1.80 | 0.00 | 0.18 |
| SER36 | -0.01 | -0.23 | 0.19 | 0.00 | -0.05 |
| ASP37 | -0.02 | -40.29 | 39.81 | 0.00 | -0.49 |
| LEU38 | -0.01 | 0.22 | -0.20 | 0.00 | 0.01 |
| TRP39 | -0.09 | 1.85 | -1.78 | 0.00 | -0.02 |
| VAL40 | -0.01 | -0.24 | 0.25 | 0.00 | 0.00 |
| PRO41 | -0.01 | 0.38 | -0.38 | 0.00 | -0.01 |
| ASP42 | 0.00 | -22.41 | 22.13 | 0.00 | -0.28 |
| VAL43 | 0.00 | -0.15 | 0.15 | 0.00 | 0.00 |
| ASN44 | 0.00 | 0.24 | -0.22 | 0.00 | 0.01 |
| VAL45 | -0.01 | 0.70 | -0.68 | 0.00 | 0.01 |
| ASP46 | -0.01 | -49.50 | 48.90 | 0.00 | -0.60 |
| CYS47 | -0.07 | 29.24 | -28.23 | 0.00 | 0.93 |
| GLN48 | -0.22 | 0.16 | 0.31 | -0.02 | 0.23 |
| VAL49 | -2.33 | -2.44 | 3.14 | -0.46 | -2.09 |
| THR50 | -4.47 | -3.44 | 4.06 | -0.70 | -4.55 |
| TYR51 | -0.91 | -0.40 | 0.81 | -0.24 | -0.73 |
| SER52 | -0.12 | -27.06 | 27.19 | 0.00 | 0.01 |
| ASP53 | -0.17 | 0.55 | -0.45 | 0.00 | -0.08 |
| GLN54 | -0.01 | -1.11 | 1.14 | 0.00 | 0.02 |
| THR55 | -0.01 | -0.34 | 0.35 | 0.00 | 0.01 |
| ALA56 | -0.01 | -21.96 | 21.70 | 0.00 | -0.26 |
| ASP57 | -0.09 | -28.69 | 28.47 | 0.00 | -0.31 |
| PHE58 | -0.01 | 49.16 | -48.39 | 0.00 | 0.77 |
| CYS59 | -0.01 | 1.14 | -1.11 | 0.00 | 0.03 |
| LYS60 | -0.02 | 31.70 | -31.20 | 0.00 | 0.49 |
| GLN61 | 0.00 | -1.00 | 1.00 | 0.00 | -0.01 |
| LYS62 | 0.00 | 0.24 | -0.22 | 0.00 | 0.01 |
| GLY63 | 0.00 | -0.23 | 0.22 | 0.00 | -0.01 |
| THR64 | 0.00 | -18.18 | 17.95 | 0.00 | -0.23 |
| TYR65 | 0.00 | 0.22 | -0.22 | 0.00 | 0.00 |
| ASP66 | 0.00 | 0.09 | -0.09 | 0.00 | 0.00 |
| PRO67 | 0.00 | 0.38 | -0.37 | 0.00 | 0.00 |
| SER68 | 0.00 | 0.32 | -0.32 | 0.00 | 0.00 |
| GLY69 | 0.00 | -0.04 | 0.04 | 0.00 | 0.00 |
| SER70 | 0.00 | 0.07 | -0.07 | 0.00 | 0.00 |
| SER71 | 0.00 | 0.62 | -0.61 | 0.00 | 0.01 |
| ALA72 | 0.00 | -0.52 | 0.51 | 0.00 | -0.01 |
| SER73 | 0.00 | -18.62 | 18.38 | 0.00 | -0.23 |
| GLN74 | 0.00 | 0.38 | -0.37 | 0.00 | 0.00 |
| ASP75 | 0.00 | 0.20 | -0.20 | 0.00 | 0.00 |
| LEU76 | 0.00 | 0.19 | -0.19 | 0.00 | 0.00 |
| ASN77 | 0.00 | 0.02 | -0.02 | 0.00 | 0.00 |
| THR78 | -0.01 | 0.78 | -0.76 | 0.00 | 0.01 |
| PRO79 | -0.01 | 29.88 | -29.43 | 0.00 | 0.44 |
| PHE80 | -0.05 | 1.95 | -1.90 | 0.00 | -0.01 |
| LYS81 | -0.07 | 1.78 | -1.54 | 0.00 | 0.17 |
| ILE82 | -2.29 | -5.79 | 5.28 | -0.37 | -3.17 |
| GLY83 | -0.28 | -1.80 | 2.03 | -0.03 | -0.07 |
| TYR84 | -2.53 | -92.56 | 93.36 | -0.65 | -2.38 |
| GLY85 | -0.58 | 1.31 | -0.92 | -0.13 | -0.31 |
| ASP86 | -1.24 | 0.74 | -0.52 | -0.17 | -1.19 |
| GLY87 | -0.13 | -0.80 | 0.91 | 0.00 | -0.02 |
| SER88 | -0.04 | -1.22 | 1.21 | 0.00 | -0.04 |
| SER89 | -0.01 | 0.55 | -0.52 | 0.00 | 0.02 |
| SER90 | 0.00 | -0.22 | 0.22 | 0.00 | 0.00 |
| GLN91 | 0.00 | -0.59 | 0.58 | 0.00 | -0.01 |
| GLY92 | 0.00 | 0.53 | -0.52 | 0.00 | 0.01 |
| THR93 | 0.00 | -0.41 | 0.41 | 0.00 | -0.01 |
| LEU94 | 0.00 | 19.26 | -19.02 | 0.00 | 0.24 |
| TYR95 | 0.00 | -19.78 | 19.54 | 0.00 | -0.25 |
| LYS96 | 0.00 | -0.25 | 0.25 | 0.00 | 0.00 |
| ASP97 | 0.00 | 0.40 | -0.39 | 0.00 | 0.01 |
| THR98 | 0.00 | -0.41 | 0.40 | 0.00 | -0.01 |
| VAL99 | 0.00 | 0.26 | -0.25 | 0.00 | 0.00 |
| GLY100 | 0.00 | 0.18 | -0.18 | 0.00 | 0.00 |
| PHE101 | 0.00 | 0.27 | -0.26 | 0.00 | 0.00 |
| GLY102 | 0.00 | -0.15 | 0.15 | 0.00 | 0.00 |
| GLY103 | 0.00 | -0.03 | 0.03 | 0.00 | 0.00 |
| VAL104 | 0.00 | 0.01 | -0.01 | 0.00 | 0.00 |
| SER105 | 0.00 | 15.60 | -15.41 | 0.00 | 0.20 |
| ILE106 | 0.00 | -0.07 | 0.07 | 0.00 | 0.00 |
| LYS107 | 0.00 | 0.83 | -0.81 | 0.00 | 0.02 |
| ASN108 | 0.00 | -0.41 | 0.40 | 0.00 | 0.00 |
| GLN109 | 0.00 | 0.72 | -0.70 | 0.00 | 0.01 |
| VAL110 | 0.00 | -0.24 | 0.24 | 0.00 | 0.00 |
| LEU111 | 0.00 | -23.24 | 22.95 | 0.00 | -0.29 |
| ALA112 | -0.01 | 0.50 | -0.48 | 0.00 | 0.01 |
| ASP113 | 0.00 | -24.06 | 23.78 | 0.00 | -0.28 |
| VAL114 | -0.01 | -0.51 | 0.54 | 0.00 | 0.02 |
| ASP115 | -0.06 | 1.34 | -1.27 | 0.00 | 0.01 |
| SER116 | -0.68 | -0.37 | 1.07 | -0.07 | -0.05 |
| THR117 | -1.29 | 1.35 | -1.31 | -0.18 | -1.43 |
| SER118 | -0.28 | -35.06 | 35.47 | -0.07 | 0.06 |
| ILE119 | -0.09 | -1.24 | 1.45 | 0.00 | 0.12 |
| ASP120 | -0.03 | -0.93 | 1.05 | 0.00 | 0.09 |
| GLN121 | -0.83 | 2.68 | -2.60 | -0.07 | -0.82 |
| GLY122 | -0.03 | -0.54 | 0.60 | 0.00 | 0.03 |
| ILE123 | -0.02 | 1.81 | -1.82 | 0.00 | -0.04 |
| LEU124 | -0.01 | 1.40 | -1.38 | 0.00 | 0.01 |
| GLY125 | 0.00 | 1.28 | -1.25 | 0.00 | 0.03 |
| VAL126 | 0.00 | -1.65 | 1.62 | 0.00 | -0.03 |
| GLY127 | 0.00 | 22.17 | -21.89 | 0.00 | 0.28 |
| TYR128 | 0.00 | -0.24 | 0.24 | 0.00 | 0.00 |
| LYS129 | -0.01 | -36.99 | 36.60 | 0.00 | -0.40 |
| THR130 | -0.01 | 38.21 | -37.66 | 0.00 | 0.54 |
| ASN131 | 0.00 | 0.57 | -0.56 | 0.00 | 0.01 |
| GLN132 | 0.00 | -0.87 | 0.87 | 0.00 | -0.01 |
| ALA133 | 0.00 | 0.14 | -0.14 | 0.00 | 0.00 |
| GLY134 | 0.00 | -0.19 | 0.19 | 0.00 | 0.00 |
| GLY135 | 0.00 | -21.57 | 21.31 | 0.00 | -0.27 |
| SER136 | 0.00 | 2.61 | -2.55 | 0.00 | 0.05 |
| TYR137 | 0.00 | 1.17 | -1.14 | 0.00 | 0.03 |
| ASP138 | 0.00 | 1.05 | -1.03 | 0.00 | 0.01 |
| ASN139 | 0.00 | 0.59 | -0.58 | 0.00 | 0.01 |
| VAL140 | 0.00 | 0.71 | -0.69 | 0.00 | 0.01 |
| PRO141 | 0.00 | 0.73 | -0.72 | 0.00 | 0.01 |
| VAL142 | 0.00 | 19.90 | -19.65 | 0.00 | 0.25 |
| THR143 | 0.00 | 20.04 | -19.79 | 0.00 | 0.25 |
| LEU144 | 0.00 | 0.25 | -0.24 | 0.00 | 0.00 |
| LYS145 | 0.00 | 0.24 | -0.24 | 0.00 | 0.00 |
| LYS146 | 0.00 | 0.35 | -0.34 | 0.00 | 0.01 |
| GLN147 | 0.00 | 0.16 | -0.16 | 0.00 | 0.00 |
| GLY148 | 0.00 | 0.14 | -0.13 | 0.00 | 0.00 |
| VAL149 | 0.00 | 19.42 | -19.17 | 0.00 | 0.24 |
| ILE150 | 0.00 | -0.61 | 0.60 | 0.00 | 0.00 |
| ALA151 | 0.00 | 0.30 | -0.30 | 0.00 | 0.01 |
| LYS152 | 0.00 | 0.03 | -0.02 | 0.00 | 0.00 |
| ASN153 | 0.00 | -0.17 | 0.17 | 0.00 | 0.00 |
| ALA154 | -0.01 | 0.77 | -0.75 | 0.00 | 0.02 |
| TYR155 | 0.00 | 0.29 | -0.28 | 0.00 | 0.01 |
| SER156 | -0.01 | 0.40 | -0.39 | 0.00 | 0.01 |
| LEU157 | 0.00 | -1.07 | 1.06 | 0.00 | -0.01 |
| TYR158 | 0.00 | -0.17 | 0.18 | 0.00 | 0.01 |
| LEU159 | 0.00 | -0.22 | 0.22 | 0.00 | 0.00 |
| ASN160 | 0.00 | -24.34 | 24.04 | 0.00 | -0.30 |
| SER161 | 0.00 | -0.77 | 0.77 | 0.00 | -0.01 |
| PRO162 | 0.00 | -0.76 | 0.76 | 0.00 | -0.01 |
| ASP163 | 0.00 | 0.24 | -0.23 | 0.00 | 0.01 |
| ALA164 | 0.00 | 0.46 | -0.45 | 0.00 | 0.01 |
| ALA165 | 0.00 | -0.27 | 0.27 | 0.00 | 0.00 |
| THR166 | 0.00 | 0.43 | -0.42 | 0.00 | 0.00 |
| GLY167 | 0.00 | -0.15 | 0.15 | 0.00 | 0.00 |
| GLN168 | 0.00 | 0.37 | -0.37 | 0.00 | 0.00 |
| ILE169 | 0.00 | 0.45 | -0.44 | 0.00 | 0.01 |
| ILE170 | 0.00 | -0.47 | 0.46 | 0.00 | -0.01 |
| PHE171 | 0.00 | 0.34 | -0.34 | 0.00 | 0.00 |
| GLY172 | 0.00 | -20.09 | 19.84 | 0.00 | -0.25 |
| GLY173 | 0.00 | -0.30 | 0.29 | 0.00 | -0.01 |
| VAL174 | 0.00 | 0.17 | -0.17 | 0.00 | 0.00 |
| ASP175 | 0.00 | 20.59 | -20.34 | 0.00 | 0.26 |
| ASN176 | 0.00 | 0.29 | -0.29 | 0.00 | 0.00 |
| ALA177 | 0.00 | -0.55 | 0.54 | 0.00 | -0.01 |
| LYS178 | 0.00 | -0.02 | 0.02 | 0.00 | 0.00 |
| TYR179 | 0.00 | -0.08 | 0.08 | 0.00 | 0.00 |
| SER180 | 0.00 | 0.57 | -0.56 | 0.00 | 0.01 |
| GLY181 | 0.00 | -0.46 | 0.45 | 0.00 | 0.00 |
| SER182 | 0.00 | 0.30 | -0.29 | 0.00 | 0.01 |
| LEU183 | 0.00 | 0.03 | -0.02 | 0.00 | 0.00 |
| ILE184 | 0.00 | -0.51 | 0.51 | 0.00 | -0.01 |
| ALA185 | 0.00 | 0.76 | -0.75 | 0.00 | 0.01 |
| LEU186 | 0.00 | 0.14 | -0.13 | 0.00 | 0.00 |
| PRO187 | 0.00 | 0.37 | -0.35 | 0.00 | 0.01 |
| VAL188 | 0.00 | -27.43 | 27.10 | 0.00 | -0.34 |
| THR189 | -0.01 | 2.47 | -2.38 | 0.00 | 0.08 |
| SER190 | -0.01 | 38.86 | -38.22 | 0.00 | 0.62 |
| ASP191 | -0.02 | 48.03 | -47.27 | 0.00 | 0.74 |
| ARG192 | 0.00 | -0.96 | 0.95 | 0.00 | -0.02 |
| GLN193 | 0.00 | -0.11 | 0.12 | 0.00 | 0.00 |
| LEU194 | 0.00 | 0.21 | -0.20 | 0.00 | 0.00 |
| ARG195 | 0.00 | -0.14 | 0.13 | 0.00 | 0.00 |
| ILE196 | 0.00 | -0.27 | 0.27 | 0.00 | 0.00 |
| SER197 | 0.00 | 0.20 | -0.20 | 0.00 | 0.00 |
| LEU198 | 0.00 | -23.03 | 22.74 | 0.00 | -0.29 |
| GLY199 | 0.00 | 0.32 | -0.31 | 0.00 | 0.01 |
| SER200 | 0.00 | 0.09 | -0.08 | 0.00 | 0.00 |
| VAL201 | 0.00 | 0.37 | -0.36 | 0.00 | 0.01 |
| GLU202 | 0.00 | 22.06 | -21.77 | 0.00 | 0.28 |
| VAL203 | 0.00 | 0.33 | -0.32 | 0.00 | 0.01 |
| SER204 | 0.00 | -0.02 | 0.02 | 0.00 | 0.00 |
| GLY205 | 0.00 | -1.00 | 0.99 | 0.00 | -0.01 |
| LYS206 | 0.00 | -0.08 | 0.08 | 0.00 | 0.00 |
| THR207 | 0.00 | -24.13 | 23.83 | 0.00 | -0.30 |
| ILE208 | 0.00 | 0.52 | -0.51 | 0.00 | 0.01 |
| ASN209 | 0.00 | 0.34 | -0.33 | 0.00 | 0.01 |
| THR210 | 0.00 | -31.57 | 31.18 | 0.00 | -0.39 |
| ASP211 | -0.01 | 1.73 | -1.66 | 0.00 | 0.06 |
| ASN212 | -0.15 | 0.97 | -0.97 | -0.05 | -0.19 |
| VAL213 | -0.02 | -0.51 | 0.55 | 0.00 | 0.02 |
| ASP214 | -0.46 | -79.84 | 78.82 | -0.05 | -1.53 |
| VAL215 | -0.10 | 0.51 | -0.19 | 0.00 | 0.23 |
| LEU216 | -1.43 | -2.00 | 3.36 | -0.25 | -0.33 |
| LEU217 | -1.62 | -2.43 | 2.81 | -0.18 | -1.42 |
| ASP218 | -0.65 | 2.04 | -1.24 | -0.13 | 0.01 |
| SER219 | -0.97 | 1.10 | -0.99 | -0.13 | -0.99 |
| GLY220 | -0.07 | -0.86 | 0.96 | 0.00 | 0.03 |
| THR221 | -1.07 | -1.08 | 1.23 | -0.28 | -1.20 |
| THR222 | -0.02 | -1.91 | 1.89 | 0.00 | -0.04 |
| ILE223 | -0.01 | -1.07 | 1.07 | 0.00 | -0.01 |
| THR224 | -0.01 | -0.73 | 0.76 | 0.00 | 0.02 |
| TYR225 | 0.00 | -30.23 | 29.87 | 0.00 | -0.37 |
| LEU226 | 0.00 | 1.02 | -0.99 | 0.00 | 0.03 |
| GLN227 | -0.01 | 0.77 | -0.75 | 0.00 | 0.01 |
| GLN228 | -0.01 | -35.62 | 35.23 | 0.00 | -0.39 |
| ASP229 | 0.00 | 1.16 | -1.13 | 0.00 | 0.03 |
| LEU230 | -0.01 | 1.11 | -1.08 | 0.00 | 0.02 |
| ALA231 | -0.03 | 0.84 | -0.83 | 0.00 | -0.02 |
| ASP232 | 0.00 | 26.55 | -26.21 | 0.00 | 0.34 |
| GLN233 | 0.00 | 0.80 | -0.78 | 0.00 | 0.02 |
| ILE234 | -0.01 | 0.40 | -0.40 | 0.00 | 0.00 |
| ILE235 | 0.00 | 0.04 | -0.02 | 0.00 | 0.02 |
| LYS236 | 0.00 | 1.35 | -1.30 | 0.00 | 0.05 |
| ALA237 | -0.01 | 23.17 | -22.84 | 0.00 | 0.32 |
| PHE238 | -0.03 | 0.07 | -0.03 | 0.00 | 0.01 |
| ASN239 | -0.04 | -1.04 | 1.12 | 0.00 | 0.03 |
| GLY240 | -0.13 | 0.35 | -0.01 | 0.00 | 0.21 |
| LYS241 | -1.84 | -45.88 | 48.32 | -0.30 | 0.30 |
| LEU242 | -1.03 | -1.66 | 2.73 | -0.28 | -0.25 |
| THR243 | -3.82 | -6.26 | 8.80 | -0.86 | -2.14 |
| GLN244 | -0.24 | 0.12 | 0.04 | -0.03 | -0.11 |
| ASP245 | -2.59 | 1.57 | 0.41 | -0.47 | -1.08 |
| SER246 | -0.38 | -2.81 | 3.30 | -0.08 | 0.02 |
| ASN247 | -0.57 | 2.96 | -2.57 | -0.11 | -0.29 |
| GLY248 | -0.17 | -2.45 | 2.48 | -0.04 | -0.18 |
| ASN249 | -0.04 | -34.10 | 33.76 | 0.00 | -0.37 |
| SER250 | -0.02 | -0.79 | 0.79 | 0.00 | -0.01 |
| PHE251 | 0.00 | -54.38 | 53.75 | 0.00 | -0.64 |
| TYR252 | 0.00 | 26.49 | -26.11 | 0.00 | 0.37 |
| GLU253 | 0.00 | 0.02 | -0.01 | 0.00 | 0.00 |
| VAL254 | 0.00 | -0.26 | 0.26 | 0.00 | 0.00 |
| ASP255 | 0.00 | -0.25 | 0.25 | 0.00 | 0.00 |
| CYS256 | 0.00 | -23.39 | 23.10 | 0.00 | -0.29 |
| ASN257 | 0.00 | 0.21 | -0.20 | 0.00 | 0.01 |
| LEU258 | 0.00 | 0.01 | -0.01 | 0.00 | 0.00 |
| SER259 | 0.00 | 0.59 | -0.59 | 0.00 | 0.00 |
| GLY260 | 0.00 | -0.55 | 0.55 | 0.00 | -0.01 |
| ASP261 | 0.00 | 0.37 | -0.37 | 0.00 | 0.00 |
| VAL262 | 0.00 | -0.47 | 0.46 | 0.00 | -0.01 |
| VAL263 | 0.00 | 19.83 | -19.59 | 0.00 | 0.25 |
| PHE264 | 0.00 | -0.09 | 0.09 | 0.00 | 0.00 |
| ASN265 | 0.00 | 0.05 | -0.05 | 0.00 | 0.00 |
| PHE266 | 0.00 | 20.82 | -20.55 | 0.00 | 0.26 |
| SER267 | 0.00 | -0.24 | 0.24 | 0.00 | 0.00 |
| LYS268 | 0.00 | -0.21 | 0.21 | 0.00 | 0.00 |
| ASN269 | 0.00 | 0.32 | -0.31 | 0.00 | 0.01 |
| ALA270 | 0.00 | -1.33 | 1.32 | 0.00 | -0.02 |
| LYS271 | 0.00 | -1.42 | 1.40 | 0.00 | -0.02 |
| ILE272 | 0.00 | -35.60 | 35.15 | 0.00 | -0.46 |
| SER273 | -0.03 | 37.86 | -37.14 | 0.00 | 0.69 |
| VAL274 | -0.02 | 0.54 | -0.48 | 0.00 | 0.04 |
| PRO275 | -0.07 | -0.09 | 0.11 | 0.00 | -0.05 |
| ALA276 | -0.05 | -39.75 | 39.61 | 0.00 | -0.19 |
| SER277 | -0.02 | -9.58 | 9.63 | 0.00 | 0.03 |
| GLN278 | -1.26 | 41.38 | -38.32 | -0.38 | 1.42 |
| PHE279 | 0.08 | -10.87 | 8.67 | -0.08 | -2.20 |
| ALA280 | -0.60 | -0.27 | 0.21 | -0.09 | -0.75 |
| ALA281 | -0.13 | 0.19 | -0.07 | 0.00 | -0.01 |
| SER282 | -1.02 | -74.72 | 75.40 | -0.22 | -0.56 |
| LYS293 | -0.11 | -2.82 | 2.93 | 0.00 | 0.01 |
| CYS294 | -0.16 | 3.22 | -2.86 | -0.01 | 0.19 |
| GLN295 | -0.02 | -41.77 | 41.34 | 0.00 | -0.45 |
| LEU296 | -0.11 | 2.01 | -1.89 | -0.04 | -0.03 |
| LEU297 | -0.03 | -0.56 | 0.60 | 0.00 | 0.01 |
| PHE298 | -0.61 | 0.79 | -0.83 | -0.12 | -0.77 |
| ASP299 | -0.02 | -0.26 | 0.29 | 0.00 | 0.00 |
| VAL300 | -0.03 | 2.59 | -2.49 | 0.00 | 0.07 |
| ASN301 | -0.06 | -48.84 | 48.35 | 0.00 | -0.55 |
| ASP302 | -0.03 | 2.03 | -1.91 | 0.00 | 0.08 |
| ALA303 | -0.01 | 1.57 | -1.52 | 0.00 | 0.04 |
| ASN304 | -0.01 | 1.71 | -1.65 | 0.00 | 0.04 |
| ILE305 | -0.01 | 39.86 | -39.22 | 0.00 | 0.63 |
| LEU306 | 0.00 | 0.77 | -0.75 | 0.00 | 0.01 |
| GLY307 | 0.00 | -0.24 | 0.25 | 0.00 | 0.00 |
| ASP308 | 0.00 | 0.00 | 0.00 | 0.00 | 0.00 |
| ASN309 | 0.00 | 0.38 | -0.38 | 0.00 | 0.00 |
| PHE310 | 0.00 | 0.10 | -0.10 | 0.00 | 0.00 |
| LEU311 | -0.01 | 0.45 | -0.45 | 0.00 | -0.01 |
| ARG312 | 0.00 | -22.97 | 22.68 | 0.00 | -0.29 |
| SER313 | 0.00 | 0.22 | -0.22 | 0.00 | 0.00 |
| ALA314 | 0.00 | -21.06 | 20.79 | 0.00 | -0.26 |
| TYR315 | 0.00 | -19.44 | 19.20 | 0.00 | -0.24 |
| ILE316 | 0.00 | -23.25 | 22.97 | 0.00 | -0.29 |
| VAL317 | 0.00 | 26.74 | -26.39 | 0.00 | 0.35 |
| TYR318 | 0.00 | -0.75 | 0.75 | 0.00 | -0.01 |
| ASP319 | 0.00 | 0.59 | -0.58 | 0.00 | 0.01 |
| LEU320 | 0.00 | -0.55 | 0.54 | 0.00 | -0.01 |
| ASP321 | 0.00 | 0.22 | -0.21 | 0.00 | 0.01 |
| ASP322 | 0.00 | 0.44 | -0.43 | 0.00 | 0.01 |
| ASN323 | 0.00 | 18.38 | -18.15 | 0.00 | 0.23 |
| GLN324 | 0.00 | 0.73 | -0.72 | 0.00 | 0.01 |
| ILE325 | 0.00 | -0.31 | 0.31 | 0.00 | 0.00 |
| SER326 | 0.00 | -0.14 | 0.14 | 0.00 | 0.00 |
| LEU327 | 0.00 | -0.50 | 0.49 | 0.00 | -0.01 |
| ALA328 | 0.00 | 0.20 | -0.20 | 0.00 | 0.00 |
| GLN329 | 0.00 | 0.10 | -0.10 | 0.00 | 0.00 |
| VAL330 | 0.00 | 0.46 | -0.45 | 0.00 | 0.01 |
| LYS331 | 0.00 | -0.55 | 0.54 | 0.00 | -0.01 |
| TYR332 | 0.00 | 0.03 | -0.03 | 0.00 | 0.00 |
| THR333 | 0.00 | -18.60 | 18.36 | 0.00 | -0.23 |
| SER334 | 0.00 | 19.11 | -18.87 | 0.00 | 0.24 |
| ALA335 | 0.00 | -0.29 | 0.29 | 0.00 | 0.00 |
| SER336 | 0.00 | 0.25 | -0.25 | 0.00 | 0.00 |
| SER337 | 0.00 | -0.14 | 0.14 | 0.00 | 0.00 |
| ILE338 | 0.00 | -0.26 | 0.27 | 0.00 | 0.01 |
| SER339 | -0.01 | 0.85 | -0.83 | 0.00 | 0.02 |
| ALA340 | -0.01 | -0.98 | 0.98 | 0.00 | 0.00 |
| LEU341 | -0.01 | -0.51 | 0.55 | 0.00 | 0.03 |

**Table S7 -** MM/GBSA results (kcal·mol⁻¹) of *C. albicans* SAP2 complexed with Plantaricin 423, calculated using HawkDock.

| ***C. albicans* SAP2**  **(Sequence)** | **Van der Waals (VDW)** | **Electrostatic (ELE)** | **Polar solvation (GB)** | **Nonpolar solvation (SA)** | **Binding energy** |
| --- | --- | --- | --- | --- | --- |
| ALA2 | 0.00 | 8.05 | -7.95 | 0.00 | 0.10 |
| VAL3 | 0.00 | -0.13 | 0.13 | 0.00 | 0.00 |
| PRO4 | 0.00 | 0.13 | -0.13 | 0.00 | 0.00 |
| VAL5 | 0.00 | -0.04 | 0.05 | 0.00 | 0.00 |
| THR6 | 0.00 | -0.11 | 0.11 | 0.00 | 0.00 |
| LEU7 | 0.00 | 0.48 | -0.47 | 0.00 | 0.01 |
| HIS8 | 0.00 | -0.58 | 0.57 | 0.00 | 0.00 |
| ASN9 | 0.00 | -0.49 | 0.50 | 0.00 | 0.01 |
| GLN10 | -0.01 | -20.92 | 20.70 | 0.00 | -0.23 |
| GLN11 | -0.01 | -0.71 | 0.75 | 0.00 | 0.03 |
| VAL12 | -0.03 | -0.76 | 0.83 | 0.00 | 0.04 |
| THR13 | -0.02 | 0.78 | -0.71 | 0.00 | 0.04 |
| TYR14 | -0.01 | -1.21 | 1.18 | 0.00 | -0.04 |
| ALA15 | 0.00 | 0.00 | 0.00 | 0.00 | 0.00 |
| ALA16 | 0.00 | -0.45 | 0.44 | 0.00 | -0.01 |
| ASP17 | 0.00 | -12.54 | 12.39 | 0.00 | -0.16 |
| ILE18 | 0.00 | -0.06 | 0.06 | 0.00 | 0.00 |
| THR19 | 0.00 | -0.24 | 0.24 | 0.00 | 0.00 |
| VAL20 | 0.00 | 0.03 | -0.03 | 0.00 | 0.00 |
| GLY21 | 0.00 | -0.16 | 0.16 | 0.00 | 0.00 |
| SER22 | 0.00 | -0.07 | 0.07 | 0.00 | 0.00 |
| ASN23 | 0.00 | -0.09 | 0.09 | 0.00 | 0.00 |
| ASN24 | 0.00 | -0.15 | 0.15 | 0.00 | 0.00 |
| GLN25 | 0.00 | -0.45 | 0.44 | 0.00 | -0.01 |
| LYS26 | 0.00 | 9.10 | -8.98 | 0.00 | 0.11 |
| LEU27 | 0.00 | 0.43 | -0.43 | 0.00 | 0.01 |
| ASN28 | 0.00 | -0.97 | 0.97 | 0.00 | 0.00 |
| VAL29 | 0.00 | 0.71 | -0.68 | 0.00 | 0.03 |
| ILE30 | -0.03 | 0.96 | -0.92 | 0.00 | 0.01 |
| VAL31 | -0.02 | -0.63 | 0.73 | 0.00 | 0.09 |
| ASP32 | 0.13 | -51.56 | 45.07 | -0.03 | -6.38 |
| THR33 | -0.09 | 1.24 | -0.89 | 0.00 | 0.25 |
| GLY34 | -0.83 | 4.12 | -2.38 | -0.09 | 0.81 |
| SER35 | -0.26 | 0.31 | -0.50 | 0.00 | -0.45 |
| SER36 | -0.02 | -0.40 | 0.34 | 0.00 | -0.08 |
| ASP37 | -0.01 | -15.06 | 14.87 | 0.00 | -0.20 |
| LEU38 | 0.00 | 0.23 | -0.22 | 0.00 | 0.00 |
| TRP39 | -0.02 | 0.54 | -0.53 | 0.00 | -0.01 |
| VAL40 | 0.00 | 0.06 | -0.06 | 0.00 | 0.00 |
| PRO41 | 0.00 | 0.38 | -0.38 | 0.00 | 0.00 |
| ASP42 | 0.00 | -11.22 | 11.08 | 0.00 | -0.14 |
| VAL43 | 0.00 | -0.17 | 0.17 | 0.00 | 0.00 |
| ASN44 | 0.00 | 0.05 | -0.05 | 0.00 | 0.00 |
| VAL45 | 0.00 | 0.39 | -0.38 | 0.00 | 0.01 |
| ASP46 | 0.00 | -28.93 | 28.57 | 0.00 | -0.36 |
| CYS47 | -0.02 | 19.00 | -18.56 | 0.00 | 0.42 |
| GLN48 | -0.02 | -0.11 | 0.19 | 0.00 | 0.06 |
| VAL49 | -0.34 | 2.88 | -2.25 | -0.17 | 0.12 |
| THR50 | -0.29 | -2.00 | 2.16 | -0.14 | -0.26 |
| TYR51 | -0.01 | -0.27 | 0.29 | 0.00 | 0.01 |
| SER52 | -0.01 | -17.52 | 17.36 | 0.00 | -0.17 |
| ASP53 | -0.02 | 0.97 | -0.92 | 0.00 | 0.02 |
| GLN54 | 0.00 | -1.12 | 1.11 | 0.00 | -0.01 |
| THR55 | 0.00 | -0.24 | 0.24 | 0.00 | 0.00 |
| ALA56 | 0.00 | -12.40 | 12.25 | 0.00 | -0.16 |
| ASP57 | -0.02 | -17.88 | 17.69 | 0.00 | -0.20 |
| PHE58 | 0.00 | 28.10 | -27.71 | 0.00 | 0.39 |
| CYS59 | 0.00 | 1.05 | -1.03 | 0.00 | 0.02 |
| LYS60 | 0.00 | 18.70 | -18.44 | 0.00 | 0.26 |
| GLN61 | 0.00 | -0.67 | 0.67 | 0.00 | -0.01 |
| LYS62 | 0.00 | 0.13 | -0.13 | 0.00 | 0.00 |
| GLY63 | 0.00 | -0.19 | 0.19 | 0.00 | 0.00 |
| THR64 | 0.00 | -8.55 | 8.44 | 0.00 | -0.11 |
| TYR65 | 0.00 | 0.12 | -0.12 | 0.00 | 0.00 |
| ASP66 | 0.00 | 0.06 | -0.06 | 0.00 | 0.00 |
| PRO67 | 0.00 | 0.20 | -0.20 | 0.00 | 0.00 |
| SER68 | 0.00 | 0.15 | -0.15 | 0.00 | 0.00 |
| GLY69 | 0.00 | 0.00 | 0.00 | 0.00 | 0.00 |
| SER70 | 0.00 | 0.07 | -0.07 | 0.00 | 0.00 |
| SER71 | 0.00 | 0.32 | -0.31 | 0.00 | 0.00 |
| ALA72 | 0.00 | -0.18 | 0.17 | 0.00 | 0.00 |
| SER73 | 0.00 | -7.87 | 7.77 | 0.00 | -0.10 |
| GLN74 | 0.00 | 0.20 | -0.20 | 0.00 | 0.00 |
| ASP75 | 0.00 | 0.10 | -0.10 | 0.00 | 0.00 |
| LEU76 | 0.00 | 0.01 | -0.01 | 0.00 | 0.00 |
| ASN77 | 0.00 | 0.14 | -0.14 | 0.00 | 0.01 |
| THR78 | -0.01 | 0.17 | -0.17 | 0.00 | 0.00 |
| PRO79 | -0.01 | 12.81 | -12.57 | 0.00 | 0.22 |
| PHE80 | -0.04 | -0.25 | 0.29 | 0.00 | 0.00 |
| LYS81 | -0.10 | 1.82 | -1.54 | 0.00 | 0.18 |
| ILE82 | -1.76 | -0.23 | 1.93 | -0.27 | -0.34 |
| GLY83 | -1.63 | -4.21 | 3.65 | -0.41 | -2.61 |
| TYR84 | -2.40 | -51.23 | 52.82 | -0.52 | -1.33 |
| GLY85 | -0.97 | -0.05 | 0.83 | -0.20 | -0.38 |
| ASP86 | -0.62 | 2.78 | -2.50 | -0.11 | -0.44 |
| GLY87 | -0.06 | -2.35 | 2.36 | 0.00 | -0.05 |
| SER88 | -0.01 | -1.05 | 1.04 | 0.00 | -0.02 |
| SER89 | -0.01 | 0.41 | -0.39 | 0.00 | 0.02 |
| SER90 | 0.00 | -0.09 | 0.09 | 0.00 | 0.00 |
| GLN91 | 0.00 | -0.38 | 0.37 | 0.00 | 0.00 |
| GLY92 | 0.00 | 0.16 | -0.15 | 0.00 | 0.00 |
| THR93 | 0.00 | -0.13 | 0.13 | 0.00 | 0.00 |
| LEU94 | 0.00 | 7.64 | -7.55 | 0.00 | 0.10 |
| TYR95 | 0.00 | -8.67 | 8.56 | 0.00 | -0.11 |
| LYS96 | 0.00 | -0.13 | 0.12 | 0.00 | 0.00 |
| ASP97 | 0.00 | 0.22 | -0.21 | 0.00 | 0.00 |
| THR98 | 0.00 | -0.21 | 0.21 | 0.00 | 0.00 |
| VAL99 | 0.00 | 0.16 | -0.16 | 0.00 | 0.00 |
| GLY100 | 0.00 | 0.13 | -0.13 | 0.00 | 0.00 |
| PHE101 | 0.00 | 0.14 | -0.14 | 0.00 | 0.00 |
| GLY102 | 0.00 | -0.11 | 0.11 | 0.00 | 0.00 |
| GLY103 | 0.00 | 0.00 | 0.00 | 0.00 | 0.00 |
| VAL104 | 0.00 | -0.03 | 0.03 | 0.00 | 0.00 |
| SER105 | 0.00 | 6.43 | -6.35 | 0.00 | 0.08 |
| ILE106 | 0.00 | -0.02 | 0.02 | 0.00 | 0.00 |
| LYS107 | 0.00 | 0.31 | -0.31 | 0.00 | 0.01 |
| ASN108 | 0.00 | -0.20 | 0.20 | 0.00 | 0.00 |
| GLN109 | 0.00 | 0.39 | -0.38 | 0.00 | 0.01 |
| VAL110 | 0.00 | -0.20 | 0.19 | 0.00 | 0.00 |
| LEU111 | 0.00 | -10.82 | 10.69 | 0.00 | -0.14 |
| ALA112 | 0.00 | 0.22 | -0.21 | 0.00 | 0.00 |
| ASP113 | 0.00 | -11.88 | 11.74 | 0.00 | -0.14 |
| VAL114 | 0.00 | -0.10 | 0.12 | 0.00 | 0.01 |
| ASP115 | -0.01 | 0.64 | -0.59 | 0.00 | 0.04 |
| SER116 | -0.16 | -2.56 | 2.67 | -0.04 | -0.09 |
| THR117 | -0.07 | 0.73 | -0.76 | 0.00 | -0.10 |
| SER118 | -0.02 | -28.00 | 27.78 | 0.00 | -0.23 |
| ILE119 | -0.01 | -1.46 | 1.50 | 0.00 | 0.02 |
| ASP120 | 0.00 | -0.80 | 0.80 | 0.00 | 0.00 |
| GLN121 | -0.04 | 0.91 | -0.90 | 0.00 | -0.03 |
| GLY122 | -0.01 | -0.48 | 0.50 | 0.00 | 0.01 |
| ILE123 | -0.01 | 0.90 | -0.86 | 0.00 | 0.03 |
| LEU124 | -0.01 | 0.78 | -0.76 | 0.00 | 0.02 |
| GLY125 | -0.01 | 0.30 | -0.26 | 0.00 | 0.03 |
| VAL126 | -0.01 | -0.59 | 0.58 | 0.00 | -0.02 |
| GLY127 | 0.00 | 8.03 | -7.92 | 0.00 | 0.11 |
| TYR128 | 0.00 | 0.11 | -0.10 | 0.00 | 0.01 |
| LYS129 | -0.05 | -9.61 | 9.63 | 0.00 | -0.02 |
| THR130 | -0.01 | 12.59 | -12.33 | 0.00 | 0.25 |
| ASN131 | 0.00 | 0.28 | -0.28 | 0.00 | 0.00 |
| GLN132 | 0.00 | -0.28 | 0.29 | 0.00 | 0.00 |
| ALA133 | 0.00 | 0.03 | -0.03 | 0.00 | 0.00 |
| GLY134 | 0.00 | -0.07 | 0.07 | 0.00 | 0.00 |
| GLY135 | 0.00 | -8.27 | 8.17 | 0.00 | -0.10 |
| SER136 | 0.00 | 1.06 | -1.03 | 0.00 | 0.03 |
| TYR137 | 0.00 | 0.55 | -0.53 | 0.00 | 0.01 |
| ASP138 | 0.00 | 0.43 | -0.42 | 0.00 | 0.01 |
| ASN139 | 0.00 | 0.22 | -0.22 | 0.00 | 0.00 |
| VAL140 | 0.00 | 0.36 | -0.35 | 0.00 | 0.01 |
| PRO141 | 0.00 | 0.32 | -0.32 | 0.00 | 0.01 |
| VAL142 | 0.00 | 7.64 | -7.54 | 0.00 | 0.10 |
| THR143 | 0.00 | 7.65 | -7.55 | 0.00 | 0.10 |
| LEU144 | 0.00 | 0.04 | -0.04 | 0.00 | 0.00 |
| LYS145 | 0.00 | 0.09 | -0.09 | 0.00 | 0.00 |
| LYS146 | 0.00 | 0.14 | -0.13 | 0.00 | 0.00 |
| GLN147 | 0.00 | 0.07 | -0.07 | 0.00 | 0.00 |
| GLY148 | 0.00 | 0.08 | -0.08 | 0.00 | 0.00 |
| VAL149 | 0.00 | 7.48 | -7.38 | 0.00 | 0.10 |
| ILE150 | 0.00 | -0.34 | 0.34 | 0.00 | 0.00 |
| ALA151 | 0.00 | 0.13 | -0.13 | 0.00 | 0.00 |
| LYS152 | 0.00 | 0.15 | -0.15 | 0.00 | 0.00 |
| ASN153 | 0.00 | -0.09 | 0.09 | 0.00 | 0.00 |
| ALA154 | 0.00 | 0.50 | -0.48 | 0.00 | 0.01 |
| TYR155 | 0.00 | 0.11 | -0.10 | 0.00 | 0.00 |
| SER156 | 0.00 | 0.14 | -0.13 | 0.00 | 0.00 |
| LEU157 | 0.00 | -0.46 | 0.46 | 0.00 | 0.00 |
| TYR158 | 0.00 | -0.23 | 0.24 | 0.00 | 0.00 |
| LEU159 | 0.00 | -0.29 | 0.29 | 0.00 | 0.00 |
| ASN160 | 0.00 | -10.91 | 10.78 | 0.00 | -0.14 |
| SER161 | 0.00 | -0.44 | 0.44 | 0.00 | 0.00 |
| PRO162 | 0.00 | -0.47 | 0.46 | 0.00 | -0.01 |
| ASP163 | 0.00 | 0.16 | -0.16 | 0.00 | 0.01 |
| ALA164 | 0.00 | 0.19 | -0.19 | 0.00 | 0.01 |
| ALA165 | 0.00 | -0.08 | 0.09 | 0.00 | 0.00 |
| THR166 | 0.00 | 0.15 | -0.15 | 0.00 | 0.00 |
| GLY167 | 0.00 | -0.05 | 0.05 | 0.00 | 0.00 |
| GLN168 | 0.00 | 0.13 | -0.13 | 0.00 | 0.00 |
| ILE169 | 0.00 | 0.18 | -0.18 | 0.00 | 0.00 |
| ILE170 | 0.00 | -0.20 | 0.20 | 0.00 | 0.00 |
| PHE171 | 0.00 | 0.16 | -0.16 | 0.00 | 0.00 |
| GLY172 | 0.00 | -8.62 | 8.51 | 0.00 | -0.11 |
| GLY173 | 0.00 | -0.09 | 0.09 | 0.00 | 0.00 |
| VAL174 | 0.00 | 0.09 | -0.09 | 0.00 | 0.00 |
| ASP175 | 0.00 | 9.02 | -8.91 | 0.00 | 0.11 |
| ASN176 | 0.00 | 0.11 | -0.11 | 0.00 | 0.00 |
| ALA177 | 0.00 | -0.24 | 0.23 | 0.00 | 0.00 |
| LYS178 | 0.00 | -0.04 | 0.04 | 0.00 | 0.00 |
| TYR179 | 0.00 | -0.03 | 0.03 | 0.00 | 0.00 |
| SER180 | 0.00 | 0.26 | -0.25 | 0.00 | 0.00 |
| GLY181 | 0.00 | -0.20 | 0.20 | 0.00 | 0.00 |
| SER182 | 0.00 | 0.13 | -0.13 | 0.00 | 0.00 |
| LEU183 | 0.00 | -0.02 | 0.02 | 0.00 | 0.00 |
| ILE184 | 0.00 | -0.19 | 0.19 | 0.00 | 0.00 |
| ALA185 | 0.00 | 0.25 | -0.24 | 0.00 | 0.01 |
| LEU186 | 0.00 | 0.11 | -0.09 | 0.00 | 0.01 |
| PRO187 | 0.00 | 0.03 | -0.01 | 0.00 | 0.02 |
| VAL188 | 0.00 | -7.93 | 7.85 | 0.00 | -0.08 |
| THR189 | -0.07 | -5.77 | 5.88 | 0.00 | 0.04 |
| SER190 | -0.02 | 12.61 | -12.18 | 0.00 | 0.41 |
| ASP191 | -0.12 | 9.28 | -8.96 | -0.03 | 0.17 |
| ARG192 | -0.01 | -0.23 | 0.22 | 0.00 | -0.01 |
| GLN193 | 0.00 | -0.04 | 0.05 | 0.00 | 0.01 |
| LEU194 | 0.00 | 0.11 | -0.10 | 0.00 | 0.00 |
| ARG195 | 0.00 | -0.03 | 0.04 | 0.00 | 0.00 |
| ILE196 | 0.00 | -0.11 | 0.12 | 0.00 | 0.00 |
| SER197 | 0.00 | 0.09 | -0.08 | 0.00 | 0.00 |
| LEU198 | 0.00 | -8.60 | 8.49 | 0.00 | -0.11 |
| GLY199 | 0.00 | 0.11 | -0.11 | 0.00 | 0.00 |
| SER200 | 0.00 | 0.04 | -0.04 | 0.00 | 0.00 |
| VAL201 | 0.00 | 0.15 | -0.15 | 0.00 | 0.00 |
| GLU202 | 0.00 | 7.83 | -7.72 | 0.00 | 0.11 |
| VAL203 | 0.00 | 0.13 | -0.12 | 0.00 | 0.01 |
| SER204 | 0.00 | 0.01 | -0.01 | 0.00 | 0.00 |
| GLY205 | 0.00 | -0.41 | 0.42 | 0.00 | 0.01 |
| LYS206 | 0.00 | -0.18 | 0.19 | 0.00 | 0.01 |
| THR207 | 0.00 | -8.52 | 8.43 | 0.00 | -0.09 |
| ILE208 | 0.00 | 0.26 | -0.24 | 0.00 | 0.02 |
| ASN209 | -0.01 | 0.01 | 0.01 | 0.00 | 0.01 |
| THR210 | -0.01 | -9.67 | 9.56 | 0.00 | -0.11 |
| ASP211 | -0.02 | 0.52 | -0.47 | 0.00 | 0.03 |
| ASN212 | -0.39 | 0.29 | -0.22 | -0.06 | -0.38 |
| VAL213 | -0.03 | -0.13 | 0.22 | 0.00 | 0.06 |
| ASP214 | 0.26 | -54.12 | 47.56 | -0.04 | -6.35 |
| VAL215 | -0.07 | 0.82 | -0.61 | 0.00 | 0.14 |
| LEU216 | -0.32 | -3.25 | 3.24 | -0.06 | -0.39 |
| LEU217 | -0.76 | -0.81 | 0.95 | -0.08 | -0.70 |
| ASP218 | -0.08 | 1.25 | -1.10 | 0.00 | 0.07 |
| SER219 | -0.31 | -0.75 | 0.81 | -0.06 | -0.31 |
| GLY220 | -0.05 | -0.29 | 0.41 | 0.00 | 0.07 |
| THR221 | -1.16 | 1.54 | -0.97 | -0.22 | -0.81 |
| THR222 | -0.08 | -0.96 | 0.95 | 0.00 | -0.09 |
| ILE223 | -0.10 | 0.35 | -0.22 | 0.00 | 0.04 |
| THR224 | -0.82 | -0.70 | 1.63 | -0.23 | -0.13 |
| TYR225 | -0.03 | -12.27 | 12.19 | 0.00 | -0.11 |
| LEU226 | -0.02 | 0.46 | -0.38 | 0.00 | 0.06 |
| GLN227 | -0.04 | 0.64 | -0.58 | 0.00 | 0.02 |
| GLN228 | -0.08 | -16.74 | 16.99 | 0.00 | 0.17 |
| ASP229 | -0.01 | 0.59 | -0.54 | 0.00 | 0.04 |
| LEU230 | -0.01 | 0.49 | -0.47 | 0.00 | 0.01 |
| ALA231 | -0.05 | 0.50 | -0.49 | 0.00 | -0.04 |
| ASP232 | -0.01 | 10.42 | -10.18 | 0.00 | 0.23 |
| GLN233 | 0.00 | 0.32 | -0.31 | 0.00 | 0.01 |
| ILE234 | -0.01 | 0.22 | -0.20 | 0.00 | 0.00 |
| ILE235 | 0.00 | 0.09 | -0.06 | 0.00 | 0.03 |
| LYS236 | -0.01 | 0.37 | -0.31 | 0.00 | 0.05 |
| ALA237 | -0.02 | 7.65 | -7.46 | 0.00 | 0.17 |
| PHE238 | -0.19 | 0.36 | -0.21 | -0.04 | -0.07 |
| ASN239 | -0.06 | -0.27 | 0.38 | 0.00 | 0.05 |
| GLY240 | -0.56 | -0.58 | 1.58 | -0.21 | 0.23 |
| LYS241 | -0.21 | -13.64 | 14.10 | 0.00 | 0.24 |
| LEU242 | -0.62 | -1.54 | 2.44 | -0.22 | 0.06 |
| THR243 | -1.60 | 1.92 | -0.21 | -0.21 | -0.10 |
| GLN244 | -1.67 | -0.86 | 1.03 | -0.26 | -1.75 |
| ASP245 | -1.83 | 0.84 | 1.16 | -0.41 | -0.24 |
| SER246 | -1.13 | 0.77 | 0.65 | -0.35 | -0.06 |
| ASN247 | -0.25 | 0.84 | -0.67 | -0.01 | -0.09 |
| GLY248 | -0.25 | -0.81 | 1.00 | -0.05 | -0.11 |
| ASN249 | -0.02 | -11.26 | 11.21 | 0.00 | -0.07 |
| SER250 | -0.01 | -0.22 | 0.23 | 0.00 | 0.00 |
| PHE251 | 0.00 | -19.06 | 18.84 | 0.00 | -0.21 |
| TYR252 | 0.00 | 9.65 | -9.52 | 0.00 | 0.13 |
| GLU253 | 0.00 | -0.06 | 0.06 | 0.00 | 0.00 |
| VAL254 | 0.00 | -0.11 | 0.11 | 0.00 | 0.00 |
| ASP255 | 0.00 | -0.15 | 0.15 | 0.00 | 0.00 |
| CYS256 | 0.00 | -8.90 | 8.78 | 0.00 | -0.11 |
| ASN257 | 0.00 | 0.05 | -0.04 | 0.00 | 0.00 |
| LEU258 | 0.00 | 0.03 | -0.03 | 0.00 | 0.00 |
| SER259 | -0.01 | 0.19 | -0.19 | 0.00 | -0.01 |
| GLY260 | 0.00 | -0.24 | 0.24 | 0.00 | 0.00 |
| ASP261 | 0.00 | 0.12 | -0.12 | 0.00 | 0.00 |
| VAL262 | 0.00 | -0.23 | 0.22 | 0.00 | 0.00 |
| VAL263 | 0.00 | 7.27 | -7.18 | 0.00 | 0.09 |
| PHE264 | 0.00 | -0.04 | 0.04 | 0.00 | 0.00 |
| ASN265 | 0.00 | 0.06 | -0.06 | 0.00 | 0.00 |
| PHE266 | 0.00 | 7.80 | -7.70 | 0.00 | 0.10 |
| SER267 | 0.00 | -0.05 | 0.05 | 0.00 | 0.00 |
| LYS268 | 0.00 | -0.17 | 0.17 | 0.00 | 0.00 |
| ASN269 | 0.00 | 0.17 | -0.16 | 0.00 | 0.01 |
| ALA270 | 0.00 | -0.49 | 0.49 | 0.00 | -0.01 |
| LYS271 | 0.00 | -0.46 | 0.45 | 0.00 | -0.01 |
| ILE272 | 0.00 | -13.61 | 13.44 | 0.00 | -0.17 |
| SER273 | -0.01 | 14.35 | -14.06 | 0.00 | 0.28 |
| VAL274 | -0.01 | -0.06 | 0.07 | 0.00 | 0.01 |
| PRO275 | -0.01 | 0.25 | -0.24 | 0.00 | 0.00 |
| ALA276 | -0.01 | -15.49 | 15.38 | 0.00 | -0.12 |
| SER277 | -0.01 | -3.51 | 3.57 | 0.00 | 0.05 |
| GLN278 | -0.06 | 13.61 | -13.05 | 0.00 | 0.50 |
| PHE279 | -0.05 | -0.60 | 0.68 | 0.00 | 0.04 |
| ALA280 | -0.06 | -0.23 | 0.26 | 0.00 | -0.03 |
| ALA281 | -0.06 | 0.19 | -0.14 | 0.00 | -0.01 |
| SER282 | -0.05 | -31.42 | 31.61 | -0.21 | -0.07 |
| LYS293 | -1.20 | -0.44 | 1.23 | -0.27 | -0.67 |
| CYS294 | -2.61 | -3.74 | 4.27 | -0.52 | -2.59 |
| GLN295 | -0.47 | -27.33 | 26.39 | -0.32 | -1.73 |
| LEU296 | -1.34 | 0.84 | -0.71 | -0.25 | -1.47 |
| LEU297 | -0.12 | 0.24 | -0.15 | 0.00 | -0.03 |
| PHE298 | -0.89 | 0.43 | -0.48 | -0.15 | -1.09 |
| ASP299 | -0.03 | -0.34 | 0.36 | 0.00 | -0.01 |
| VAL300 | -0.02 | 1.73 | -1.67 | 0.00 | 0.04 |
| ASN301 | -0.02 | -25.71 | 25.41 | 0.00 | -0.33 |
| ASP302 | -0.01 | 0.45 | -0.41 | 0.00 | 0.03 |
| ALA303 | -0.01 | 0.81 | -0.78 | 0.00 | 0.02 |
| ASN304 | -0.01 | 0.94 | -0.91 | 0.00 | 0.03 |
| ILE305 | -0.01 | 18.36 | -18.07 | 0.00 | 0.29 |
| LEU306 | 0.00 | 0.26 | -0.25 | 0.00 | 0.01 |
| GLY307 | 0.00 | -0.17 | 0.17 | 0.00 | 0.00 |
| ASP308 | 0.00 | 0.08 | -0.07 | 0.00 | 0.00 |
| ASN309 | 0.00 | 0.12 | -0.12 | 0.00 | 0.00 |
| PHE310 | 0.00 | 0.09 | -0.08 | 0.00 | 0.00 |
| LEU311 | -0.01 | 0.18 | -0.18 | 0.00 | 0.00 |
| ARG312 | 0.00 | -8.94 | 8.83 | 0.00 | -0.11 |
| SER313 | 0.00 | 0.18 | -0.17 | 0.00 | 0.00 |
| ALA314 | 0.00 | -7.95 | 7.85 | 0.00 | -0.10 |
| TYR315 | 0.00 | -7.26 | 7.17 | 0.00 | -0.09 |
| ILE316 | 0.00 | -8.57 | 8.47 | 0.00 | -0.10 |
| VAL317 | 0.00 | 10.29 | -10.14 | 0.00 | 0.15 |
| TYR318 | 0.00 | -0.32 | 0.32 | 0.00 | 0.00 |
| ASP319 | 0.00 | 0.27 | -0.27 | 0.00 | 0.01 |
| LEU320 | 0.00 | -0.26 | 0.25 | 0.00 | -0.01 |
| ASP321 | 0.00 | 0.14 | -0.14 | 0.00 | 0.00 |
| ASP322 | 0.00 | 0.25 | -0.24 | 0.00 | 0.00 |
| ASN323 | 0.00 | 7.65 | -7.55 | 0.00 | 0.09 |
| GLN324 | 0.00 | 0.39 | -0.39 | 0.00 | 0.00 |
| ILE325 | 0.00 | -0.16 | 0.15 | 0.00 | 0.00 |
| SER326 | 0.00 | -0.06 | 0.06 | 0.00 | 0.00 |
| LEU327 | 0.00 | -0.25 | 0.25 | 0.00 | 0.00 |
| ALA328 | 0.00 | 0.10 | -0.10 | 0.00 | 0.00 |
| GLN329 | 0.00 | 0.06 | -0.06 | 0.00 | 0.00 |
| VAL330 | 0.00 | 0.21 | -0.21 | 0.00 | 0.00 |
| LYS331 | 0.00 | -0.27 | 0.26 | 0.00 | 0.00 |
| TYR332 | 0.00 | 0.01 | -0.01 | 0.00 | 0.00 |
| THR333 | 0.00 | -7.50 | 7.40 | 0.00 | -0.09 |
| SER334 | 0.00 | 8.05 | -7.95 | 0.00 | 0.10 |
| ALA335 | 0.00 | -0.13 | 0.13 | 0.00 | 0.00 |
| SER336 | 0.00 | 0.13 | -0.13 | 0.00 | 0.00 |
| SER337 | 0.00 | -0.04 | 0.05 | 0.00 | 0.00 |
| ILE338 | 0.00 | -0.11 | 0.11 | 0.00 | 0.00 |
| SER339 | 0.00 | 0.48 | -0.47 | 0.00 | 0.01 |
| ALA340 | 0.00 | -0.58 | 0.57 | 0.00 | 0.00 |
| LEU341 | 0.00 | -0.49 | 0.50 | 0.00 | 0.01 |
